# 3D Printed Low‐Tortuosity and Ultra‐Thick Hierarchical Porous Electrodes for High‐Performance Wearable Quasi‐Solid‐State Zn‐VOH Batteries

**DOI:** 10.1002/advs.202401660

**Published:** 2025-03-05

**Authors:** Qingguo Xu, Ningning Chu, Ye Wang, Hui Wang, Tingting Xu, Xueliang Li, Shaozhuan Huang, Xinjian Li, Yongsong Luo, Hui Ying Yang, Dezhi Kong

**Affiliations:** ^1^ Key Laboratory of Material Physics of Ministry of Education School of Physics and Microelectronics Zhengzhou University Zhengzhou 450052 China; ^2^ Pillar of Engineering Product Development Singapore University of Technology and Design 8 Somapah Road Singapore 487372 Singapore; ^3^ Hubei Key Laboratory of Catalysis and Materials Science South‐Central University for Nationalities Wuhan Hubei 430074 China; ^4^ Henan International Joint Laboratory of MXene Materials Microstructure College of Physics and Electronic Engineering Nanyang Normal University Nanyang 473061 China

**Keywords:** aqueous Zn‐VOH battery, 3D printed, long‐term cycling durability, rGO/CNTs‐based microlattices electrodes, ultrahigh areal energy density

## Abstract

Rechargeable aqueous Zn‐ion batteries have received considerable attention in energy storage systems owing to their merits of high safety, low cost, and excellent rate performance. However, the unsatisfactory areal energy density and poor cycling performance hinder their practical applications. Herein, the V_5_O_12_·6H_2_O (VOH) nanosheet arrays and Zn nanoflake arrays growing on the 3D‐printed reduced graphene oxide/carbon nanotubes (3DP‐rGO/CNTs) microlattices employing the electrodeposition technique, and further serve as the cathode and anode for 3D‐printed aqueous Zn‐VOH battery, respectively. Benefiting from 3D‐printed low‐tortuosity and ultra‐thick hierarchical porous electrodes, the battery‐type VOH‐based cathode enables fast Zn^2+^ reaction kinetics and electrodeposited Zn‐based anode delivers highly reversible Zn stripping/plating. The button‐type 3D‐printed aqueous Zn‐VOH battery exhibits excellent energy/power densities (364.5 Wh kg^−1^ at 700 W kg^−1^) and remarkable cycling performance over 6500 cycles. Impressively, a customizable 3D‐printed quasi‐solid‐state Zn‐VOH battery device is fabricated, which presents ultrahigh areal capacity (≈17.06 mAh cm^−2^ at 0.2 mA cm^−2^), long‐term durability (≈85.6% capacity retention after 10 000 cycles), and excellent wearable characters. This work provides a novel strategy to gain high‐performance Zn‐ion batteries based on 3D‐printed electrodes, which may pave a new way for the applications of various high‐performance, low‐cost, and portable integrated energy storage systems.

## Graphical abstract

This work presents an advanced 3D‐printed aqueous Zn‐VOH batteries system, and the important role of 3D‐printed low‐tortuosity structures in improving thick electrodes’ electrochemical kinetics (mass transfer and charge transfer) is highlighted. The promising potential of 3D‐printed quasi‐solid‐state Zn‐VOH battery devices in many practical wearable applications and provides a new platform for integrated safe and wearable energy storage technologies.

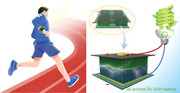

## 1 Introduction

With the rapid development of micro‐electronics technology, various kinds of micro‐electronic devices have emerged, such as imitation flying insect robots, wearable devices, micro‐drones, etc. These new‐fashioned application carriers have put forward higher requirements for miniaturization, intelligence, personalization, and integration of power source batteries.^[^ ^1^, ^2^, ^3^ ^]^ However, the traditional electrode coating manufacturing process of Li‐/Na‐/K‐ion batteries is difficult to meet the corresponding new requirements, which greatly limits the design freedom of microelectronic devices, and it is difficult to meet the high safety requirements of customized mobile electronic devices and customized wearable devices.^[^ ^4^, ^5^, ^6^, ^7^ ^]^ Rechargeable aqueous Zn‐ion batteries are receiving increasing attention because of their advantages of high safety, friendly environment, high volume energy density, and low cost.^[^ ^8^, ^9^ ^]^ Unfortunately, the development and application of Zn‐ion batteries have been severely restricted owing to the decay of cathode material performance, dendrite growth and side reactions on Zn anode, lower areal capacity, restricted shape factor.^[^ ^10^, ^11^, ^12^ ^]^ At present, the electrode thickness of planar solid‐state Zn‐ion micro‐batteries is generally small (<10 µm), which makes it difficult to meet the demand for high area capacity and energy density in practical applications. Therefore, constructing solid‐state Zn‐ion micro‐batteries with 3D structure and thick electrodes (>100 µm) is of great importance in basic science and application.^[^ ^13^, ^14^ ^]^ However, the preparation of thick electrodes shows some issues, such as high curvature, long ion diffusion paths, and insufficient utilization of electrode materials, making it difficult to realize the overall improvement of the performance of Zn‐ion micro‐batteries.^[^ ^15^, ^16^, ^17^ ^]^ In addition, the accurate design and reliable construction of the electrode structure is of great importance to achieve corrosion and dendrite‐free Zn anode.^[^ ^18^, ^19^ ^]^ Therefore, the optimization of the size, structure and spatial arrangement of the electrodes by advanced fabrication techniques such as highly ordered spatial structure and micro‐/nano‐structural modulation is expected to open up new ways for Zn‐ion batteries with high‐performance.

To fully realize the maximum potential of electrochemical properties of electrode materials, it is particularly important to develop 3D spatial ordered electrodes to solve the charge transport limitations in thick electrodes and maximize the active material mass loading at a given area.^[^ ^20^, ^21^, ^22^ ^]^ Utilizing the vertical dimensions of the 3D spatial architecture electrodes can significantly increase the areal energy density of Zn‐ion batteries. Meanwhile, the 3D porous structure with a continuous conductive network and fully interconnected graded porosity is highly favorable for charge transfer between electrodes as well as at the electrolyte‐electrode interface, leading to a substantial increase in power density.^[^ ^23^, ^24^, ^25^ ^]^ Moreover, extending the contact surface to 3D space can greatly improve the designability of Zn anode structure. Further, increasing the specific surface area can effectively increase the Zn nucleation sites and reduce the local current density, thus hindering the formation of Zn dendrites.^[^ ^26^, ^27^ ^]^ Meanwhile, the micro‐structure features with 3D porous nanochannels will also lead to the uniform distribution of the electric field at the electrode/electrolyte interface, which can effectively regulate the Zn deposition/dissolution process, and significantly enhance the long‐cycle performance for Zn‐ion microbatteries.^[^ ^28^ ^]^ In addition, 3D multi‐scale structured electrodes can significantly mitigate the interfacial stress generated during charging and discharging.^[^ ^29^, ^30^ ^]^ Besides, it is equally important to optimize the 3D substrate channel size of the electrodes, because the narrow channels are not conducive to electrolyte diffusion and zinc deposition, while larger pore sizes are subject to collapse during cycling.^[^ ^31^ ^]^ Based on the above consideration, 3D printing (i.e., additive manufacturing) technology provides the possibility to accurately control the geometry of the battery device (such as size, porosity and morphology, etc.), which can accurately control the shape and structure from the micro to the macro without relying on any template, thereby improving the energy density and power density of the battery device.^[^ ^32^, ^33^, ^34^ ^]^ Meanwhile, the electrodes with orderly and low‐tortuosity by 3D printing technology are prepared to carry more active materials and form more active sites, which is conducive to the transport of electrons and ions.^[^ ^35^, ^36^ ^]^ Furthermore, the “additive” manufacturing nature of 3D printing technology is extremely cost‐effective, simplifying the preparation process and improving the controllability of electrode thickness.^[^ ^37^, ^38^ ^]^ 3D printing technology has been utilized to create high‐specific energy micro‐batteries of arbitrary shapes, which can not only provide intelligent design for the established products, maximize the optimization of product design, but also meet the special manufacturing needs in special fields.^[^ ^39^, ^40^, ^41^ ^]^ However, it is still challenging to solve the issues from both cathode and Zn anode with the 3D printing technology. Meanwhile, 3D printing technologies for battery applications face challenges of balancing printability, resolution, active material mass loading, and so on.

Herein, we report the design and fabrication of 3D‐printed quasi‐solid‐state Zn‐VOH battery devices with ultrahigh areal energy density and long‐term durability using 3D printing technology and electrodeposition process. The 3DP‐rGO/CNTs microlattices show a low‐tortuosity hierarchical porous architecture consisting of an interconnected 3D continuous carbon‐based skeleton structure with highly conductive and ultra‐lightweight characteristics. Direct growth of vertically aligned nanoarrays (VOH nanosheets and Zn nanoflakes) on the surface and interior of the 3DP‐rGO/CNTs microlattices provided a highly active interface structure with uniformly and highly dispersed active sites, which also ensured a strong covalent coupling between inorganic nanocrystals and carbon framework, resulting the electrons transfer rapidly from active materials to current collectors. Construction of ultra‐light, multi‐level pore structure thickness‐adjustable carbon‐based conductive skeleton based on 3D printing technology: i) increase the percentage of active materials for high areal energy density; ii) effectively regulate the uniform distribution of ions/electrons, to achieve uniform and fast Zn deposition/migration dynamics, and to reduce dendritic crystal formation, hydrogen precipitation reaction, and corrosion of Zn anode; iii) achieve a customizable all‐3D‐printed rechargeable aqueous Zn‐ion batteries. Specifically, the 3D printed quasi‐solid‐state Zn‐VOH batteries exhibit a high capacity of 461.3 mAh g^−1^ at 0.1 A g^−1^ and prominent cycling stability with a capacity retention of ≈85.6% after 10 000 cycles. Furthermore, four 3D printed quasi‐solid‐state Zn‐VOH batteries devices can be integrated in a single chip in series and further improved the overall output voltage, which can light up high‐power blue LED strip lights. These results indicate the promising potential of 3D printed Zn‐ion battery devices in many practical wearable applications and provide a new platform for integrated safe and wearable energy storage technologies.

## 2 Results and Discussion

**Figure 1:**
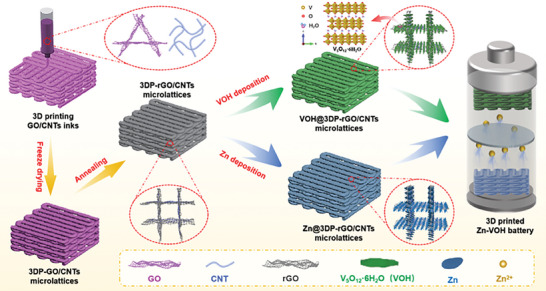
Fabrication procedures and schematics of the 3D printed Zn‐VOH battery. Schematic illustration of fabrication of VOH@3DP‐rGO/CNTs cathode and Zn@3DP‐rGO/CNTs anode.

The detailed fabrication procedures of VOH@3DP‐rGO/CNTs cathode and Zn@3DP‐rGO/CNTs anode is schematically illustrated in **Figure** **1**. First, 3DP‐GO/CNTs microlattices were constructed by an extrusion‐based 3D printing technique using a homogeneous gel inks slurry containing a mixture of graphene oxide (GO) and CNTs (Figure S1, Supporting Information). Second, 3DP‐GO/CNTs microlattices were further formed hybrid aerogels with graded pore structure by using a freeze‐drying method and followed reductive annealing under Ar atmosphere to convert GO to rGO, then 3DP‐rGO/CNTs microlattices with lightweight are successfully prepared. Finally, the interconnected V_5_O_12_·6H_2_O (VOH) nanosheet arrays and Zn nanoflake arrays directly grown on the self‐supported 3DP‐rGO/CNTs microlattices using a one‐step electrodeposition method, and further used as the dendrite‐free anode and high‐loading cathode for 3D‐printed Zn‐VOH batteries, respectively.

**Figure 2:**
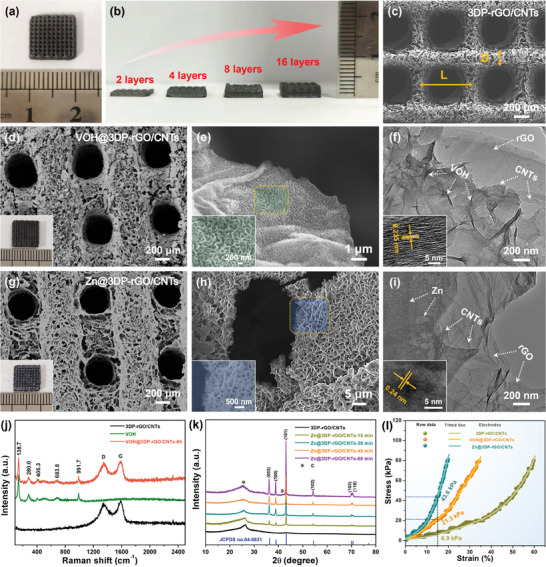
Morphology characterizations and mechanical performance of both the 3DP‐rGO/CNTs, VOH@3DP‐rGO/CNTs and Zn@3DP‐rGO/CNTs microlattices electrodes. a) Photos of 3D printed rGO/CNTs microlattice top view. b) Optical images of 3D printed rGO/CNTs microlattices electrodes with different printing layers (2–8 layers). c) SEM images of 3D printed rGO/CNTs microlattices electrodes. d,e) SEM images of the VOH@3DP‐rGO/CNTs microlattices and their gradient porous structures. f) TEM images of the synthesized VOH@3DP‐rGO/CNTs. g,h) SEM images of the Zn@3DP‐rGO/CNTs microlattices and its gradient porous structure. i) TEM images of the synthesized Zn@3DP‐rGO/CNTs. Insets in (d,g) show the top views of VOH@3DP‐rGO/CNTs and Zn@3DP‐rGO/CNTs microlattices, respectively. j) Raman pattern of the as‐prepared VOH cathode grown on 3D‐printed rGO/CNTs. k) XRD pattern of the as‐prepared Zn nanoflakes grown on 3D‐printed rGO/CNTs. l) Nominal compression stress versus nominal strain curves of these samples: 3D‐printed rGO/CNTs, VOH@3DP‐rGO/CNTs, and Zn@rGO/CNTs microlattices.

The rheological behaviors of GO hydrogels were easily controlled by adjusting the adding dose of CNTs. To further clarify the effect of CNTs, we investigated the rheological properties of pure GO dispersion and GO hydrogel ink with addition of CNTs (Figure S2, Supporting Information). The GO/CNTs inks obtained by uniformly stirring and centrifugation are printed into fine lines in layer‐by‐layer using a needle and form orthogonal multilayers with parallel porous cylindrical rods. After freeze‐drying and annealing treatments, the 3DP‐rGO/CNTs aerogel microlattices with a thickness of 2.0 mm and a thickness of 1.0 cm show ultralight features (≈20 mg cm^−3^), and can be supported by dandelion (**Figure** **2**a; Figures S3a,b and S4, Supporting Information). Meanwhile, the thickness of the 3DP‐rGO/CNTs microlattices can be adjusted by controlling the number of printing layers, as evidenced in Figure 2b. Furthermore, the homogeneous GO/CNTs gel inks also can be easily printed into various patterns with different sizes, shapes, and precisions (Figure S3c, Supporting Information), indicating the high universality and designability of 3D printing technology. As shown in Figure 2c and Figures S3d–f and S5 (Supporting Information), the self‐supported 3DP‐rGO/CNTs microlattices architecture shows the interconnected frame structure with hierarchical open holes, which is composed of randomly oriented 2D nanosheets with rough and wrinkled morphology, as well as a large number of CNTs are anchored uniformly on the surface of the rGO nanosheets to further improve the electrical conductivity and mechanical reliability of 3DP‐rGO/CNTs samples. Additionally, the 3DP‐rGO/CNTs, Zn@3DP‐rGO/CNTs and VOH@3DP‐rGO/CNTs microlattices electrodes can basically return to its original state and no serious collapse occurred after compression (Figure S6, Supporting Information), which shows that they have good mechanical properties.

After growth of VOH nanosheets by electrodeposition process (Figure S7, Supporting Information), the 3D printed microlattices architecture is well maintained, as shown in Figure 2d. The scanning electron microscopy (SEM) images in Figure 2e present that the interconnected VOH nanosheet arrays with thickness from tens to hundreds of nanometers were vertically rooted on the surface of rGO/CNTs nanosheets. The uniform deposition of VOH nanosheets indicates that the rGO/CNTs aerogel has good electrical conductivity and the 3D‐printed microlattices architecture allows efficient permeation of electrolyte. Importantly, the VOH nanosheets are not only deposited on the outer surface but also deposited into the interior of the whole 3DP‐rGO/CNTs microlattices (Figure S8, Supporting Information). Furthermore, the SEM images of the prepared VOH@3DP‐rGO/CNTs samples with different electrodeposition times are depicted in Figure S9 (Supporting Information). Notably, the thickness of VOH nanosheets layer on the 3DP‐rGO/CNTs aerogel microlattice increases slowly with the electrodeposition time from 2 to 8 h, which would achieve a high‐mass loading of VOH nanosheets from 31.3 to 111.5 mg cm^−3^ (Figure S10, Supporting Information). The transmission electron microscopy (TEM) images further validate that the polycrystalline VOH nanosheets uniformly grow on the rGO/CNTs surface (Figure 2f). According to the high‐resolution TEM image (HRTEM, inset of Figure 2f), the lattice fringes is close to 0.235 nm, which corresponds to the (005) crystal plane of V_5_O_12_·6H_2_O.^[^ ^42^ ^]^ Similarly, after growth of Zn nanoflakes by electrodeposition process, and the 3D printed microlattices architecture is also maintained well (Figure 2g). Importantly, the Zn nanoflakes are not only deposited on the outer surface but also deposited into the interior of the whole 3DP‐rGO/CNTs microlattices (Figure S11, Supporting Information). The SEM images in Figure 2h showed that Zn nanoflake arrays (≈50 nm in thickness) were uniformly anchored on rGO/CNTs nanosheets surface, which provide proper channels for electron pathways. From the HRTEM image (Figure 2i), it can be seen that the well‐defined lattice fringes with distances of 0.24 nm are attributed to the (002) plane of hexagonal Zn, which also demonstrates the highly crystalline nature of the Zn nanosheets.^[^ ^43^ ^]^ Additionally, the density of Zn nanosheet arrays can be well controlled by regulating the electrodeposition time from 15 to 60 min, and the corresponding volumetric mass loading of Zn nanosheets from 36.5 to 127.8 mg cm^−3^ (Figures S12 and S13, Supporting Information). As displayed in Figures S14 and S15 (Supporting Information), the energy‐dispersive X‐ray spectroscope (EDS) mapping results clearly illustrate that all elements are uniformly distributed on the 3DP‐rGO/CNTs aerogel microlattice surface, confirming both the VOH@3DP‐rGO/CNTs sample and the Zn@3DP‐rGO/CNTs sample were successfully prepared. The Raman spectrum of the 3DP‐rGO/CNTs, pure VOH and VOH@3DP‐rGO/CNTs samples is illustrated in Figure 2j, where the vibration peaks below 1000 cm^−1^ can be ascribed to V_5_O_12_·6H_2_O, and two prominent peaks at 1338 (D band) and 1582 cm^−1^ (G band) could be ascribed to 3DP‐rGO/CNTs, respectively.^[^ ^44^ ^]^ Figure S16 (Supporting Information) shows the X‐ray diffraction (XRD) patterns of the as‐prepared VOH@3DP‐rGO/CNTs samples with different electrodeposition time. The major diffraction peaks at ≈7.5° and ≈26.2° can be indexed to monoclinic V_5_O_12_·6H_2_O (JCPDS no. 45–1401).^[^ ^45^ ^]^ The XRD patterns of the as‐prepared Zn@3DP‐rGO/CNTs samples with different electrodeposition time were displayed in Figure 2k, the sharp diffraction peaks of all obtained samples could be well indexed to hexagonal Zn (JCPDS No. 04–0831).^[^ ^46^ ^]^ Meaningfully, the morphology of VOH nanosheet arrays and Zn nanoflake arrays deposition on carbon textiles (CTs) did not change when replacing the substrate, respectively (Figures S17–S20, Supporting Information), which demonstrates the scalability of the preparation method. The X‐ray photoelectron spectroscopy (XPS) analyses also were carried out to confirm the successful preparation of both the VOH nanosheet arrays and the Zn nanoflake arrays deposition on 3DP‐rGO/CNTs, respectively (Figures S21 and S22, Supporting Information). Moreover, the physical characteristics of both the 3DP‐rGO/CNTs and the VOH@3DP‐rGO/CNTs electrodes are evaluated by using the brunauer‐emmett‐teller (BET) and thermogravimetric (TGA) analysis (Figures S23 and S24, Supporting Information). Finally, in order to better understand the mechanical behaviors of the 3D printed electrodes, a series of experimental uniaxial compression tests of the 3DP‐rGO/CNTs, VOH@3DP‐rGO/CNTs and Zn@3DP‐rGO/CNTs microlattice samples are shown in Figure 2l. Compared to 3DP‐rGO/CNTs aerogel microlattice electrode, the compressive stress of the VOH@3DP‐rGO/CNTs and Zn@3DP‐rGO/CNTs electrodes significantly increase under the same compressive strain, indicating the grown active materials play a buffering role in compression and compression processes.

**Figure 3:**
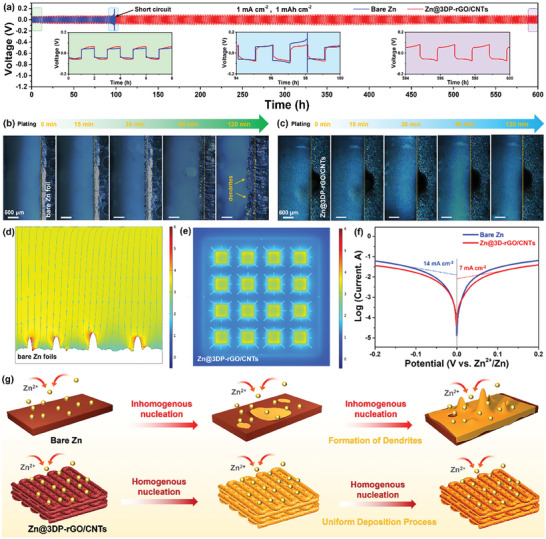
Zn deposition behavior on bare Zn foil and Zn@3DP‐rGO/CNTs. a) Galvanostatic Zn stripping/plating behavior in a Zn/Zn symmetrical cell using the aqueous 2 m ZnSO_4_ electrolyte at 1 mA cm^−2^ with an areal capacity of 1 mAh cm^−2^. In situ optical microscopy images of the Zn deposition process of (b) Bare Zn foil, c) Zn@3DP‐rGO/CNTs scaffolds at a current density of 10 mA cm^−2^. Ionic flux distribution in (d) Bare Zn foil and e) Zn@3DP‐rGO/CNTs hosts simulated by COMSOL Multiphysics. f) Corrosion curves of bare Zn foil and Zn@3DP‐rGO/CNTs anodes. g) Schematic diagram of potential dendrite growth mechanisms for bare Zn foil and Zn@3DP‐rGO/CNTs electrodes.

The superiority of 3DP‐rGO/CNTs microlattices in effectively inhibiting Zn dendrite formation is assessed by using long‐term galvanostatic plating/stripping tests on symmetric coin‐type cells assembled by two identical Zn‐based electrodes. As shown in Figure S25a,b (Supporting Information) comparing the overpotential and EIS of cells assembled by depositing zinc electrodes for different times, it was found that the cell with a deposition time of 45 min had a faster ion diffusion. **Figure** **3**a shows the voltage‐time profiles of the symmetric cells based on bare Zn foil and Zn@3DP‐rGO/CNTs electrodes at a plating/stripping capacity of 1 mAh cm^−2^ under a current density of 1 mA cm^−2^, respectively. Obviously, the Zn@3DP‐rGO/CNTs symmetrical cell delivers a highly stable voltage profile and realizes a relatively low voltage hysteresis over 600 h without irregular fluctuations, indicating that Zn@3DP‐rGO/CNTs has low polarization and fast Zn^2+^ migration kinetics. In comparison, the bare Zn symmetrical cell shows a limited cycling life and then rapidly ascending voltage polarization, suggesting that the formation of plentiful inert “dead Zn”, and the bare Zn symmetrical cell suffers from short‐circuit after cycling for only 100 h. Meanwhile, the first and last four enlarged voltage profiles based on each anode are shown in inset of Figure 3a. As can be seen, the Zn@3DP‐rGO/CNTs based symmetrical cell manifests a negligible changed voltage gap and round‐trip efficiency, indicating its favorable charge–discharge stability. In addition, the rate performances of both the Zn‐based symmetrical cells under the current density increases from 0.5 to 10 mA cm^−2^ are investigated. As shown in Figure S26 (Supporting Information), the voltage hysteresis of the Zn@3DP‐rGO/CNTs symmetrical cell are always very small with a voltage hysteresis of 65.2 mV even at 10 mA cm^−2^, which are much smaller than the bare Zn foil at the same current density. As discussed above, compared with the bare Zn foil anode, the Zn@3DP‐rGO/CNTs anode exhibits smaller voltage hysteresis, high reversibility, and prolonged cyclic stability. To further prove the above results, the Zn plating process on the surface of bare Zn foil and Zn@3DP‐rGO/CNTs electrodes is monitored in real‐time using in situ optical microscopy in a transparent electrolytic cell. Figure 3b,c shows the enlarged cross‐sectional morphologies of the top surface of bare Zn foil and Zn@3DP‐rGO/CNTs electrodes at the electrode‐electrolyte interface during the Zn plating process at different times under the current density of 10 mA cm^−2^, respectively. Consistent with other reported results, the thickness of bare Zn foil (Figure 3b) increases significantly. After 30 min of Zn deposition, rough and uneven Zn deposition with obvious lumps appears on the surface of bare Zn foil. When increasing the electroplating time to 120 min, more serious dendrite clusters on the Zn foil appear, leading to the formation of bumpy surface. During the electroplating process, visible bubbles also gradually appear on the surface of bare Zn foil, which significantly changes the electric field distribution, and thus leads to Zn dendrite formation. In contrast, a dendrite‐free morphology was observed during the whole Zn deposition process for Zn@3DP‐rGO/CNTs anode (Figure 3c). Remarkably, the thickness of the Zn@3DP‐rGO/CNTs electrode is almost unchanged, which proves the effectiveness of the 3D printed structure in guiding Zn growth and buffering volume expansion.

To further understanding the principle of 3D printed electrodes for Zn plating/stripping, the simulations on current density distribution of both the Zn‐based electrodes architecture are displayed in the liquid electrolyte. As shown in Figure 3d,e and Figure S27 (Supporting Information), the finite element simulation implemented in COMSOL Multiphysics has been applied to simulate the local current density at the interface between the electrode and the electrolyte for 2D bare Zn foil and 3D Zn@3DP‐rGO/CNTs electrodes during Zn deposition. Obviously, the current density distribution of the 2D bare Zn foil electrode is inhomogeneous, and the maximum value of the current density occurs around the tip of the 2D bare Zn foil electrode, resulting in rapid Zn deposition (Figure 3d). The charge on the tips attracts more Zn^2+^ and aggravates continuously uneven Zn deposition, leading to the Zn dendrites growth. In contrast, through the introduction of 3D printing technology, the current density within the 3D highly ordered spatial multichannel becomes much higher than that of the upper surface, as shown in Figure 3e and Figure S27 (Supporting Information). The maximum value of the current density appears on the inner surface of the Zn@3DP‐rGO/CNTs microlattice electrode, inducing a preferential homogeneous deposition of Zn into the 3D highly ordered spatial microchannels and thus easing the zinc growth on the shallow top surface.^[^ ^47^ ^]^ The 3D‐printed highly ordered porous structure helps regulate the Zn^2+^ deposition kinetics at the Zn anode interface, enhances its corrosion resistance, and further contributes to more stable Zn deposition/stripping. To further evaluate the corrosion resistance of the as‐prepared samples, Tafel linear polarization tests were performed on bare Zn foil and Zn@3DP‐rGO/CNTs electrodes. The corrosion current of the Zn@3DP‐rGO/CNTs electrode was reduced to 7 mA cm^−2^, and the bare Zn foil electrode had a corrosion current of up to 14 mA cm^−2^ (Figure 3f), indicating that the corrosion behavior was suppressed by 3D‐printed Zn electrode.^[^ ^48^ ^]^ Additionally, the schematic illustration on the underlying dendrite growth mechanism for both the bare Zn foil and the Zn@3DP‐rGO/CNTs electrodes are proposed in Figure 3g. As mentioned above, the 3D printed highly ordered porous structure provides a uniform electric field and enough Zn nucleation sites, resulting in uniform nucleation within the 3DP‐rGO/CNTs host. Moreover, the reservoir integration structure with layered multi‐channels helps to create a uniform ion field within the host body to ensure uniform Zn deposition, thus guides Zn growth along reservoir pores constructed with adjacent printing strips. Conversely, for the 2D bare Zn foil, the uneven distribution of Zn nucleus causes the uneven distribution of electric field inside the host, which further leads to the uneven deposition and growth of Zn at the dendrite site. Even worse, there is not enough room to accommodate the sharp Zn produced, which inevitably exacerbates the development of abundant dendrites and “dead Zn” growth on the surface of bare Zn, and eventually causes the battery failure.^[^ ^49^ ^]^

**Figure 4:**
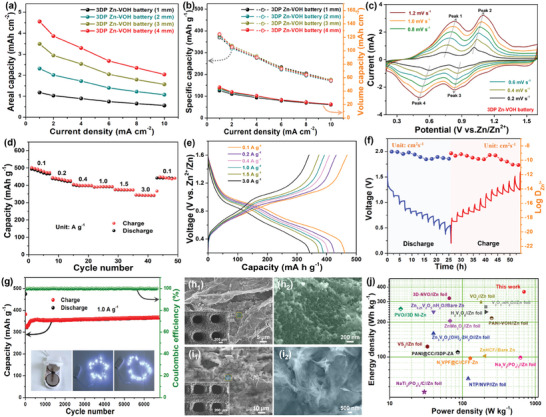
Electrochemical properties of the 3D printed Zn‐VOH button cell. Comparison of the areal capacitance (a) and the volumetric capacitance (b) of the different with various cell thicknesses, respectively. c) CV profiles from the single 3D printed Zn‐VOH button cell at various sweep rates. d,e) The corresponding discharge profiles at different current densities. f) The 3D printed button‐type battery performs 7000 cycles at 1 A g^−1^; (The white LED lamp can be powered by two button batteries connected in series). g) The discharge charging GITT curve of VOH@3DP‐rGO/CNTs cathode and the corresponding Zn^2+^ diffusion coefficient (D_Zn_). h) SEM images of the VOH@3D‐rGO/CNTs microlattices electrode after long cycle stability test. i) SEM images of the Zn@3D‐rGO/CNTs microlattices electrode after long cycle stability test. j) The electrochemical performance of the 3D‐printed Zn‐VOH button cell and the previous reported aqueous Zn‐ion battery devices.

To understand the effects of active material loading, electrode structure and thickness on the electrochemical performance of Zn‐VOH batteries, a series of comparative experiments were carried out. Figure S28a (Supporting Information) shows the cyclic voltammetry (CV) curves of VOH@3DP‐rGO/CNTs cathode (thickness: 2 mm) with different electrodeposition time at a scan rate of 0.8 mV s^−1^. Noteworthily, the enclosed area by the CV curve of the VOH@3DP‐rGO/CNTs‐6 h electrode is the maximum, indicating the highest capacity and the optimal electrode design due to its high surface area and effective porosity. By comparing the electrochemical impedance spectroscopy (EIS) of VOH‐based cathode with different electrodeposition time (Figure S28b, Supporting Information), which can be seen that VOH@3DP‐rGO/CNTs‐6 h has excellent R_ct_ value, suggesting the faster electron/proton transfer kinetics.^[^ ^50^ ^]^ For comparison, the CV and galvanostatic charge‐discharge (GCD) curves of both the VOH/CTs//Zn/CTs and the VOH@rGO/CNTs//Zn@rGO/CNTs batteries were also carried out (Figure S29, Supporting Information). Amazingly, the assembled 3D‐printed Zn‐VOH battery by VOH@3DP‐rGO/CNTs‐6 h cathode and Zn@3DP‐rGO/CNTs anode shows a much larger integral area of CV curves and longer discharge time of GCD curves than other electrodes, indicating a higher specific capacitance. Moreover, the contribution of pure 3DP‐rGO/CNTs substrate to the capacity of 3D‐printed Zn‐VOH battery is negligible capacity, as shown in Figure S30 (Supporting Information). As a word, the 3D‐printed spatial ordering electrode optimizes the transport pathways of ion and electron migration and improves the surface/interface dynamics, thus 3D‐printed Zn‐VOH batteries have excellent electrochemical performance and versatility. Additionally, as the electrode thickness increased from 1 to 4 mm, the electrochemical performance of 3D‐printed Zn‐VOH batteries was evaluated in a battery‐type Swagelok cell system. The areal capacity was improved linearly with the increment both electrodes thickness (**Figure** **4**a; Figure S31, Supporting Information), and a high reversible areal capacity of 4.52 mAh cm^−2^ at single electrode thickness of 4 mm can be achieved, suggesting that the optimized 3D‐printed electrodes have broad application prospects for aqueous rechargeable Zn‐ion batteries with high areal capacity. Meanwhile, 3D‐printed Zn‐VOH batteries exhibit similar charge/discharge curves under different electrodes thicknesses, indicating that the charge transfer and ion diffusion in 3D‐printed electrodes with highly ordered porous architecture is almost identical (Figure S32, Supporting Information). Moreover, the increase in electrodes thickness does not sacrifice the specific and volumetric capacities of 3D‐printed Zn‐VOH batteries (Figure 4b), further confirming the feasibility of the 3D‐printed electrodes for practical applications. Compared to conventional manufacturing electrode (Figure S33, Supporting Information), the 3D‐printed rGO/CNTs‐based electrodes displayed a large number of well‐interconnected hierarchical pores and constructed continuous conductive network, thus providing unobstructed channels for the rapid transport of ions from the electrolyte to the surface of active materials. Moreover, the connection between neighboring printed rods in close contact with the active electrode materials, which ensures sufficient and fast pathways for electron transfer.^[^ ^20^, ^51^ ^]^ Thus, the above two factors together contributed to the excellent electrochemical performances of the 3D‐printed Zn‐VOH batteries.

To further investigate the electrochemical kinetics of the 3D‐printed Zn‐VOH batteries, 3D‐printed Zn‐VOH button cells were assembled from the obtained VOH@3DP‐rGO/CNTs cathode (thickness: ≈2 mm) and Zn@3DP‐rGO/CNTs anode (thickness: ≈2 mm) using a 2.0 m ZnSO_4_ aqueous solution as the electrolyte and glass fiber as the separator. As shown in Figure 4c, the CV measurements of the single 3D‐printed Zn‐VO cell were obtained at different scan rates from 0.2 to 1.2 mV s^−1^. It can be observed that the two pairs of redox peaks in the CV profiles can correspond to the Zn^2+^ (de‐)intercalation processes, the reduction peak and oxidation peaks shift a little to lower and higher potentials, respectively. Meanwhile, the CV curves maintain a similar shape and the characteristic peaks broaden with increasing scan rate, indicating that a strong tolerance for Zn^2+^ insertion/extraction. Moreover, the redox pseudo‐capacitance‐like contribution in the VOH@3DP‐rGO/CNTs cathode was analyzed to explain the high‐rate performance. The capacitive response of battery chemistry can be determined according to the following relation:^[^ ^52^, ^53^ ^]^

(1) $$i=av^{b}$$

(2) logi=b×logv+loga

where *v* refers to the scan rate (mV s^−1^), *a* and *b* are adjustable parameters, and the ranges of *b* value from 0.5 to 1.0. The value of b is 0.5, indicating that the capacity is entirely due to the diffusion‐controlled process, while a value of 1.0 for b indicates a fully capacitive process. As shown in Figure S34a (Supporting Information), the b‐value of 0.89, 0.69, 0.80, and 0.70 calculate from the redox peaks, indicating that the combination of ion diffusion and capacitive behavior synergistically controls the Zn^2+^ storage process. Thus, the fast Zn^2+^ diffusion dynamic behavior and moderate pseudocapacitance behavior make the 3D‐printed Zn‐VOH batteries have excellent rate performance. In addition, the capacity can be split into capacitive (*k*_1_ *v*) and diffusion‐controlled (*k*_2_ *v* ^1/2^) processes at a certain scan rate, within a specific voltage range during cycling, as follows:^[^ ^54^, ^55^ ^]^

(3) $$i=k_{1}v+k_{2}v$$

(4) $$\frac{i}{v^{1/2}}=k_{1}v^{1/2}+k_{2}$$

According to the above relation, the shaded area represents the capacitive contribution to the total capacity at a scan rate of 0.6 mV s^−1^, which contributes 81.6% of the total capacity (Figure S34b, Supporting Information), reflecting impressive rate capability. Furthermore, the bar plot of capacitive and diffusive controlled contribution at different scan rates is illustrated in Figure S34c (Supporting Information). The results show that the capacitance contribution increases from 72.7% to 85.2% when the scan rate is increased from 0.2 to 1.2 mV s^−1^, which contributes to remarkable rate capability of 3D‐printed Zn‐VOH batteries. Moreover, the rate performance of 3D‐sprinted Zn‐VOH batteries over a current range from 0.1 to 3.0 A g^−1^ is shown in Figure 4d. The 3D‐printed Zn‐VOH cell delivers reversible capacities of 461, 432, 405, 389, 374, and 343 mAh g^−1^ at the current densities of 0.1, 0.2, 0.4, 1.0, 1.5, and 3.0 A g^−1^, respectively. When the rate returns to 0.1 A g^−1^, the reversible capacity can recover to ≈440 mAh g^−1^, indicating outstanding structure stability of both the 3DP‐rGO/CNTs‐based microlattice electrodes. The corresponding charge/discharge curves at the selected current rate appear a similar shape with small polarization (Figure 4e), indicating that excellent charge diverting dynamics for efficient electrochemical procedures. To investigate the electrochemical kinetics of Zn^2+^ removal/insertion during the charge/discharge process, the apparent Zn^2+^ diffusion coefficient (D_Zn_) in the VOH@3DP‐rGO/CNTs cathode was revealed by the galvanostatic intermittent titration technique (GITT).^[^ ^56^ ^]^ Accordingly, the D_Zn_ of the VOH@3DP‐rGO/CNTs electrodes calculated by GITT curves are to be 10^−8^ to 10^−11^ cm^2^ s^−1^ (Figure 4f). To further evaluate the long‐cycle performance of 3D printed Zn‐VOH cells, a constant current charge/discharge testis are performed at a high current density of 1.0 A g^−1^ (Figure 4g). A capacity retention rate of ≈97% relative to the highest achievable capacity (≈368 mAh g^−1^) was obtained after 100 cycles, and still remained ≈94% even after ≈7000 cycles. It is worth noting that the coulomb efficiency (CE) is close to 100%, further demonstrating high reversibility. Meanwhile, two 3D‐printed Zn‐VOH button cells connected in series can easily light the LED strip light (inset of Figure 4g). Moreover, the SEM images of the VOH@3DP‐rGO/CNTs cathode and Zn@3DP‐rGO/CNTs anode after long cycle testing showed that the highly ordered porous structure and morphology of the 3D‐printed electrodes are still well maintained (Figure 4h,i), further indicating the stability and excellent cycling behavior of the 3D‐printed electrode materials. Noteworthily, the 3D‐printed Zn‐VOH batteries exhibits competitive gravimetric energy/power densities (364.5 Wh kg^−1^ at 700 W kg^−1^, based on the active materials mass of the whole device), which are much better than those of the previously reported aqueous Zn‐ion batteries (Figure 4j; Table S1, Supporting Information).

**Figure 5:**
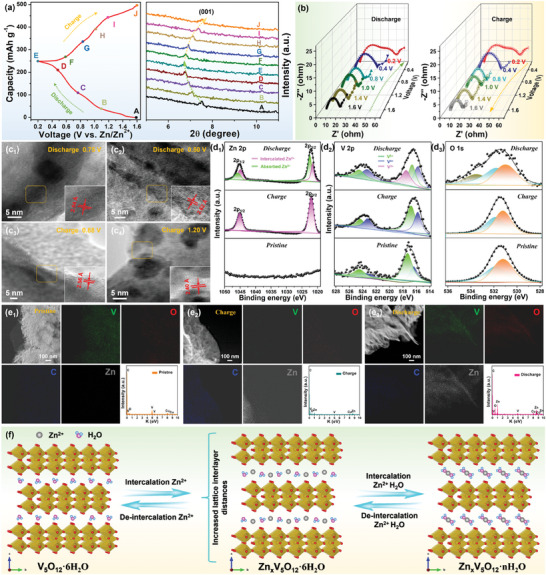
The electrochemical reaction mechanism of the VOH@3DP‐rGO/CNTs cathode. a) Ex situ XRD patterns of Bragg peak around (001) of VOH during the cycle and the corresponding charge/discharge curves at 0.2 A g^−1^. b) In situ EIS measures patterns in the initial charge–discharge cycle. c_1_–c_4_) HRTEM images of VOH at Discharge‐0.75/0.50 V, Charge‐0.88/1.20 V states, respectively. d_1_–d_3_) XPS spectra of Zn 2p, V 2p, and O 1s regions at the pristine, 1st charged (1.6 V), and 1st discharged (0.2 V) states. e_1_–e_3_) STEM images of the pristine, 1st charged, and 1st discharged electrodes along with the relevant elemental mapping images. f) Schematic illustration of the phase transition from VOH to Zn_x_VOH and the reaction mechanism on Zn_x_VOH electrode.

**Figure 6:**
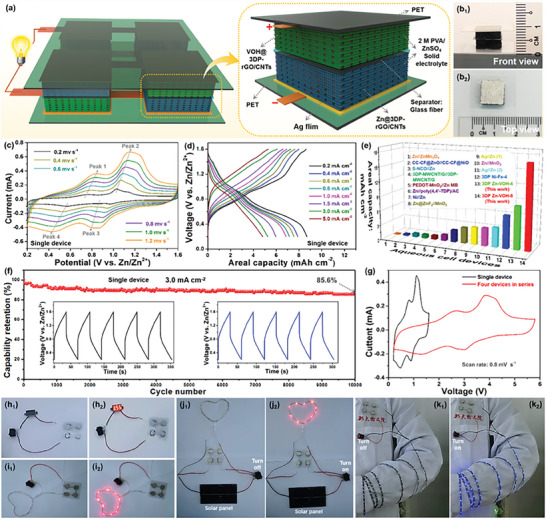
Electrochemical properties of the 3D printed quasi‐solid‐state Zn‐VOH battery devices. a) Schematic diagram of 3D printed quasi‐solid‐state Zn‐VOH battery device based on VOH@3DP‐rGO/CNTs cathode and Zn@3DP‐rGO/CNTs anode. c) The CV curves of the 3D printed quasi‐solid‐state Zn‐VOH battery devices at different scan rates. d) The galvanostatic charge/discharge curves of the 3D printed quasi‐solid‐state Zn‐VOH battery device at different scan rates. e) Areal capacity comparison of the 3D printed quasi‐solid‐state Zn‐VOH battery devices with previous studies. f) Cycle characteristics of 3D printed quasi‐solid‐state Zn‐VOH battery device with gel electrolyte for 10 000 cycles at 3 mA cm^−2^. g) The CV curves of four 3D printed quasi‐solid‐state Zn‐VOH battery devices group and a single 3D printed quasi‐solid‐state Zn‐VOH battery device at the scan rate of 1 mV s^−1^. h–j) The red LED strip lamp is powered by four 3D‐printed quasi‐solid‐state Zn‐VOH battery devices connected in series and can be powered by a solar panel when there is no power. k) The blue LED strip lights wrapped around the human arm is powered by four 3D‐printed quasi‐solid‐state Zn‐VOH battery devices in series.

To deeply investigate the Zn^2+^ (de‐)intercalation reaction mechanism process, a comprehensive study was carried out using a combination of in situ EIS and ex situ XRD, TEM, XPS analyses. As displayed in **Figure** **5**a, the first discharge/charge profile of the VOH@3DP‐rGO/CNTs cathode (marked points A‐J) was selected for ex situ XRD patterns. When the electrode was discharged to 0.2 V (A→E), the (001) lattice plane diffraction peak of VOH gradually changed from 7.5° to 6.7° with the insertion of Zn^2+^. These results showed that the original VOH crystal gradually evolved into a layered Zn_x_VOH phase after complete discharge, and the (001) facet spacing increased from 2.35 to 2.65 Å. The subsequent charging process (E→J) caused the crystal to evolve in the opposite direction during charging, which was related to the Zn^2+^ deintercalation. To investigate the charge transfer kinetics of VOH@3DP‐rGO/CNTs cathode at various charge/discharge stages, in situ EIS was also employed (Figure 5b). The Nyquist plots at different voltages consist of a concave semicircle at high‐frequency region and a sloping line at low‐frequency region, which are described as the charge‐transfer resistance (R_ct_) and the Warburg impedance (Z_w_), respectively.^[^ ^57^ ^]^ The intercept at the real axis reflects the solution resistance (R_s_), which is related with the ionic conductivity in the electrolyte. Figure S35 (Supporting Information) demonstrates the calculated values of R_ct_ and R_s_ under different discharge/charge states. The R_s_ is very low (<2 Ω) and remains constant, indicating that possess high electronic conductivity and fast ion diffusion in all discharge/charge states. Interestingly, R_ct_ increases significantly from 13.4 to 46.1 Ω when the fully charged state to the fully discharged state, and the change trend of R_ct_ is fully reversible during the subsequent charging process. In addition, the ex‐HRTEM images further indicate that the intercalation of Zn^2+^ into the sample and the detachment from the sample are accompanied by changes in the interlayer d‐spacing of VOH (Figure 5c_1_–c_4_).^[^ ^58^ ^]^ Compared with original VOH, the interlayer d‐spacing slightly increases from 2.35 to 2.54 Å in the Discharge‐0.75 V‐state and 2.62 Å in the Discharge‐0.50 V state, which is caused by the insertion of Zn^2+^/H^+^. Meanwhile, the interlayer d‐spacing returned to 2.35 Å when charging to Charge‐1.60 V state. The XPS analyses were performed to further explore the essence of the excellent electrochemical behaviors of the VOH@3DP‐rGO/CNTs cathode materials. Figure 5d_1_ displays the Zn 2p spectra of VOH@3DP‐rGO/CNTs cathode at pristine, fully charged, and fully discharged states in the first cycle. In the pristine state, no signal for Zn element was detected. On the fully charged VOH@3DP‐rGO/CNTs cathode, only one Zn 2p_3/2_ component (1021.9 eV) was displayed due to the remaining surface absorption. In the fully discharged state, the electrode shows two Zn 2p_3/2_ components located at 1022.5 and 1021.6 eV.^[^ ^59^ ^]^ The signal at 1022.5 eV accurately declaring the insertion of Zn^2+^ ions into VOH nanosheets, and the signal at 1021.6 eV can be ascribed to the Zn^2+^ salts absorbed on the electrode surface.^[^ ^60^ ^]^ As shown in Figure 5d_2_, the evolution of the V 2p region of the XPS spectra was recorded during battery operation. The V^5+^ component (V 2p_3/2_: 517.3 eV) and the V^4+^ signal (V 2p_3/2_: 516.5 eV) were observed in the pristine state.^[^ ^59^, ^61^ ^]^ In the fully discharged state, the V^4+^ signal is strongly enhanced, and the component of V^3+^ clearly appears clearly due to the insertion of Zn^2+^ ions. When the VOH@3DP‐rGO/CNTs cathode was charged to 1.6 V, the pristine V 2p spectrum re‐emerged. Moreover, the O 1s XPS region in the pristine electrode can be fitted into two peaks (Figure 5d_3_), which are related to the O 1s of the VO_x_ layer (531 eV) and the water molecules (532 eV) in the VOH interlayer, respectively. During the discharge state, a new broad peak appears at a higher binding energy of 534 eV, which can be assigned to the interactions between inserted Zn^2+^ and the oxide layer.^[^ ^62^ ^]^ Finally, the scanning transmission electron microscopy (STEM) and the corresponding elemental mapping images of the pristine and charge/discharge electrodes are presented in Figure 5e_1_–e_3_. Under the charging state, a trace amount of Zn is detected, which is attributed to the small amount of adsorbed Zn^2+^ salt still remaining on the electrode surface. However, the uniform dispersion of Zn, V, and O confirmed the Zn^2+^ insertion reaction in the discharging state. Based on the above analysis, the reaction mechanism suggests that the layered VOH nanosheets permit reversible Zn^2+^ insertion/extraction and accompanied by reversible evolution of V and O chemical states. The Zn storing mechanism in VOH active material is illustrated in Figure 5f, which illustrates the phase transition from VOH to ZVOH and the embedding mechanism of Zn^2+^ in VOH, the corresponding electrochemical reaction taking place in cathode can be formulated as follows: V_5_O_12_·6H_2_O + xZn^2+^ + 2xe^−^ ↔ Zn_x_V_5_O_12_·6H_2_O, where x is the number of the intercalated Zn^2+^ cations. Then the inserted Zn^2+^ and H_2_O of crystallization in VOH combine to synthesize [ZnH_2_O]^2+^, and the corresponding Zn_x_V_5_O_12_·6H_2_O is transformed into Zn_x_V_5_O_12_·nH_2_O (**Figure** **6**).^[^ ^8^, ^63^ ^]^

In order to verify the practicability of the 3D printed Zn‐VOH batteries for wearable/portable devices, an in‐depth study of the 3D‐printed quasi‐solid‐state Zn‐VOH battery devices with the integrated configuration was conducted, and the schematic illustration is shown in Figure 6a. Conductive Ag adhesive layers were served as current collectors on flexible poly(ethylene terephthalate) (PET) substrate, 2 m PVA/ZnSO_4_ hydrogel electrolyte was used as the quasi‐solid‐state electrolyte, and glass fiber films were used as separator. In the 3D‐printed quasi‐solid‐state Zn‐VOH battery device, both the 3D‐printed positive and negative electrodes coated with solid electrolyte maintains a porous structure. The side and top views of the 3D‐printed quasi‐solid‐state Zn‐VOH battery device were shown in Figure 6b_1_,b_2_, respectively. Similarly, as shown in Figure 6c, the representative CV curves of the the 3D‐printed quasi‐solid‐state Zn‐VOH battery device at different scanning rates from 0.2 to 1.2 mV s^−1^. Two pairs of the redox peaks showed in the CV curves are consistent with the voltage platforms during the discharge process shown in Figure 6d and Figure S36 (Supporting Information), corresponding to the Zn^2+^ (de)intercalation processes. Specifically, the 3D printed quasi‐solid‐state Zn‐VOH battery device with 4 mm (3DP Zn‐VOH‐4) thickness endows a high areal capacity of ≈8.63 mAh cm^−2^ at 0.2 mA cm^−2^, even the areal capacity can reach ≈17.06 mAh cm^−2^ when the device thickness is 8 mm (3DP Zn‐VOH‐8), which are significantly higher than those of earlier reported rechargeable aqueous Zn‐ion batteries (Figure 6e). As shown in Figure 6f, the capacitance retention rate is nearly 85.6% after 10 000 cycles at 3.0 mA cm^−2^, showing that the 3D‐printed quasi‐solid‐state Zn‐VOH battery device has outstanding electrochemical stability. The CV curves of four 3D‐printed quasi‐solid‐state Zn‐VOH battery devices in series (Figure 6g) show the voltage range extends from 1.4 to 5.6 V, which are consistent with the discharge/charge curves (Figure S37, Supporting Information). Generally, the energy stored in a single 3D‐printed quasi‐solid‐state Zn‐VOH battery device is too low to meet the needs of practical applications. Therefore, several 3D‐printed quasi‐solid‐state Zn‐VOH battery devices can be connected together in series, parallel or series‐parallel combinations to generate higher voltage or current. Here, we combine four 3D‐printed quasi‐solid‐state Zn‐VOH battery devices in series into a unit by designing conductive Ag adhesive layers patterns on PET substrate (Figure 6h,i). The obtained integrated 3D‐printed quasi‐solid‐state Zn‐VOH battery device unit can easily light up the red LED light band (3.56 V), which can be powered by a solar panel when there is no power (Figure 6j). In addition, the blue LED strip lamp wrapped around the human arm is powered by four 3D‐printed quasi‐solid‐state Zn‐VOH battery devices in series (Figure 6k), which shows the good practical application prospect of the 3D‐ printed quasi‐solid‐state Zn‐VOH battery.

To further demonstrate the application of its wearable technology, both the mechanical flexibility and the thermal stability performance of the flexible 3D‐printed quasi‐solid‐state Zn‐VOH battery devices were evaluated. As shown in Figure S38 (Supporting Information), the bending and twisting performance of the 3D‐printed microlattice electrodes on a flexible PET substrate were measured, and it can be found that the electrode sheet can still maintain a stable structure with the large‐angle bending and torsion test strands. Based on 2.0 m PVA/ZnSO_4_ gel electrolyte, the flexible 3D‐printed quasi‐solid‐state Zn‐VOH battery device was assembled by separating the VOH@3DP‐rGO/CNTs and Zn@3DP‐rGO/CNTs microlattices electrodes with a piece of glass fiber membrane as the separator, as shown in Figure S39a–c (Supporting Information). While different applied strains were applied in the the single flexible 3D‐printed quasi‐solid‐state Zn‐VOH battery device, the CV curves showed only slight deviation (Figure S39d, Supporting Information), indicating that the exceptional flexibility and the electrochemical stability of the flexible 3D‐printed quasi‐solid‐state Zn‐VOH battery devices under different bending angles from 0° to 135°, which is also confirmed by their charge/discharge curves (Figure S39g, Supporting Information). Meanwhile, the capacity of the single flexible 3D‐printed quasi‐solid‐state Zn‐VOH battery device is only slight decrease after 100 bending‐releasing cycles, as shown in Figure S39e,h (Supporting Information), which indicates that the excellent long‐term durability of the 3D‐printed quasi‐solid‐state Zn‐VOH battery device under continuous flexing and exposure to body sweat or environmental moisture. Additionally, to assess the thermal stability of the batteries, the corresponding electrochemical performance and application in scenarios of overcharging, mechanical damage or extreme temperatures (≈60 °C) are also demonstrated (Figures S39f,i and S40, Supporting Information). These results can be further proved that the 3D‐printed quasi‐solid‐state Zn‐VOH battery devices have the potential to fulfill the wearable devices.

## 3 Conclusion

In summary, a wearable quasi‐solid‐state aqueous Zn‐VOH battery has been developed by using VOH nanosheet arrays and Zn nanoflake arrays homogeneously electrodeposited on 3D‐printed rGO/CNTs hybrid aerogel microlattices as the cathode and anode, respectively. For the VOH@3DP‐rGO/CNTs cathode, the layered VOH nanosheet arrays enables highly reversible and ultra‐fast Zn^2+^ insertion/extraction processes, and the structural water effectively shields the electrostatic interactions between Zn^2+^ cations and the host anions, resulting in good kinetics and great stability. The constructed Zn@3DP‐rGO/CNTs anode with highly ordered spatial hierarchical porous structure and super‐hydrophilic surface can effectively improve the electric‐field distribution, inducing uniform Zn deposition without the growth of Zn dendrites, which are confirmed by simulating the current density distribution of the Zn‐based electrodes in electrolyte and observing the Zn plating/stripping process using in situ optical microscopy. Moreover, the 3DP‐rGO/CNTs‐based microlattice electrodes present abundant open and hierarchical porous structure, which enables pathways for ion/electrolyte transportation and accommodates volume change upon cycling. Consequently, the 3D‐printed Zn‐VOH batteries with excellent long‐term stability and high capacity are achieved by matching VOH@3DP‐rGO/CNTs cathode with Zn@3DP‐rGO/CNTs anode, which exhibits a reversible capacity of ≈461.3 mAh g^−1^ at 0.1 A g^−1^, high energy and power density (364.5 Wh kg^−1^ at 700 W kg^−1^), and long cycle life (reversible capacity sustains ≈364.5 mAh g^−1^ after near 7000 cycles at 1.0 A g^−1^). On the basis of the PVA/ZnSO_4_ gel electrolyte, an ultrahigh areal capacity of ≈17.06 mAh cm^−2^ at 0.2 mA cm^−2^ can be achieved in single 3D‐printed quasi‐solid‐state Zn‐VOH battery device with 8 mm thickness, which retaining capacity up to ≈85.6% after 10 000 cycles at 3.0 mA cm^−2^. Additionally, several 3D‐printed quasi‐solid‐state Zn‐VOH battery devices can be integrated in series on a flexible substrate to increase the overall output voltage, which can light up a high‐power LED strip lights. This 3D printed Zn‐VOH batteries system is expected to be used in flexible electronic devices and large‐scale energy storage applications due to its excellent electrochemical performance, high safety, ease of assembly and high flexibility.

## 4 Experimental Section

### Preparation of 3D Printed rGO/CNTs Microlattices

The prepared 3D printing inks were filled into a syringe (5 mL), and further fixed to a modified DIW 3D printer (MUSASHI, SM200SX‐3A). The specially designed 3D network microlattice pattern was printed using a disposable micro‐nozzle with an inner diameter of 100 µm. During the printing process, the pressure was set between 0.15 and 0.25 MPa, and the moving speed of the syringe was 2.0 mm s^−1^. The printed patterns on the glass substrate were immersed into liquid nitrogen for 5 min, and then freeze‐dried at −40 °C for 24 h to remove excess water and further form porous aerogels structure. Finally, the 3DP‐GO/CNTs microlattices were annealed in a tube‐type furnace at 650 °C for 3 h under Ar gas flow to form 3DP‐rGO/CNTs microlattices, and the heating rate was set to 2.0 °C min^−1^.

### Synthesis of VOH@3DP‐rGO/CNTs Cathode

Interconnected V_5_O_12_·6H_2_O (VOH) nanosheet arrays were in situ grown on the 3DP‐rGO/CNTs microlattices via a simple and extensible electrodeposition technology. In a typical process, the electrodeposition experiment was carried out in a 100 mL electrolytic cell with a two‐electrode system. The 3DP‐rGO/CNTs microlattices sample was used as working electrode, platinum foil was used as both reference and counter electrode, and 0.1 m VOSO_4_ aqueous solution without any additives was used as electrodeposition electrolyte. Electrodeposition process was prepared at a constant potential of 2.6 V for different time from 2 to 8 h at 50 °C using the VM3 electrochemical workstation (Bio‐Logic Inc.). The possible electrodeposition reactions on the working electrode is as follows: 5VO^2+^ + 13H_2_O → V_5_O_12_·6H_2_O + 4e^−^ + 14H^+^. After that, the as‐obtained products were washed several times with deionized (DI) water and ethanol, and then dried at 40 °C under vacuum overnight. For the 2 mm thickness 3DP‐rGO/CNTs microlattices (1.0 × 1.0 × 0.2 cm^3^, ≈20 mg cm^−3^) electrodes, the electrodeposition time was varied from 2 to 8 h to control the mass loading of VOH nanosheet arrays from 31 to 112 mg cm^−3^. The as‐prepared VOH@3DP‐rGO/CNTs samples could be directly used as an binder‐free and self‐supporting electrodes without additional treatment.

### Synthesis of Zn@3DP‐rGO/CNTs Anode

Ultrathin Zn nanoflake arrays were vertically grown on the 3DP‐rGO/CNTs microlattices aerogel by a conventional electrodeposition method. In a typical procedure, the 3DP‐rGO/CNTs microlattices was used as the working electrode, and Pt foil was used as reference and counter electrode, an electrolyte solution with 6.0 g ZnSO_4_·7H_2_O, 6.0 g Na_2_SO_4_, and 1.0 g H_3_BO_3_ dissolved in 40 mL DI water, and a constant current of 10 mA cm^−2^ for different time (such as 15, 30, 45, and 60 min). And then the sample was taken out, cleaned with DI water and ethanol, and dried in a vacuum oven at 70 °C overnight, the area mass‐loadings of the Zn nanoflakes was from 36 to 127 mg cm^−3^.

### Fabrication of the 3D Printed Zn‐VOH Battery

Rechargeable aqueous Zn‐VOH battery could be safely prepared in ambient air. A CR2032 coin‐type cell was fabricated using the VOH@3DP‐rGO/CNTs cathode, Zn@3DP‐rGO/CNTs anode, glass fiber membrane (Whatman, GF/C) separator, and 2.0 m ZnSO_4_ aqueous electrolyte. The 3D‐printed quasi‐solid‐state aqueous Zn‐VOH battery device was assembled using the VOH@3DP‐rGO/CNTs microlattices as the cathode and the Zn@3DP‐rGO/CNTs microlattices as the anode, with a piece of glass fiber membrane as the separator and 2.0 m PVA/ZnSO_4_ gel polymer as the electrolyte, and then packing with commercial PET films. First, the gel electrolyte was obtained using 3.0 g of poly vinyl alcohol (PVA) dissolved into 20 mL of H_2_O and then mixed with 20 mL of 2.0 m ZnSO_4_ solution at 90 °C with strongly stirring. Next, both the 3D‐printed cathode and anode were immersed into the gel electrolyte, and then removed after 5 min. After that, they were dried in an indoor environment for 30 min, and then assembled face‐to‐face with a piece of glass fiber soaked in gel electrolyte as a diaphragm. Until the electrolyte solidified, the assembled 3D‐printed quasi‐solid‐state aqueous Zn‐VOH battery device with lightweight and mechanical robustness was then packed and further tested. The actual area of the obtained 3D‐printed quasi‐solid‐state aqueous Zn‐VOH battery device was ≈1.0 cm^−2^.

### Materials Characterization

The morphologies and structures of the as‐prepared samples were characterized using field‐emission scanning electron microscopy (FESEM, JSM‐7600F, JEOL, Japan) and transmission electron microscopy (TEM, JEM‐2100F, JEOL, Japan). The chemical characterization/elemental analysis of the as‐obtained samples were performed by energy‐dispersive X‐ray spectroscope (EDS). The crystal structures and crystallography of the as‐synthesized samples were identified using X‐ray diffraction (XRD, Bruker AXS D8 Focus, Germany) techniques. Raman analysis of the as‐obtained samples was performed using the confocal Raman system (WITec Instruments Corp., Ulm, Germany) equipped with a 532 nm excitation laser. The specific surface areas and derived pore size distribution of the as‐prepared samples were calculated using the Brunauer–Emmett–Teller (BET) method. The rheological properties of the 3D‐printing inks were measured at 25 °C by a rotational rheometer (Discovery HR‐3, TA Instruments, U.S.A.). The compression stress–strain characteristics were performed using a universal testing machine (WDW‐5G, Jinan Hengsi Shanda Co., Ltd, Jinan, China) at room temperature with a tensile rate of 0.1 mm min^−1^. The weight of 3D‐printed samples was measured using a semi‐micro analytical balance (GR‐202, A&D, Japan).

### Electrochemical Measurements

The galvanostatic charge–discharge measurements were conducted using a multi‐channel battery testing system (Neware, Shenzhen, China) in the voltage range 0.2–1.6 V (versus Zn^2+^/Zn) at various current densities ranging from 0.1 to 3.0 A g^−1^ (or 0.2 to 5.0 mA cm^−2^). The specific capacity was calculated based on the mass of the whole 3D‐printed cathode. Cyclic voltammetry curves were recorded at different scan rates (0.2–1.2 mV s^−1^) within a voltage window of 0.2–1.6 V. Electrochemical impedance spectra (EIS) measurements were carried out in the frequency range of 100 kHz–10 mHz at open circuit voltage and AC perturbation of 5 mV. Both CV and EIS were conducted using a VMP3 electrochemical workstation (VMP3, Bio Logic, France).

## Conflict of Interest

The authors declare no conflict of interest.

## Supporting information

Supporting Information

## Data Availability

The data that support the findings of this study are available from the corresponding author upon reasonable request.

## References

[1] B. Dunn , H. Kamath , J.‐M. Tarascon , Science 2011, 334, 928. 22096188 10.1126/science.1212741

[2] D. Chao , W. Zhou , F. Xie , C. Ye , H. Li , M. Jaroniec , S.‐Z. Qiao , Sci. Adv. 2020, 6, eaba4098. 32494749 10.1126/sciadv.aba4098 PMC7244306

[3] F. Mo , G. Liang , Z. Huang , H. Li , D. Wang , C. Zhi , Adv. Mater. 2020, 32, 1902151. 10.1002/adma.201902151 31364216

[4] X. Li , X. Zhang , J. Xu , Z. Duan , Y. Xu , X. Zhang , L. Zhang , Y. Wang , P. K. Chu , Adv. Sci. 2024, 11, 2305467. 10.1002/advs.202305467 PMC10837388 38059813

[5] Y. Wang , Q. Li , H. Hong , S. Yang , R. Zhang , X. Wang , X. Jin , B. Xiong , S. Bai , C. Zhi , Nat. Commun. 2023, 14, 3890. 37393327 10.1038/s41467-023-39634-8 PMC10314915

[6] D. Pan , H. Yang , Y. Liu , H. Wang , T. Xu , D. Kong , J. Yao , Y. Shi , X. Li , H. Y. Yang , Y. Wang , Nanoscale 2023, 15, 17482. 37861463 10.1039/d3nr03046f

[7] Z. Lyu , G. J. H. Lim , J. J. Koh , Y. Li , Y. Ma , J. Ding , J. Wang , Z. Hu , J. Wang , W. Chen , Y. Chen , Joule 2021, 5, 89.

[8] G. Li , L. Sun , S. Zhang , C. Zhang , H. Jin , K. Davey , G. Liang , S. Liu , J. Mao , Z. Guo , Adv. Funct. Mater. 2024, 34, 2301291.

[9] X. Jia , C. Liu , Z. G. Neale , J. Yang , G. Cao , Chem. Rev. 2020, 120, 7795. 32786670 10.1021/acs.chemrev.9b00628

[10] C. Zhong , B. Liu , J. Ding , X. Liu , Y. Zhong , Y. Li , C. Sun , X. Han , Y. Deng , N. Zhao , W. Hu , Nat. Energy 2020, 5, 440.

[11] M. Zhao , J. Rong , F. Huo , Y. Lv , B. Yue , Y. Xiao , Y. Chen , G. Hou , J. Qiu , S. Chen , Adv. Mater. 2022, 34, 2203153. 10.1002/adma.202203153 35635220

[12] Y. Wang , S. Wei , Z.‐H. Qi , S. Chen , K. Zhu , H. Ding , Y. Cao , Q. Zhou , C. Wang , P. Zhang , X. Guo , X. Yang , X. Wu , L. Song , Proc. Natl. Acad. Sci. USA 2023, 120, e2217208120. 36940337 10.1073/pnas.2217208120 PMC10068788

[13] J. Ma , S. Zheng , L. Chi , Y. Liu , Y. Zhang , K. Wang , Z.‐S. Wu , Adv. Mater. 2022, 34, 2205569. 10.1002/adma.202205569 35952711

[14] Y. Kuang , C. Chen , D. Kirsch , L. Hu , Adv. Energy Mater. 2019, 9, 1901457.

[15] J. Yan , S. Huang , Y. V. Lim , T. Xu , D. Kong , X. Li , H. Y. Yang , Y. Wang , Mater. Today 2022, 54, 110.

[16] B. Yong , D. Ma , Y. Wang , H. Mi , C. He , P. Zhang , Adv. Energy Mater. 2020, 10, 2002354.

[17] D. Lin , S. Chandrasekaran , J. B. Forien , X. Z. Xue , A. Pinongcos , E. Coester , M. A. Worsley , Y. T. Li , Adv. Energy Mater. 2023, 13, 2300408.

[18] K. Yang , Y. Hu , T. Zhang , B. Wang , J. Qin , N. Li , Z. Zhao , J. Zhao , D. Chao , Adv. Energy Mater. 2022, 12, 2202671.

[19] T. Huang , K. Xu , N. Jia , L. Yang , H. Liu , J. Zhu , Q. Yan , Adv. Mater. 2023, 35, 2205206. 10.1002/adma.202205206 36453716

[20] B. Yao , S. Chandrasekaran , J. Zhang , W. Xiao , F. Qian , C. Zhu , E. B. Duoss , C. M. Spadaccini , M. A. Worsley , Y. Li , Joule 2019, 3, 459.

[21] D. Kong , Y. Wang , S. Huang , B. Zhang , Y. V. Lim , G. J. Sim , P. V. y Alvarado , Q. Ge , H. Y. Yang , ACS Nano 2020, 14, 9675. 32628008 10.1021/acsnano.0c01157

[22] X. Xue , D. Lin , Y. Li , Small Struct. 2022, 3, 2200159.

[23] X. Li , S. Ling , L. Zeng , H. He , X. Liu , C. Zhang , Adv. Energy Mater. 2022, 12, 2200233.

[24] C. Kim , B. Y. Ahn , T.‐S. Wei , Y. Jo , S. Jeong , Y. Choi , I.‐D. Kim , J. A. Lewis , ACS Nano 2018, 12, 11838. 30395434 10.1021/acsnano.8b02744

[25] Y. Kuang , C. Chen , D. Kirsch , L. Hu , Adv. Energy Mater. 2019, 9, 1901457.

[26] J. Yang , B. Yin , Y. Sun , H. Pan , W. Sun , B. Jia , S. Zhang , T. Ma , Nano‐Micro Lett. 2022, 14, 42. 10.1007/s40820-021-00782-5 PMC8724388 34981202

[27] F. Li , D. Ma , K. Ouyang , M. Yang , J. Qiu , J. Feng , Y. Wang , H. Mi , S. Sun , L. Sun , C. He , P. Zhang , Adv. Energy Mater. 2023, 13, 2204365.

[28] Y. Mu , Z. Li , B. Wu , H. Huang , F. Wu , Y. Chu , L. Zou , M. Yang , J. He , L. Ye , M. Han , T. Zhao , L. Zeng , Nat. Commun. 2023, 14, 4205. 37452017 10.1038/s41467-023-39947-8 PMC10349079

[29] C. Li , X. Xie , H. Liu , P. Wang , C. Deng , B. Lu , J. Zhou , S. Liang , Natl. Sci. Rev. 2022, 9, nwab177. 35265341 10.1093/nsr/nwab177 PMC8900688

[30] B. Tang , L. Shan , S. Liang , J. Zhou , Energy Environ. Sci. 2019, 12, 3288.

[31] G. Shi , X. Peng , J. Zeng , L. Zhong , Y. Sun , W. Yang , Y. L. Zhong , Y. Zhu , R. Zou , S. Admassie , Z. Liu , C. Liu , E. I. Iwuoha , J. Lu , Adv. Mater. 2023, 35, 2300109. 10.1002/adma.202300109 37009654

[32] Y. Zhu , J. Qin , G. Shi , C. Sun , M. Ingram , S. Qian , J. Lu , S. Zhang , Y. L. Zhong , Carbon Energy 2022, 4, 1242.

[33] E. MacDonald , R. Wicker , Science 2016, 353, aaf2093. 27708075 10.1126/science.aaf2093

[34] Z. Wang , Z. Huang , H. Wang , W. Li , B. Wang , J. Xu , T. Xu , J. Zang , D. Kong , X. Li , H. Y. Yang , Y. Wang , ACS Nano 2022, 16, 9105. 35666854 10.1021/acsnano.2c01186

[35] M. Sha , H. Zhao , Y. Lei , Adv. Mater. 2021, 33, 2103304. 34561923 10.1002/adma.202103304 PMC11468247

[36] Y. Liu , H. Wang , H. Yang , Z. Wang , Z. Huang , D. Pan , Z. Zhang , Z. Duan , T. Xu , D. Kong , X. Li , Y. Wang , J. Sun , ACS Nano 2023, 17, 10844. 37204014 10.1021/acsnano.3c02506

[37] B. Li , M. Yu , Z. Li , C. Yu , H. Wang , Q. Li , Adv. Funct. Mater. 2023, 32, 2201166.

[38] Z. Fan , J. Jin , C. Li , J. Cai , C. Wei , Y. Shao , G. Zou , J. Sun , ACS Nano 2021, 15, 3098. 33576601 10.1021/acsnano.0c09646

[39] H. Lu , J. Hu , Y. Zhang , K. Zhang , X. Yan , H. Li , J. Li , Y. Li , J. Zhao , B. Xu , Adv. Mater. 2023, 35, 2209886. 10.1002/adma.202209886 36515180

[40] B. Wu , B. Guo , Y. Chen , Y. Mu , H. Qu , M. Lin , J. Bai , T. Zhao , L. Zeng , Energy Storage Mater. 2023, 54, 75.

[41] Y. Liu , S. Zheng , J. Ma , X. Wang , L. Zhang , P. Das , K. Wang , Z.‐S. Wu , Adv. Energy Mater. 2022, 12, 2200341.

[42] N. Zhang , M. Jia , Y. Dong , Y. Y. Wang , J. Z. Xu , Y. C. Liu , L. F. Jiao , F. Y. Cheng , Adv. Funct. Mater. 2019, 29, 1807331.

[43] Z. Zhang , Y. Shen , Z. Zhao , S. Li , Q. Wang , C. Zhong , W. Hu , J. Power Sources 2022, 542, 231815.

[44] J. Li , K. McColl , X. Lu , S. Sathasivam , H. Dong , L. Kang , Z. Li , S. Zhao , A. G. Kafizas , R. Wang , D. J. L. Brett , P. R. Shearing , F. Corà , G. He , C. J. Carmalt , I. P. Parkin , Adv. Energy. Mater. 2020, 10, 2000058.

[45] G. Yang , C. Wang , Energy Environ. Mater. 2021, 4, 596.

[46] G. Zhang , X. Zhang , H. Liu , J. Li , Y. Chen , H. Duan , Adv. Energy. Mater. 2021, 11, 2003927.

[47] H. Tian , Z. Li , G. Feng , Z. Yang , D. Fox , M. Wang , H. Zhou , L. Zhai , A. Kushima , Y. Du , Z. Feng , X. Shan , Y. Yang , Nat. Commun. 2021, 12, 237. 33431888 10.1038/s41467-020-20334-6 PMC7801520

[48] A. Chen , C. Zhao , Z. Guo , X. Lu , J. Zhang , N. Liu , Y. Zhang , N. Zhang , Adv. Funct. Mater. 2022, 32, 2203595.

[49] L. Zeng , H. He , H. Chen , D. Luo , J. He , C. Zhang , Adv. Energy. Mater. 2022, 12, 2103708.

[50] G. Nagaraju , C. Sekhar , B. Ramulu , J. S. Yu , Energy Storage Mater. 2021, 35, 750.

[51] L. Xu , Y. Zhang , J. Zheng , H. Jiang , T. Hu , C. Meng , Mater. Today Energy 2020, 18, 100509.

[52] L. Wang , M. Peng , J. Chen , X. Tang , L. Li , T. Hu , K. Yuan , Y. Chen , ACS Nano 2022, 16, 2877. 35129326 10.1021/acsnano.1c09936

[53] H. Hosseini , S. Shahrokhian , Chem. Eng. J. 2019, 375, 122090.

[54] Y. An , Y. Tian , Q. Man , H. Shen , C. Liu , Y.i. Qian , S. Xiong , J. Feng , Y. Qian , ACS Nano 2022, 16, 6755. 35357131 10.1021/acsnano.2c01571

[55] H. Hosseini , S. Shahrokhian , Appl. Mater. Today 2018, 10, 72.

[56] Z. Ge , L. Xu , Y. Xu , J. Wu , Z. Geng , X. Xiao , W. Deng , G. Zou , H. Hou , X. Ji , Nano Energy 2024, 119, 109053.

[57] K. Zhu , T. Wu , S. Sun , W. van den Bergh , M. Stefik , K. Huang , Energy Storage Mater. 2020, 29, 60.

[58] S. Liu , H. Zhu , B. Zhang , G. Li , H. Zhu , Y. Ren , H. Geng , Y. Yang , Q. Liu , C. C. Li , Adv. Mater. 2020, 32, 2001113. 10.1002/adma.202001113 32431024

[59] N. Zhang , M. Jia , Y. Dong , Y. Y. Wang , J. Z. Xu , Y. C. Liu , L. F. Jiao , F. Y. Cheng , Adv. Funct. Mater. 2019, 29, 1807331.

[60] C. Xia , J. Guo , Y. Lei , H. Liang , C. Zhao , H. N. Alshareef , Adv. Mater. 2018, 30, 1705580. 10.1002/adma.201705580 29226488

[61] N. Zhang , Y. Dong , M. Jia , X. Bian , Y. Wang , M. Qiu , J. Xu , Y. Liu , L. Jiao , F. Cheng , ACS Energy Lett. 2018, 3, 1366.

[62] Q. Pang , C. Sun , Y. Yu , K. Zhao , Z. Zhang , P. M. Voyles , G. Chen , Y. Wei , X. Wang , Adv. Energy Mater. 2018, 8, 1800144.

[63] C. Li , R. Kingsbury , A. S. Thind , A. Shyamsunder , T. T. Fister , R. F. Klie , K. A. Persson , L. F. Nazar , Nat. Commun. 2023, 14, 3067. 37244907 10.1038/s41467-023-38460-2 PMC10224959

