# Supplementary material for: 3D Printed Low‐Tortuosity and Ultra‐Thick Hierarchical Porous Electrodes for High‐Performance Wearable Quasi‐Solid‐State Zn‐VOH Batteries
Source: Adv Sci (Weinh). 2025 Mar 5;12(16):2401660. doi: 10.1002/advs.202401660 (PMC12021079; doi:10.1002/advs.202401660)
Supplement: Supplementary file 1 — Supporting Information [file ADVS-12-2401660-s001.docx]

**Supporting information**

**3D Printed Low-Tortuosity and Ultra-Thick Hierarchical Porous Electrodes** **for High-Performance Wearable Quasi-Solid-State Zn-VOH Batteries**

Qingguo Xu,^a^ Ningning Chu,^a^ Ye Wang,^a^ Hui Wang,^a^ Tingting Xu,^a^ Xueliang Li,^b^ Shaozhuan Huang,^d^ Xinjian Li,^a^ Yongsong Luo,^c,^*** Hui Ying Yang,^b,^*** and Dezhi Kong ^a,^***

^a^ Key Laboratory of Material Physics of Ministry of Education, School of Physics and Microelectronics, Zhengzhou University, Zhengzhou 450052, China

^b^ Pillar of Engineering Product Development, Singapore University of Technology and Design, 8 Somapah Road, Singapore 487372, Singapore

^c^ Henan International Joint Laboratory of MXene Materials Microstructure, College of Physics and Electronic Engineering, Nanyang Normal University, Nanyang 473061, China

^d^ Hubei Key Laboratory of Catalysis and Materials Science, South-Central University for Nationalities, Wuhan, Hubei 430074, China

* Corresponding author.

E-mail: *yanghuiying@sutd.edu.sg*; *ysluo@xynu.edu.cn*; *dezhi_kong@zzu.edu.cn*

**Part I: Supporting Experimental** **Section**

*Preparation of GO/CNT ink*：In a typical GO/CNT ink preparation procedure, a proper amount of CNT powder (500 mg) is dispersed in diluted GO suspension (200 mL, 5 mg/mL), and then the CNTs are uniformly distributed in GO suspension. Then, a proper amount of water is removed by a high-speed centrifuge until the obtained high-viscosity ink maintains its three-dimensional structure at different angles on the glass substrate. Finally, the GO/CNT ink was transferred to a 15 mL syringe and extruded to eliminate bubbles, and then the ink was extruded using a micro-printing nozzle (inner diameter: D = 0.08 mm) to print different patterns on the glass substrate. Synthesis of 3D printed GO/CNTs microlattices shortly after printing the pattern, the 3D printed pattern was immersed in liquid nitrogen and then freeze-dried for 2 days to form aerogel. Annealing in a tube furnace at 650 °C for 3 h under argon to form a 3D printed rGO/CNT aerogel microlattices, with a heating rate of 2.5 ℃/min.

*Rheological Measurement*: All measurements were carried out using 3.0 cm diameter steel parallel-plate geometry with a solvent trap to prevent evaporation of water. The temperature was controlled at 25 °C during the entire tests. An amplitude stress sweep from 10 to 1000 Pa was performed at a constant frequency of 1 Hz to get storage modulus and loss modulus as a function of shear stress. A frequency sweep was subsequently carried out at a fixed strain of 0.1 % (the amplitude value selected within the linear viscoelastic (LVE) region is determined by previous amplitude stress sweep test) with an angular frequency range from 0.1 to 100 rad s^-1^. A shear-rate-controlled test was also conducted using ascending logarithmic steps to record the viscosity as a function of shear rate (0.01~200 s^-1^).

**Power law model:**

The obtained shear stress as a function of shear rate in steady shearing experiments could fitted by the Power law model, as shown in the Equation [1]:

Where *τ* (Pa) is the shear stress, *γ* (s^-1^) is the shear rate, *K* is the consistency coefficient, and *n* is the flow behavior index, respectively.

**Part II: Calculations**

The capacitance values were calculated from the discharge time of galvanostatic charge-discharge (GCD) profiles according to the following equation 2 [2]:

where *C*_Areal_ (F cm^-2^) is denoted as the areal capacitance of zinc ion hybrid capacitors (ZIHCs), ΔV (V) is the voltage window of the cell, t (s) is the discharge time, A (cm^2^) is the area of the device including the gaps, and I (A) is the current.

The energy density and power density of ZIHCs were calculated based on the areal capacitance of the device according to the below equation 3 [3]:

where *E*_Areal_ (Wh cm^-2^) is the areal energy density based on the area of the device.

The power density of the device was calculated from the equation 4 [4]:

where *P*_Areal_ (W cm^-2^) is the areal power density based on the area of the device.

**Part III: Supporting Figures**

**
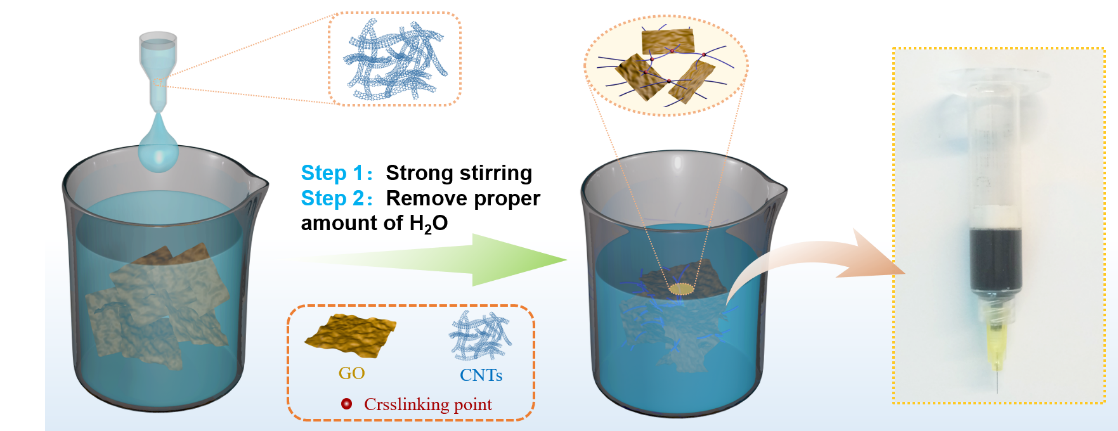
**

**Figure S1**. A proper amount of MWCNTs paste were dispersed in the dilute GO suspension, followed by strong stirring to achieve uniform distribution of MWCNTs within the GO suspension (Step I); and then via high speed centrifuge to remove proper amount of water (Step II); finally, the GO/CNTs ink is transferred to a 10 mL printing syringe (Step III).

To enable DIW printing, GO inks should conform to three important criteria. First, the ink must exhibit a relatively low elastic shear modulus under high shear stress, so it can flow stably passing through the deposition nozzle. Second, the static elastic modulus of the ink should be large enough to make the extruded filament “set” immediately, maintaining the shape with deposited features and defying the collapse caused by the self-weight and surface tension. Third, the ink needs to keep homogeneous to prevent clogging in the nozzle. To address these requirements, we designed GO/CNTs hydrogels with suitable rheological behaviors as printing inks for DIW. In a typical process, GO/CNTs DIW inks were simply prepared by adding trace of CNTs (5-10×10^-3^ m) into GO aqueous solution (8-20 mg mL^-1^) with constantly mechanical stirring (Figure S1). GO sheet possesses various oxygen-containing functional groups such as -COOH and -OH, therefore, CNTs can act as cross-linkers to form hydrogel through a coordination interaction with these functional groups of GO. The rheological behaviors of GO hydrogels were easily controlled by adjusting the dose of CNTs.


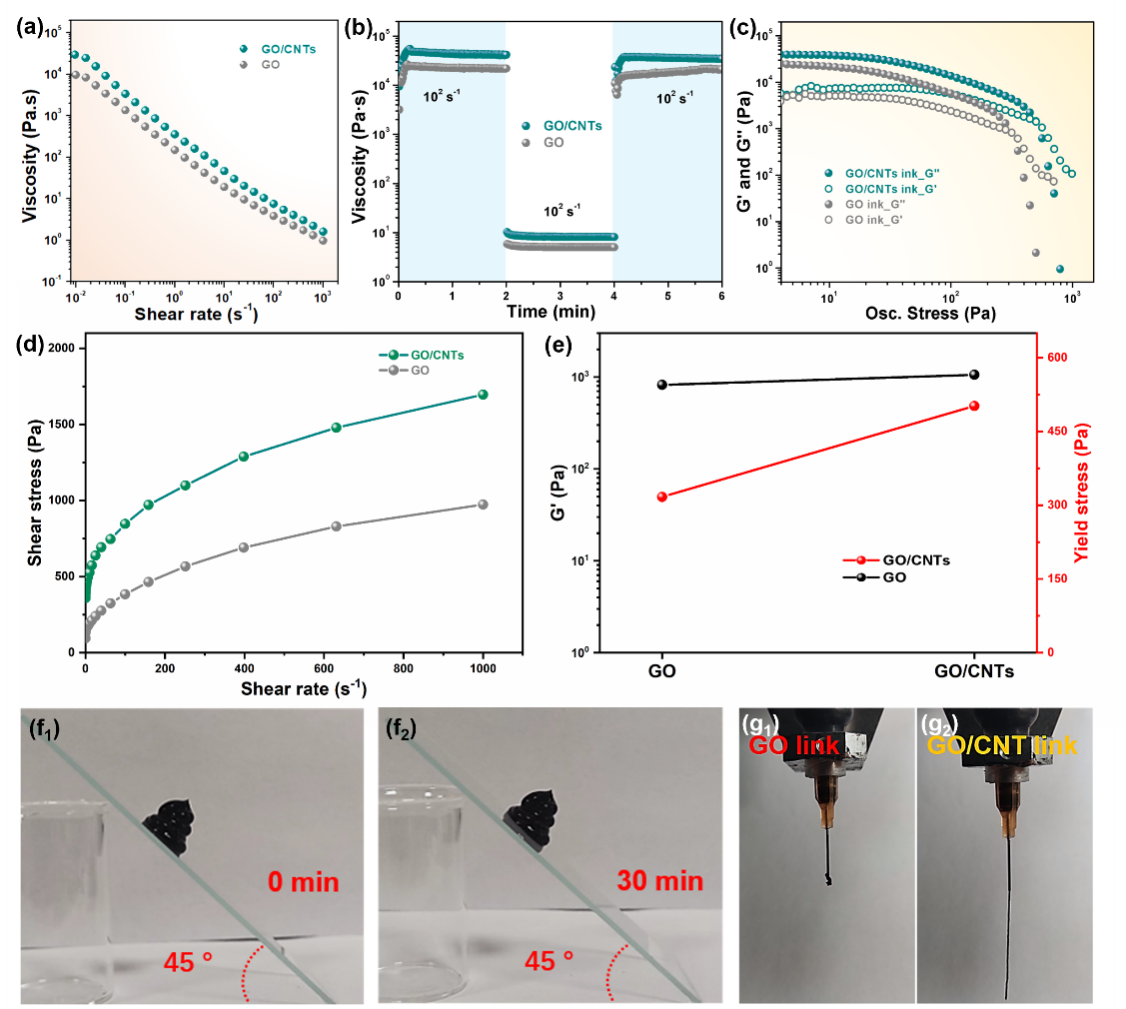


**Figure S2**. Rheology properties, 3D printed patterns and morphology characterizations. (a) Rheological behaviors of the GO pure solution and GO/CNTs mixed gel inks. (b) Viscosity evolution over time for alternating low (10^-2^ s^-1^) and high shear rates (10^2^ s^-1^). (c) G′ and G″ as a function of oscillatory stress. Photos of 3D printed rGO/CNTs microlattice. (d) Shear stress as a function of shear rate, and the curves are fitted by the power law. (e) G′ at plateau regions and yield stress obtained at the crossover point between G′ and G″. (f_1_) Digital photo showing the printed patterns using rGO/CNTs inks at the initial stage. Digitalphotos showing the printed patterns on a glass substrate at an incline of 45° after 30 min (f_2_). (g) Digital appearance of GO and GO/CNTs inks upon extruding.

Central to the extrusion-based 3D printing technology is the development of printable inks with excellent rheological properties, including significant shear-thinning behavior, sufficient initial storage modulus (*G'*) and yield stress (*τ*_y_).[5] As shown in Figure S2a, both pure GO solution and GO/CNTs mixed gel inks exhibit the same shear-thinning non-Newtonian fluid behavior, that is, the viscosity decreases with increasing shear rate. It is worth noting that the GO ink based on the addition of CNTs displays a much higher apparent viscosity with a shear-thinning non-Newtonian behavior. The curves of shear stress versus shear rate were well fitted by the power law (Figure S2d), all samples show shear thinning behavior, which is conducive to the continuous extrusion of ink from the needle. Remarkably, it was also observed that the initial viscosity of the sample gradually increased with the addition of CNTs, which was due to the enhancement of the cross-linking network between CNTs and GO nanosheets (Figure S2e and S2f).[6] The thixotropic behavior was analyzed by monitoring the change in viscosity when the low and high shear rates were alternately applied, as shown in Figure S2b. Originally, a very low shear rate of 10^-2^ s^-1^ was applied to simulate the pre-extrusion conditions. After being continuously shear-mixed for 2 min, the shear rate was raised to 10^2^ s^-1^, and the according viscosity decreases rapidly, simulating the extrusion process of inks through a narrow nozzle. At this stage, the GO/CNTs gel network is destroyed, and its structural units are aligned in the direction of flow in response to shear stress, thus exhibiting a strong shear thinning behavior that allows ink to flow out of the nozzle. Finally, when the shear rate returned to 10^-2^ s^-1^, the viscosity immediately recovered due to the recovery of the GO/CNTs gel network. Meanwhile, both inks show different viscosity recovery percentages depending on the loading of CNTs and recovery time. Within 2 min recovery time, the viscosity recovery percentage of GO and GO/CNTs ink are 75.4% and 92.8%, respectively. These observations indicate that the GO/CNTs inks can quickly restore to their original state and maintain a stable printing structure after being extruded during 3D printing. Figure S2c exhibits the storage modulus (G′) and loss modulus (G″) of different samples as a function of shear strain. The storage modulus (G′) and loss modulus (G″) of the GO/CNT ink are higher than GO ink, indicating the GO/CNT ink has a stiffer feature, which is an ideal slurry for printing self-supported structures without deformation.[7] The size of G' in the platform area (i.e., the area where G' is almost independent of stress), and the yield stress increases with the addition of CNTs (Figure S2e). Especially for the GO/CNTs ink, the G′ at the plateau region and yield stress reach the highest values of 1058.51 and 501.86 Pa, respectively. The viscosity test on the prepared GO/CNTs ink is shown in Figure S2f. Upon extrusion, the pure GO ink with low viscosity generated droplets (Figure S2g_1_), leading to discontinuous flow, i.e., poor extrudability. In contrast, the uniform, continuous filament was extruded using the GO/CNTs ink (Figure S2g_2_) ascribing to its homogeneity and improved rheological performance. All in all, the high modulus and yield stress of GO/CNTs inks represent a more rigid property, which is required for the construction of self-supported ordered structures without deformation.


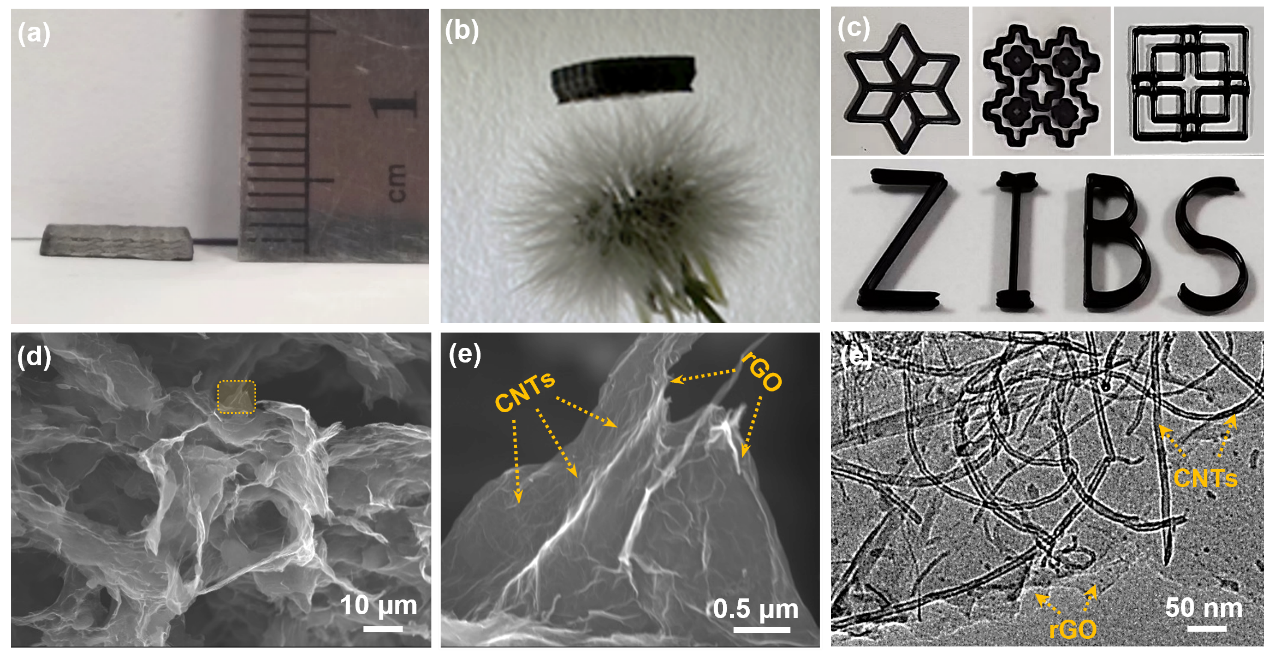


**Figure S3**. Photos of 3D printed rGO/CNTs microlattice (a) side view. (b) Optical images showing 3D printed rGO/CNTs microlattices electrodes with a thickness of 1.5 mm can be placed on dandelion flowers. (c) Photos of 3D printed rGO/CNTs microlattices electrodes. (d, e) SEM images of 3D printed rGO/CNTs microlattices electrodes and their gradient porous structures. (e) TEM images of 3D printed rGO/CNTs microcrystalline structure.

**
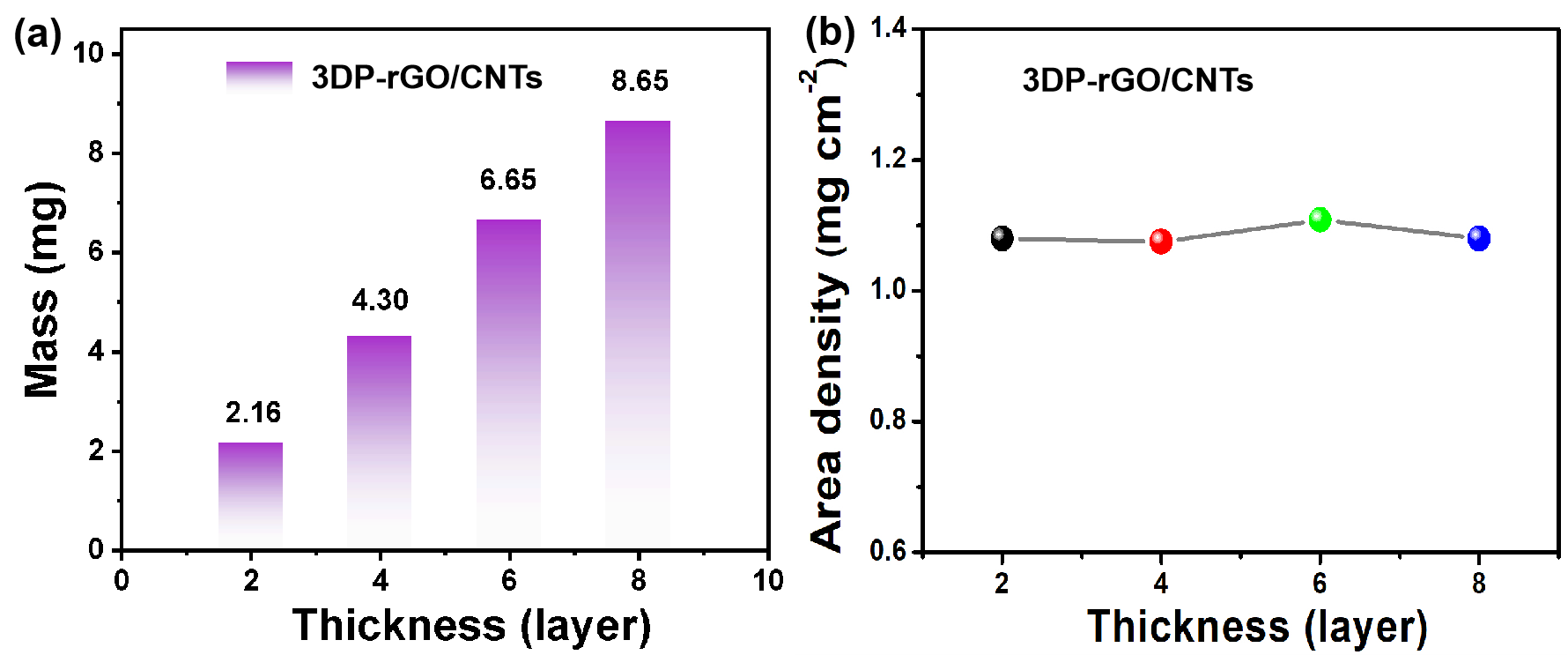
**

**Figure S4**. (a) Relationship between the total mass of 3DP-rGO/CNTs microlattices and the layer thickness of 3D printed microlattices. (b) Relationship between the area density of 3DP-rGO/CNTs microlattices and the layer thickness of 3D printed microlattices.


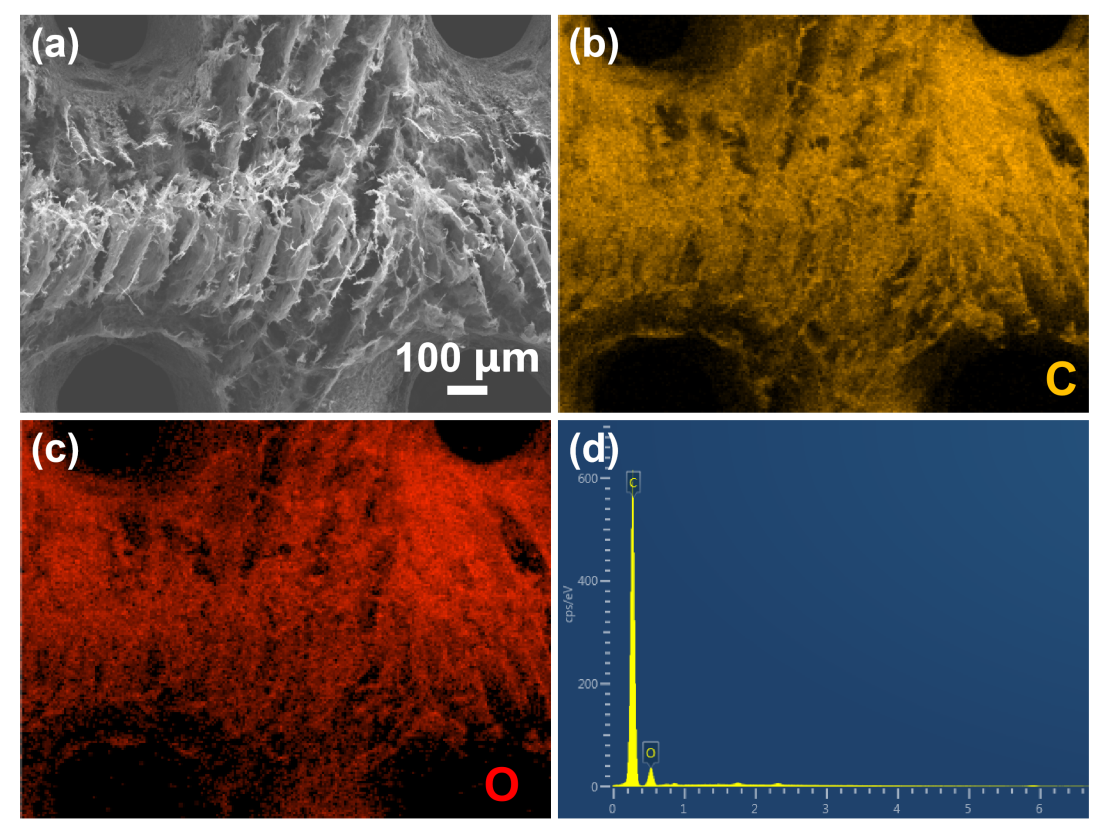


**Figure S5**. SEM image, element maps of C, and O for 3DP-rGO/CNTs microlattices.

**
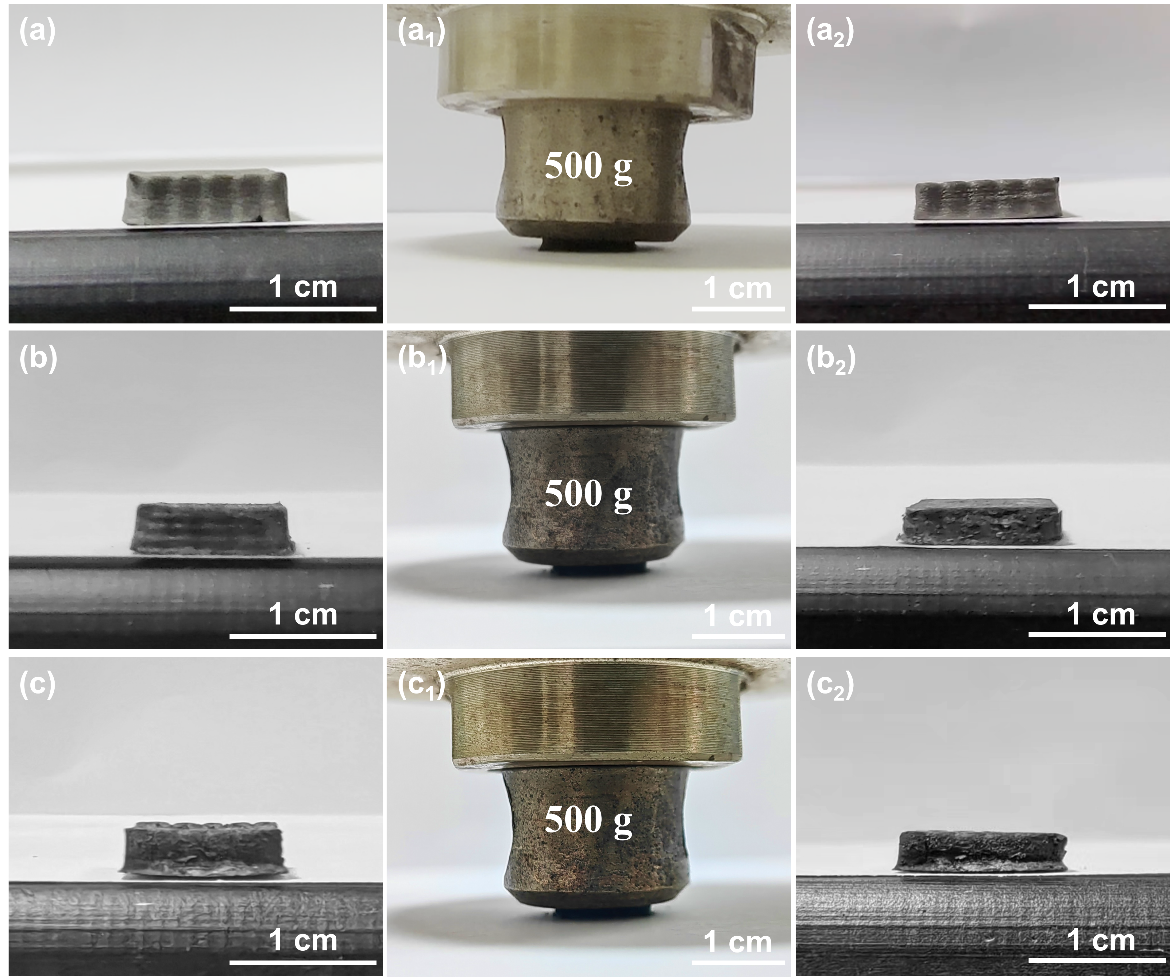
**

**Figure S6**. Photographs of the (a) 3DP-rGO/CNTs, (b) Zn@3DP-rGO/CNTs, (c) VOH@3DP-rGO/CNTs microlattices electrodes between initial states and after the repeated compression tests.


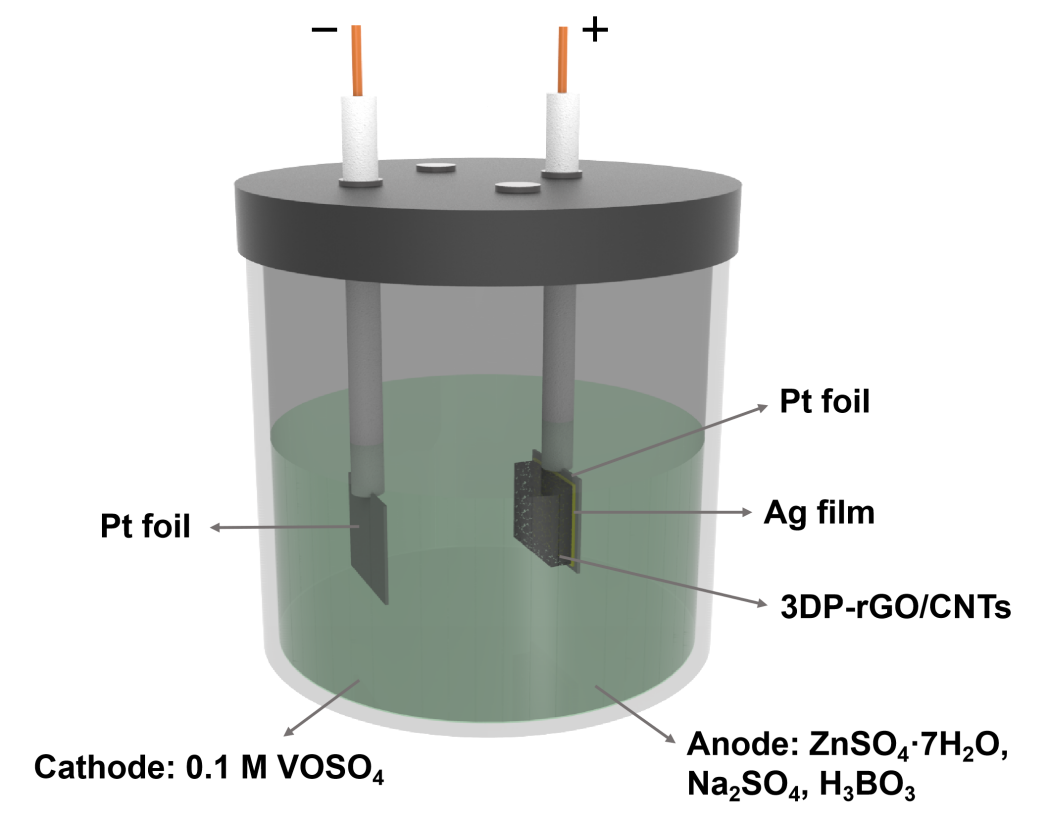


**Figure S7**. A schematic diagram of electrodeposition device based on two-electrode system.

**
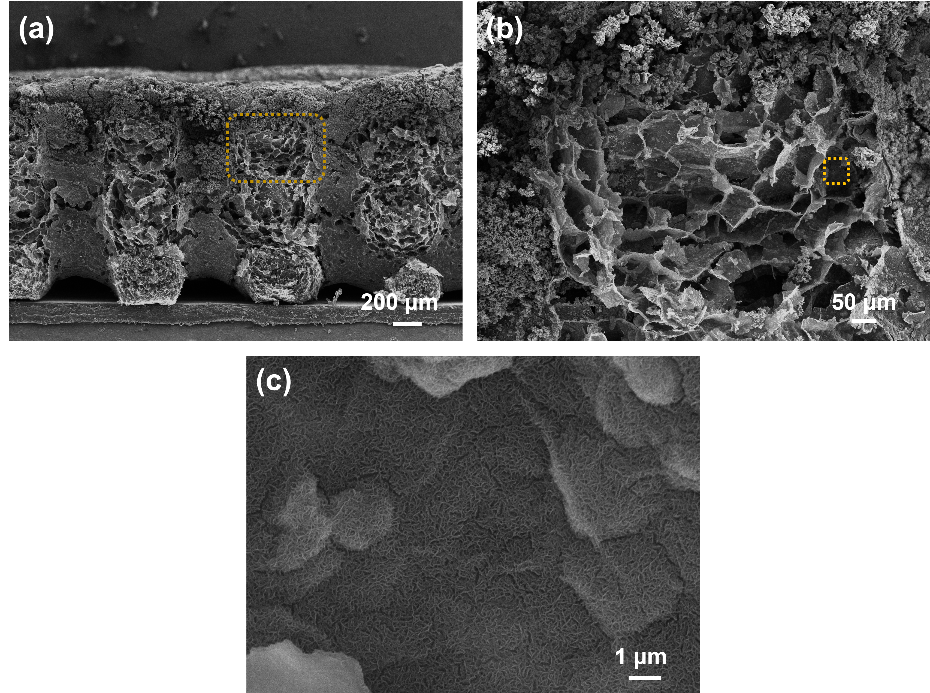
**

**Figure S8**. SEM images collected from the interior of VOH@3DP-rGO/CNTs microlattices.

**
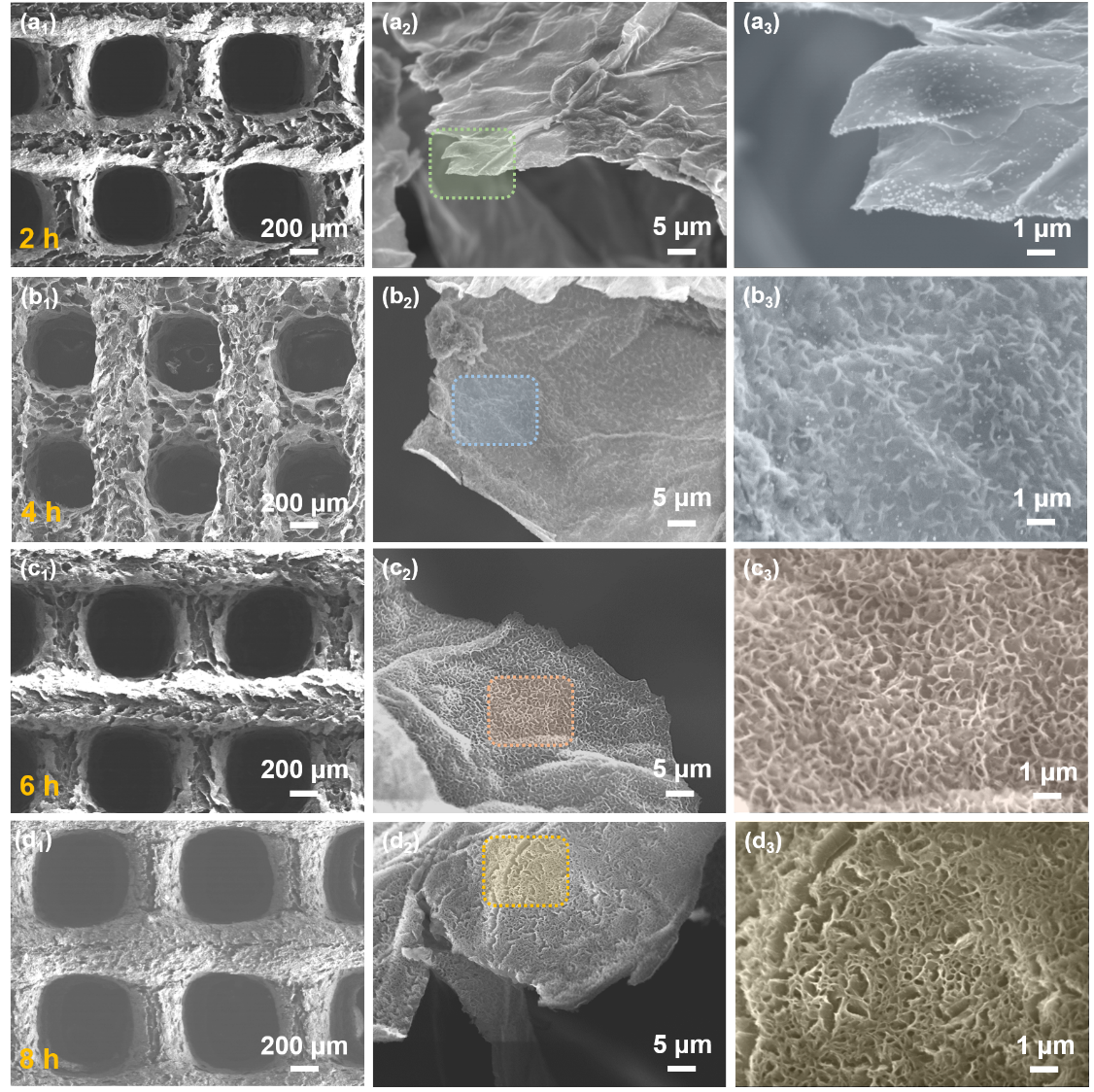
**

**Figure S9**. SEM images of the prepared VOH@3DP-rGO/CNTs samples with different electrodeposition times of (a_1_-a_3_) 2h, (b_1_-b_3_) 4 h, (c_1_-c_3_) 6 h, and (d_1_-d_3_) 8 h.


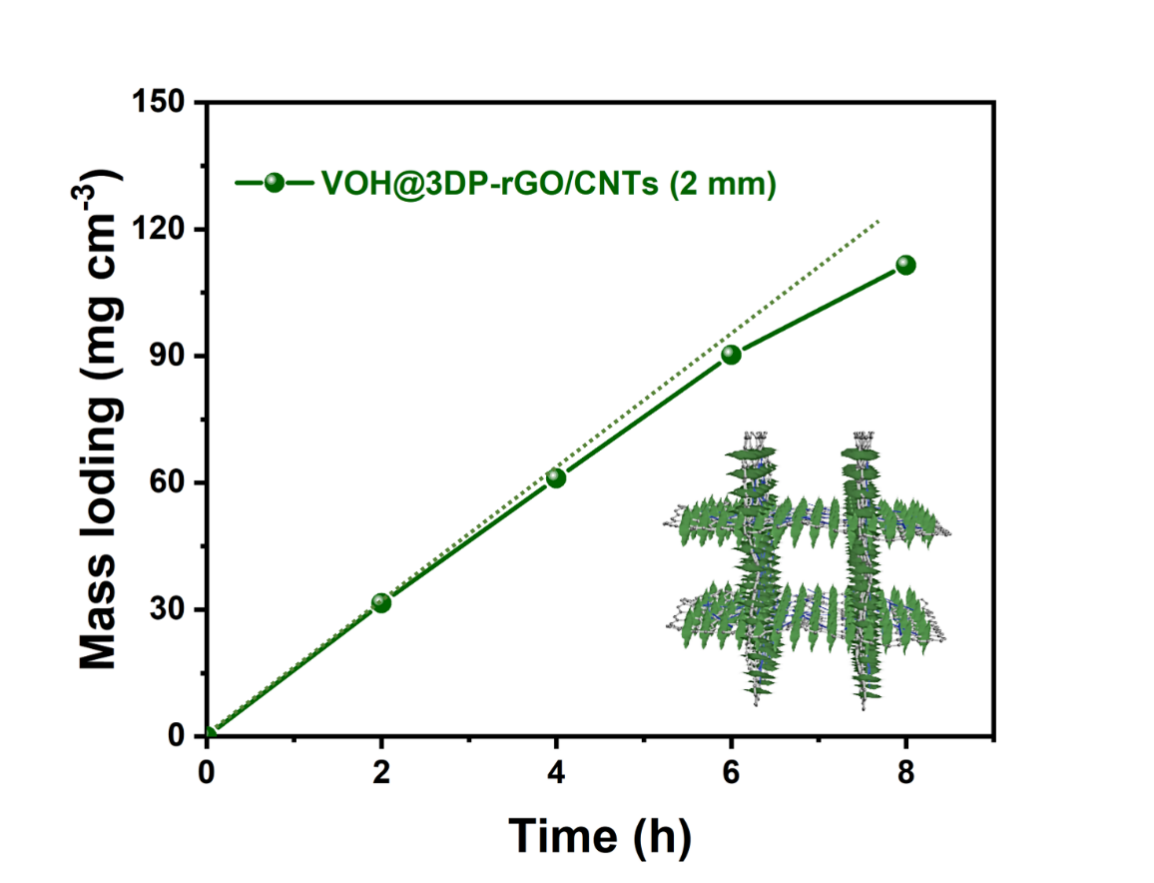


**Figure S10**. The mass loading of VOH nanosheets on the 3DP-rGO/CNTs hybrid aerogel lattices increase with the deposition time from 0 h to 8 h.

**
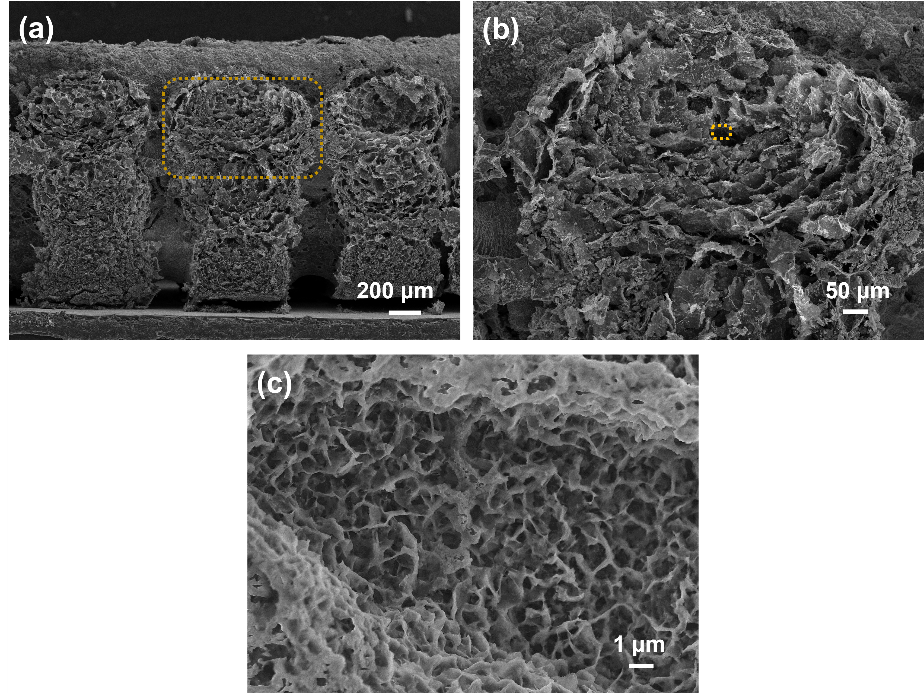
**

**Figure S11**. SEM images collected from the interior of Zn@3DP-rGO/CNTs microlattices.

**
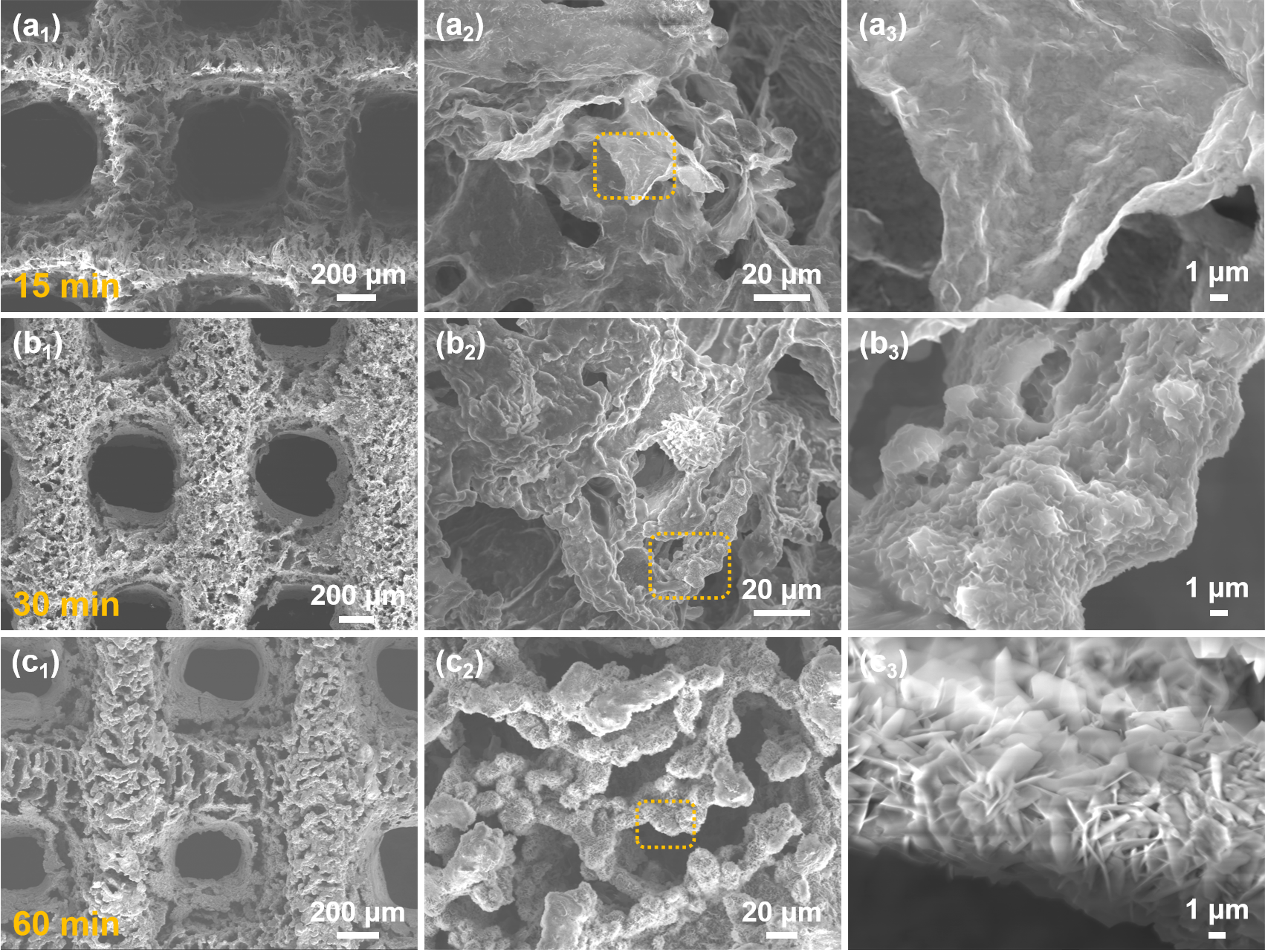
**

**Figure S12**. SEM images of the Zn@3DP-rGO/CNTs samples prepared under different electrodeposition times of (a_1_-a_3_) 15 min, (b_1_-b_3_) 30 min and (c_1_-c_3_) 60 min.


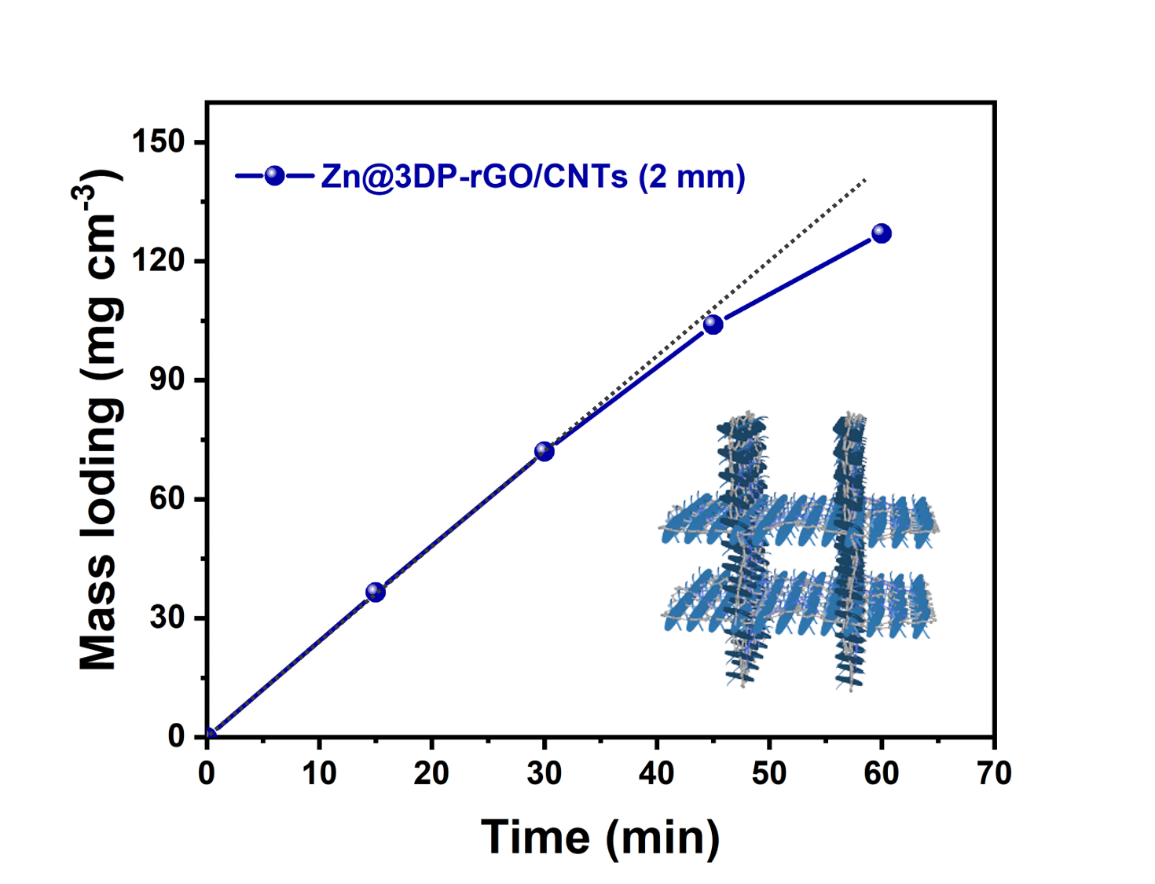


**Figure S13**. The mass loading of Zn nanoflakes on the 3DP-rGO/CNTs hybrid aerogel lattices increase with the deposition time from 0 min to 60 min.


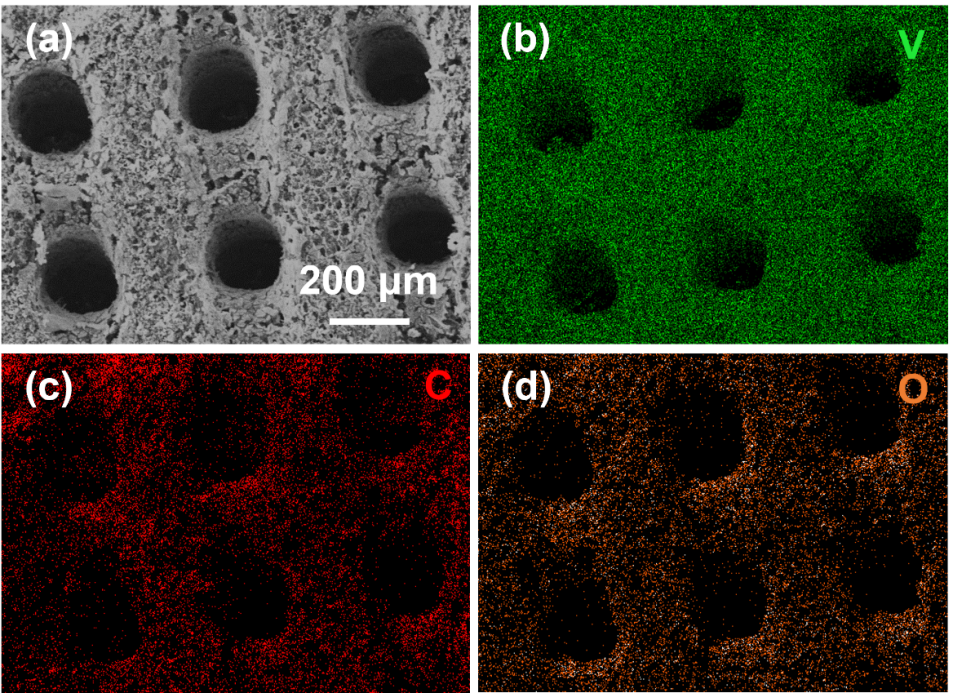


**Figure S14**. The mass loading of Zn nanoflakes on the 3DP-rGO/CNTs hybrid aerogel lattices increase with the deposition time from 0 min to 60 min.


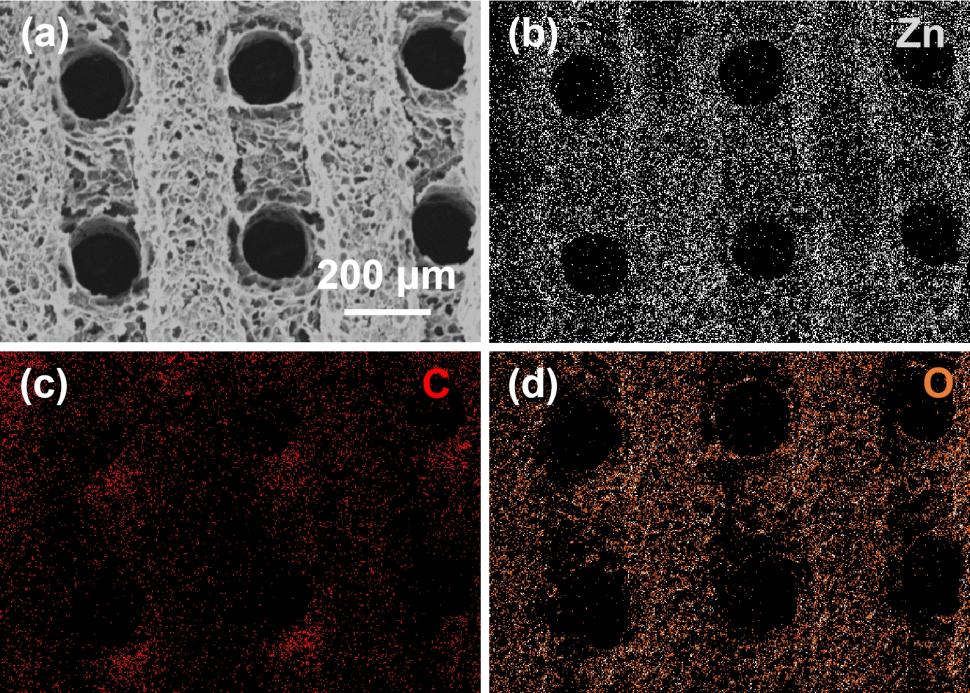


**Figure S15**. The mass loading of Zn nanoflakes on the 3DP-rGO/CNTs hybrid aerogel lattices increases with the deposition time from 0 min to 60 min.





**Figure S16**. XRD of the VOH@3DP-rGO/CNTs samples prepared under different electrodeposition times of 6 h and 8 h.

**
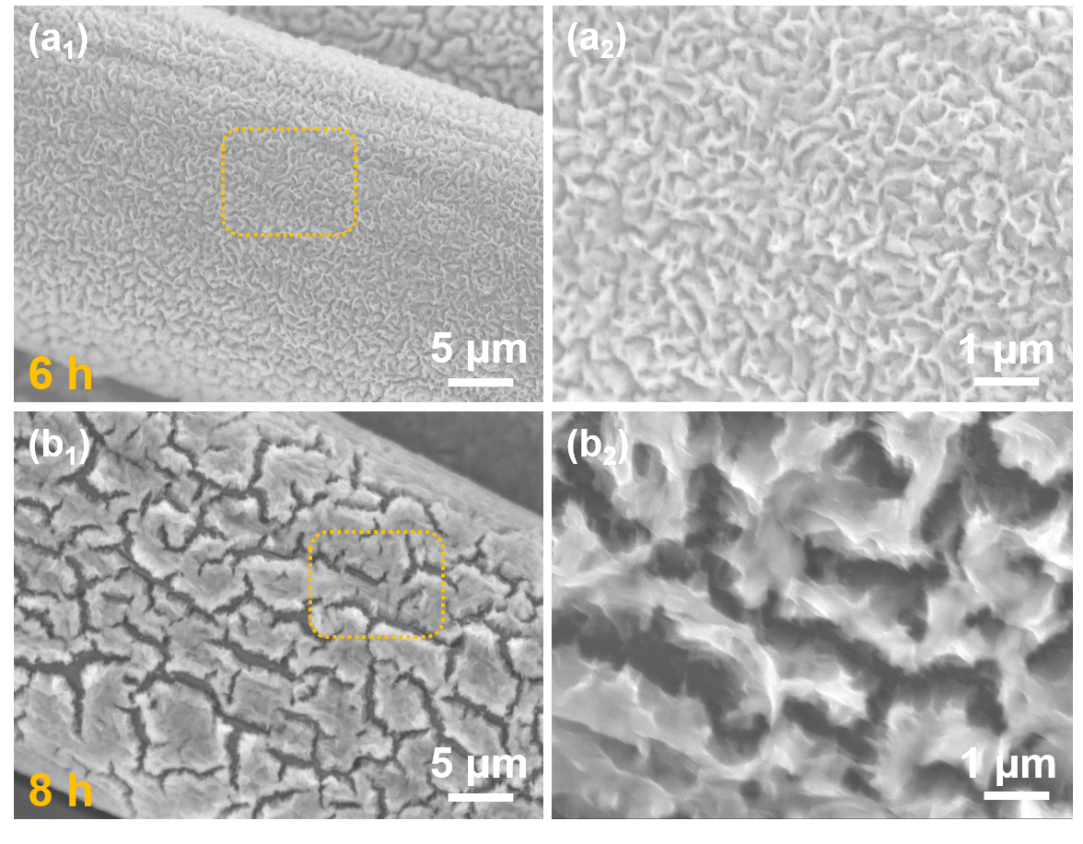
**

**Figure S17**. SEM images of the VOH@CTs samples prepared under different electrodeposition times of (a_1_,a_2_) 6 h, (b_1_,b_2_) 8 h.


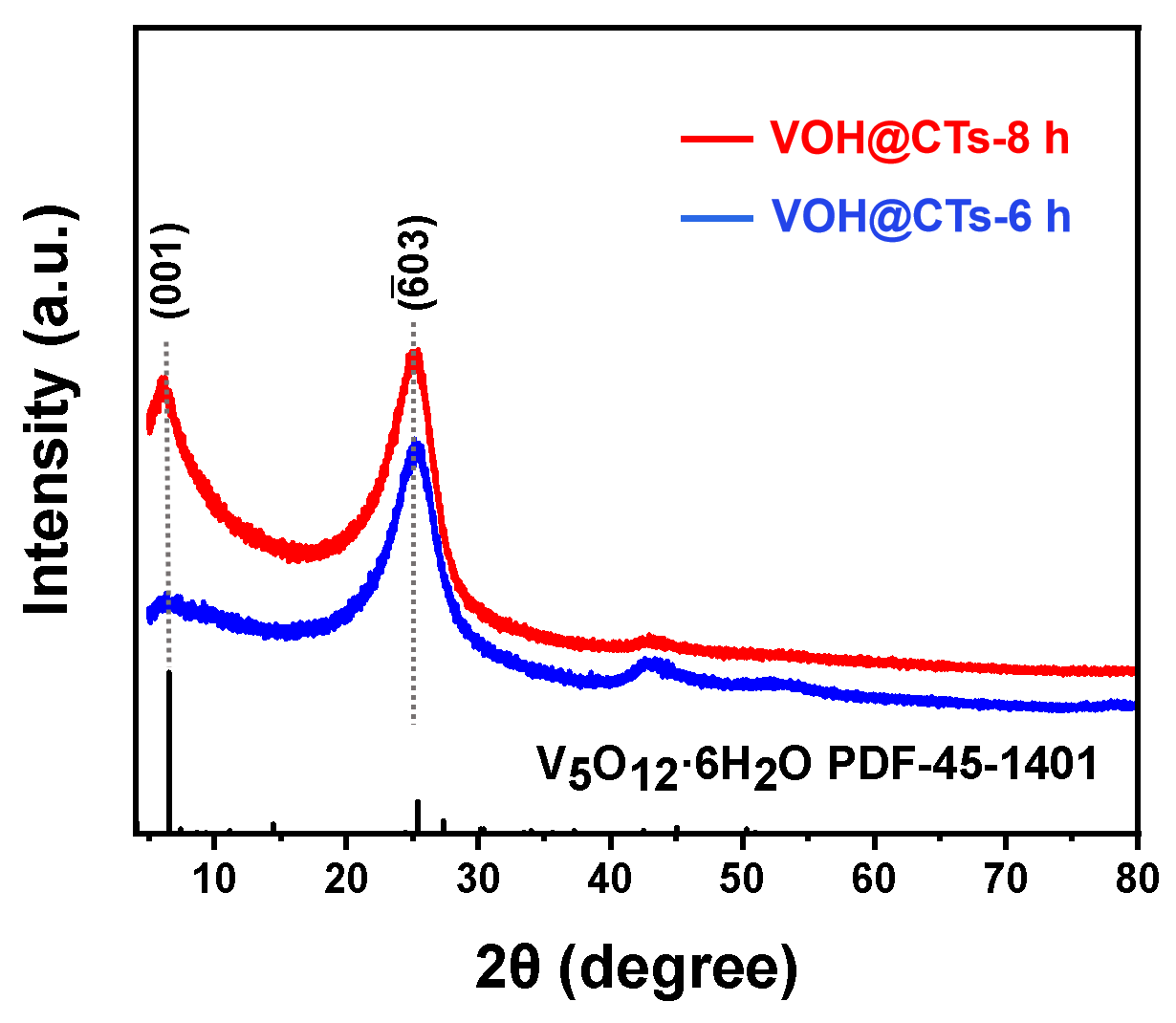


**Figure S18**. XRD of the VOH@CTs samples prepared under different electrodeposition times of 6 h and 8 h.

**
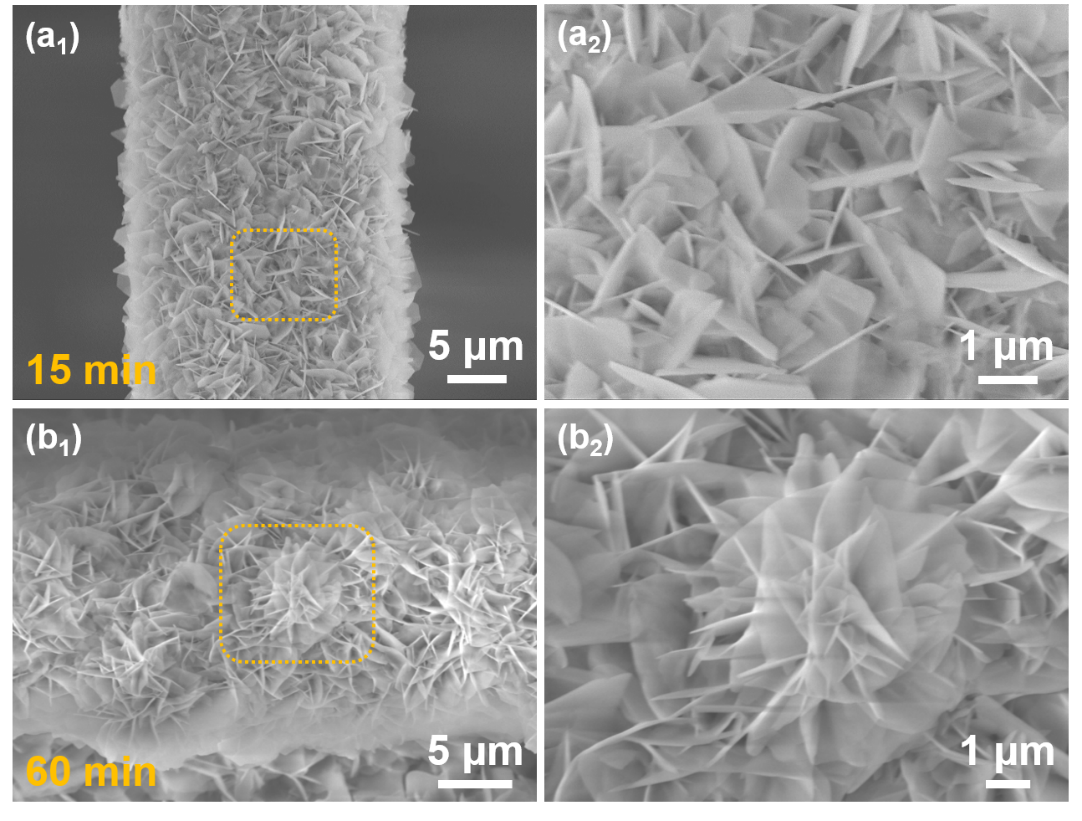
**

**Figure S19**. SEM images of the Zn@CTs samples prepared under electrodeposition times of (a_1_,a_2_) 15 min and (b_1_,b_2_) 60 min.


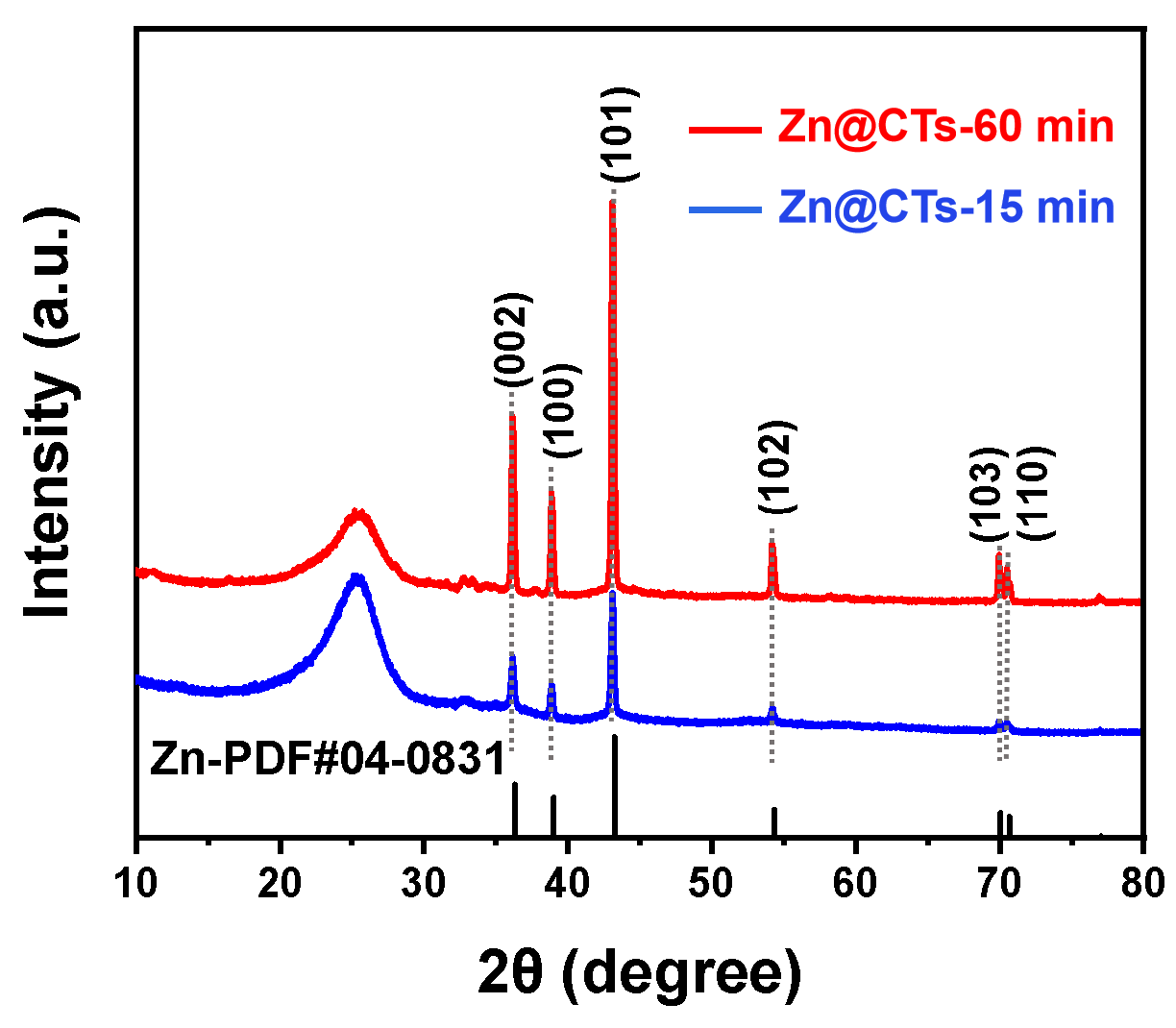


**Figure S20**. XRD of the Zn@CTs samples prepared under different electrodeposition times of 15 min and 60 min.

**
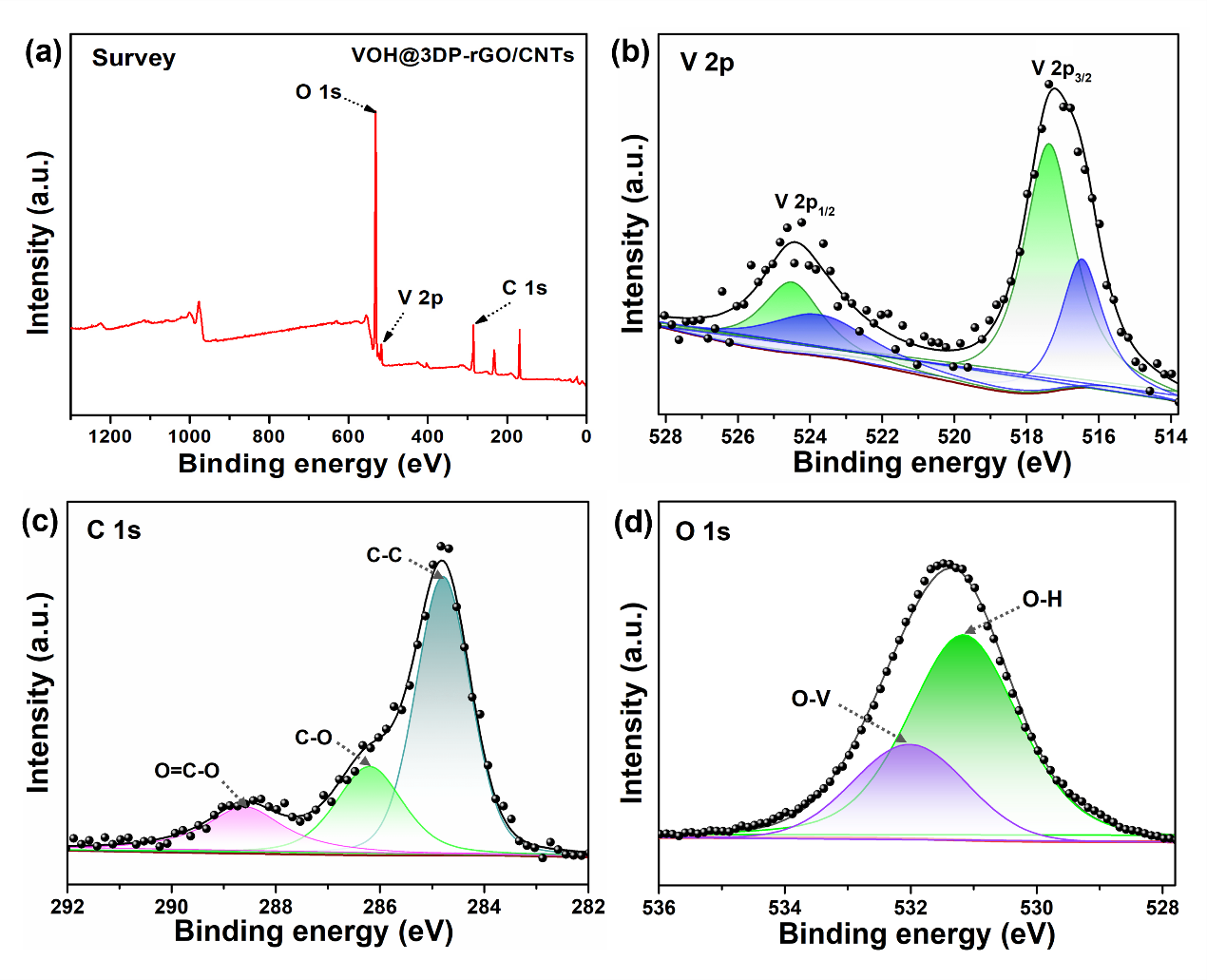
**

**Figure S21**. **(**a) XPS survey spectra of VOH@3DP-rGO/CNTs microlattices electrodes. XPS spectra of b) V 2p, (c) C 1s, (d) O 1s of the VOH@3DP-rGO/CNTs.

The elemental components and surface electronic states of the VOH@3DP-rGO/CNTs sample were investigated by X-ray photoelectron spectroscopy (XPS) analysis. As presented in Figure S21a and S22a, the survey spectrum indicates the existence of V, C, and O elements, which is well consistent with the result of elemental mapping (Figure S14). For the V 2p spectrum (Figure S21b), the fitted peaks at 524.5 and 517.4 eV are ascribed to the 2p_1/2_ and 2p_3/2_ of V^5+^, and the V^4+^ peaks of 523.8 and 516.5 eV imply the generation of the lower valence state of vanadium.[8] The C 1s spectra (Figure S21c) at 284.6 eV, 286.4 eV and 288.6 eV correspond to C-C, C-O and O=C-O, respectively.[9] The O 1s spectrum in Figure S21d displays two peaks with binding energies of 531.2 eV and 532.1 eV, belonging to O in O-V and O-H, respectively.[10]


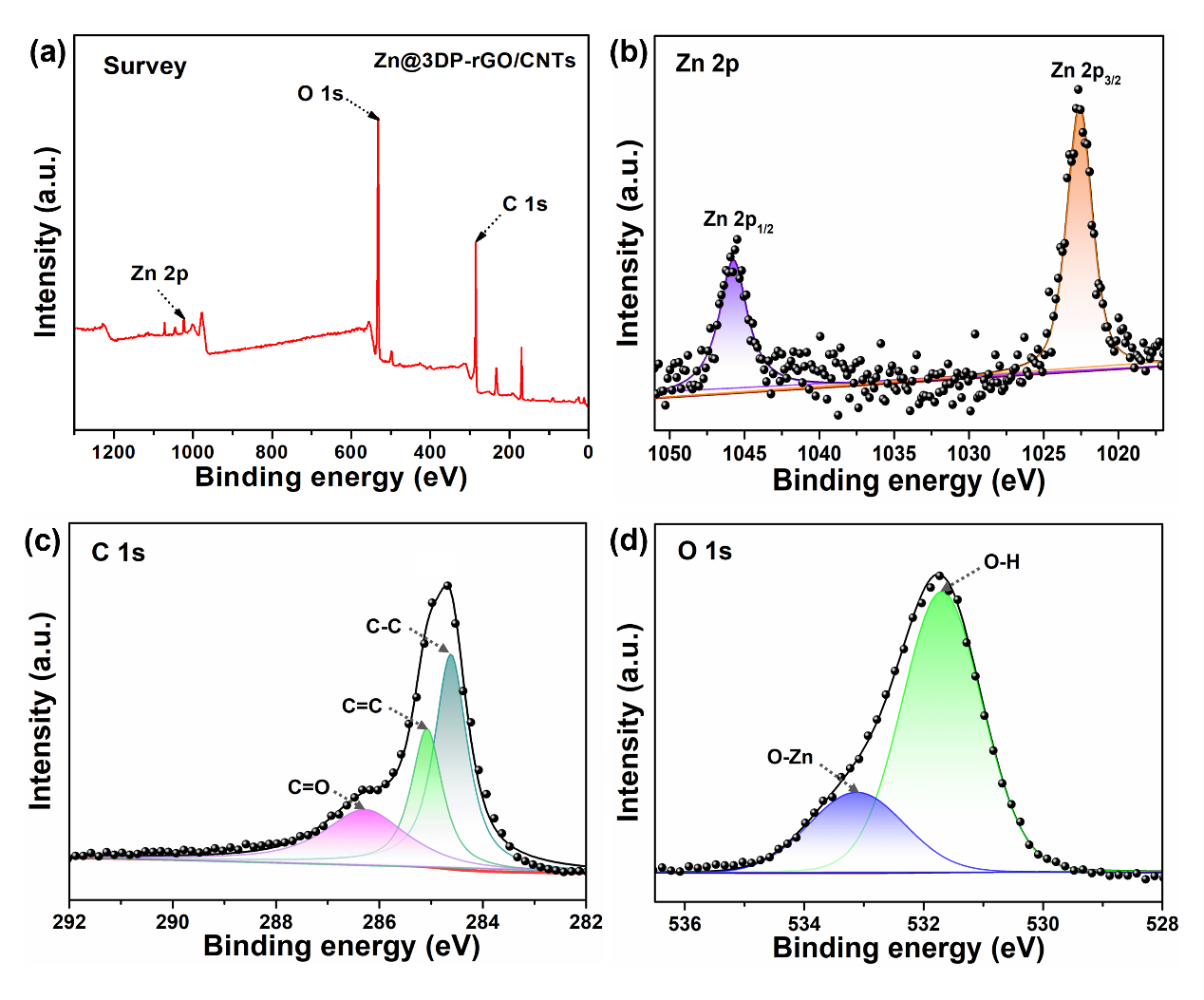


**Figure S22**. **(**a) XPS survey spectra of Zn@3DP-rGO/CNTs microlattices electrodes. XPS spectra of (b) Zn 2p, (c) C 1s, (d) O 1s of the Zn@3DP-rGO/CNTs.

The elemental components and surface electronic states of the Zn@3DP-rGO/CNTs sample were investigated by X-ray photoelectron spectroscopy (XPS) analysis. The XPS survey spectrum in Figure S22a indicates that there are only Zn, C, and O elements in the composite sample, which is also flawlessly consistent with the result of elemental mapping (Figure 15). The Zn 2p spectrum (Figure S22b) presents two typical peaks at 1022.68 and 1045.38 eV, ascribing to Zn 2p_3/2_ and Zn 2p_1/2_ of Zn.[11] The C 1s spectrum (Figure S22c) can be resolved into three peaks that were C-C (284.6 eV), C=C (285 eV) and C=O (286.3 eV). [9] The O 1s spectrum (Figure S22d) can be divided into two peaks (531.2 eV and 533.5 eV), belonging to O-Zn and O-H, respectively.[12]

**
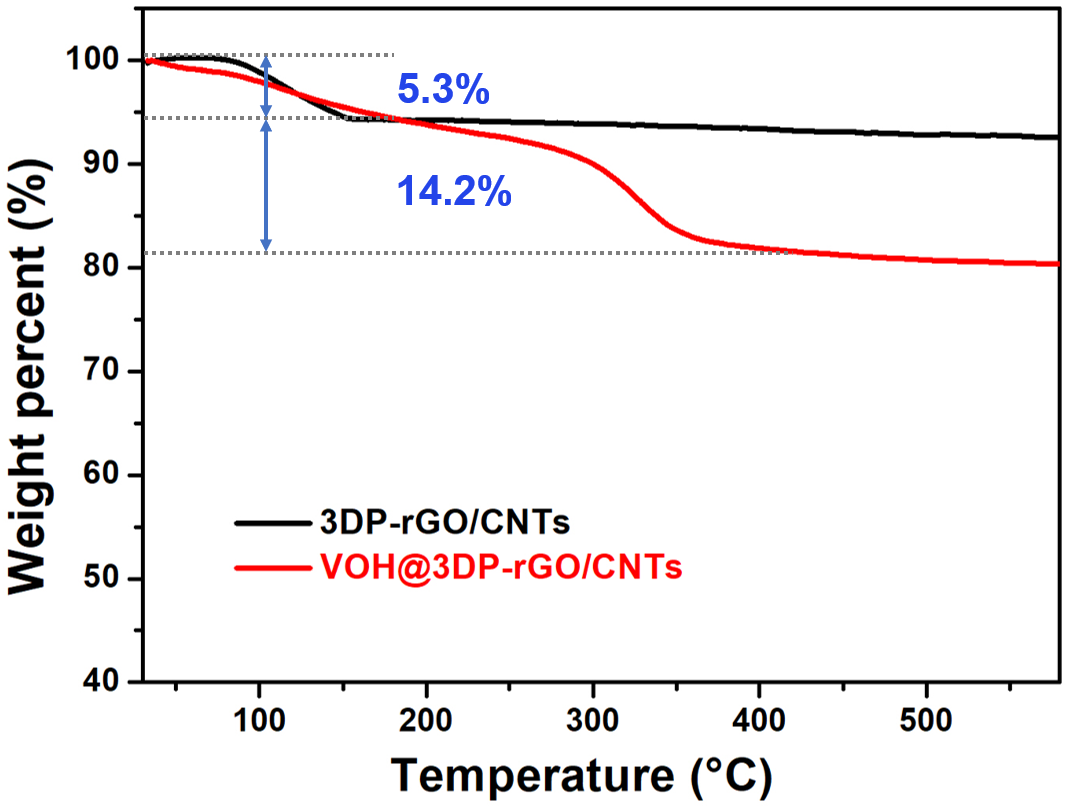
**

**Figure S23**. TG analysis of the as-prepared VOH in Ar atmosphere from 30 °C to 580 °C with a heating rate of 10 °C min^-1^. The initial ~5.3% weight loss is attributed to the loss of physically absorbed water. Additional weight loss of 14.2% mainly corresponds to the loss of crystal water.

The water loss of the as-prepared VOH@3DP-rGO/CNTs sample was determined by the thermogravimetric analysis (TGA), and the weight loss curve exhibited two-slope curves (Figure S23), ascribing to the two weight loss steps of absorbed water and structural water, and the ratio of absorbed water and structural water are 5.3% and 14.2%, respectively.

**
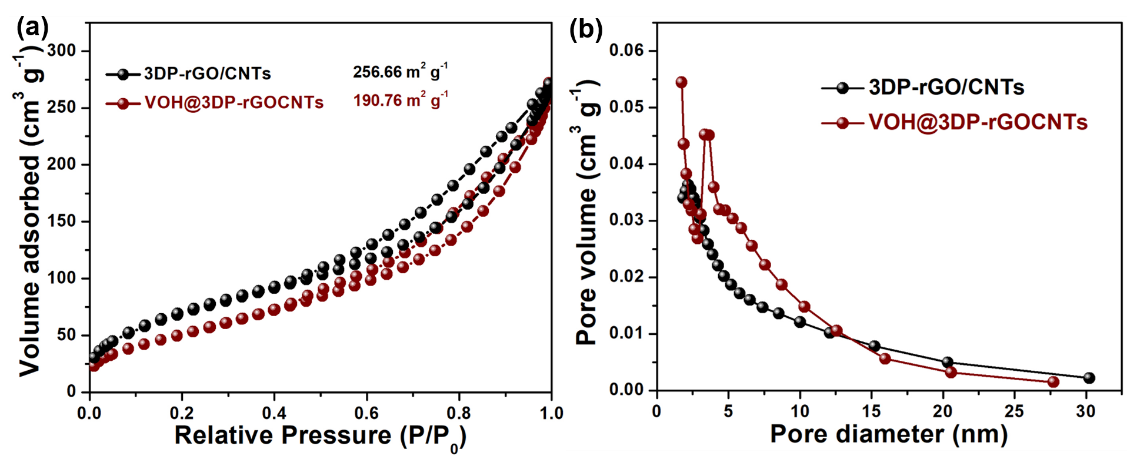
**

**Figure S24**. (a) Barrett-Joyner-Halenda (BJH) adsorption pore-size distribution for the obtained 3D-rGO/CNTs and VOH@3DP-rGO/CNTs microlattices. (b) Nitrogen adsorption-desorption isotherm.

The Brunauer Emmett Teller (BET) method further explains the changes in porosity and specific surface area of the as-prepared 3D-rGO/CNTs and VOH@3DP-rGO/CNTs samples, as shown in Figure S24. The surface areas are 256.66 and 190.76 m^2^ g^-1^ for the 3DP-rGO/CNTs and VOH@3DP-rGO/CNTs electrodes, respectively (Figure S24a). Meanwhile, the corresponding pore sizes of the both samples are ~2.1 nm and ~3.4 nm, respectively (Figure S24b). The growth of VOH NSAs may lead to the blockage of some micropores and mesopores, and making the pore size increases, which ultimately reduces the exposed specific surface area. Therefore, an optimal electrodeposition time of VOH NSAs in the 3DP-rGO/CNTs aerogel microlattice may be adjusted to achieve the best electrochemical performance.


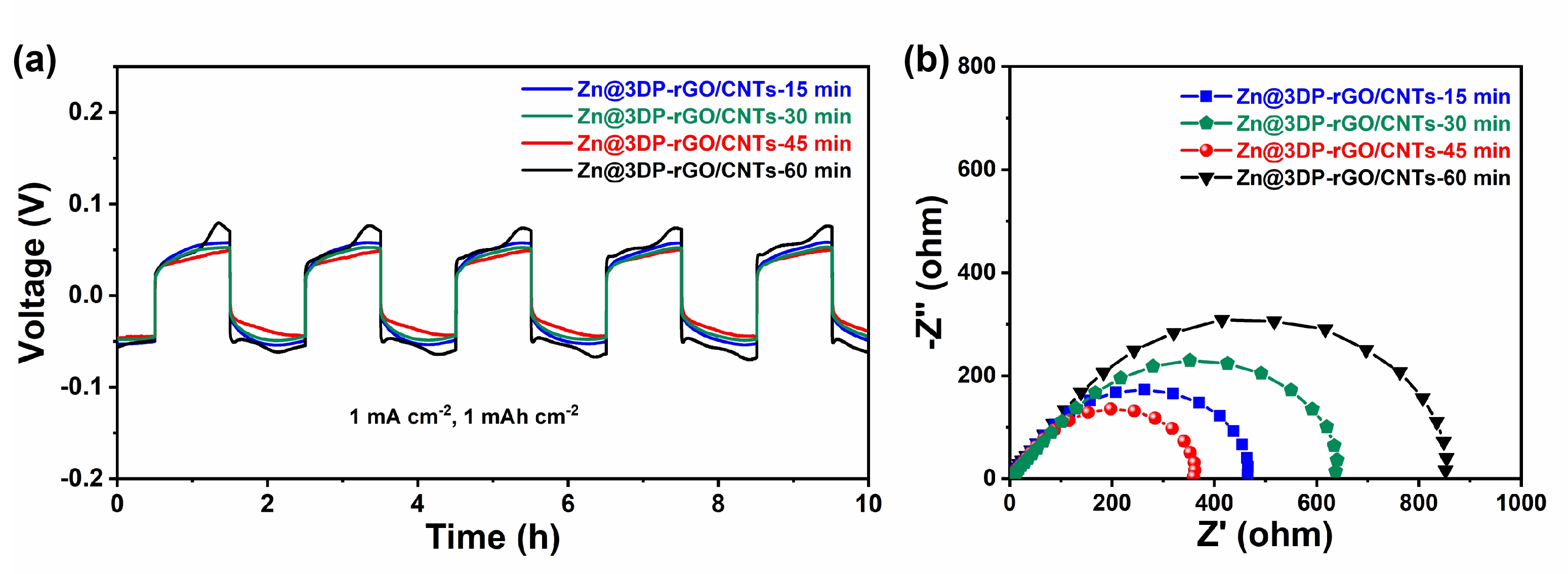


**Figure S25**. (a) Galvanostatic Zn stripping/plating behavior in a Zn/Zn symmetrical cell (based on Zn@3DP-rGO/CNTs electrodes with different growth time (15~60 min)) using the aqueous 2 M ZnSO_4_ electrolyte at 1 mA cm^-2^ with an areal capacity of 1 mAh cm^-2^. (b) The EIS of Zn@3DP-rGO/CNTs electrodes with different growth time (15~60 min) in the frequency range of 100 mHz to 100 kHz.

**
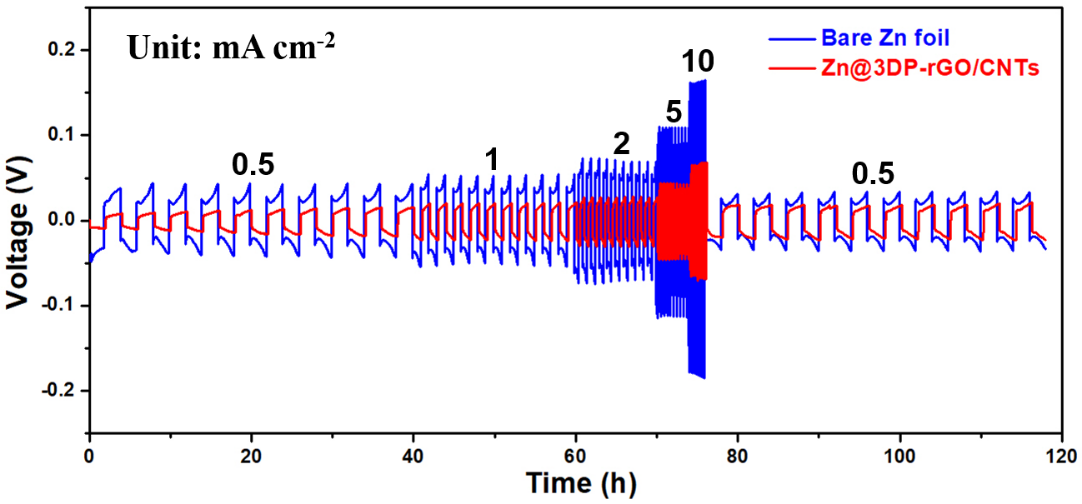
**

**Figure S26**. Rate performances of bare Zn foil and Zn@3DP-rGO/CNTs at different current densities with the capacity of 1 mAh cm^-2^.


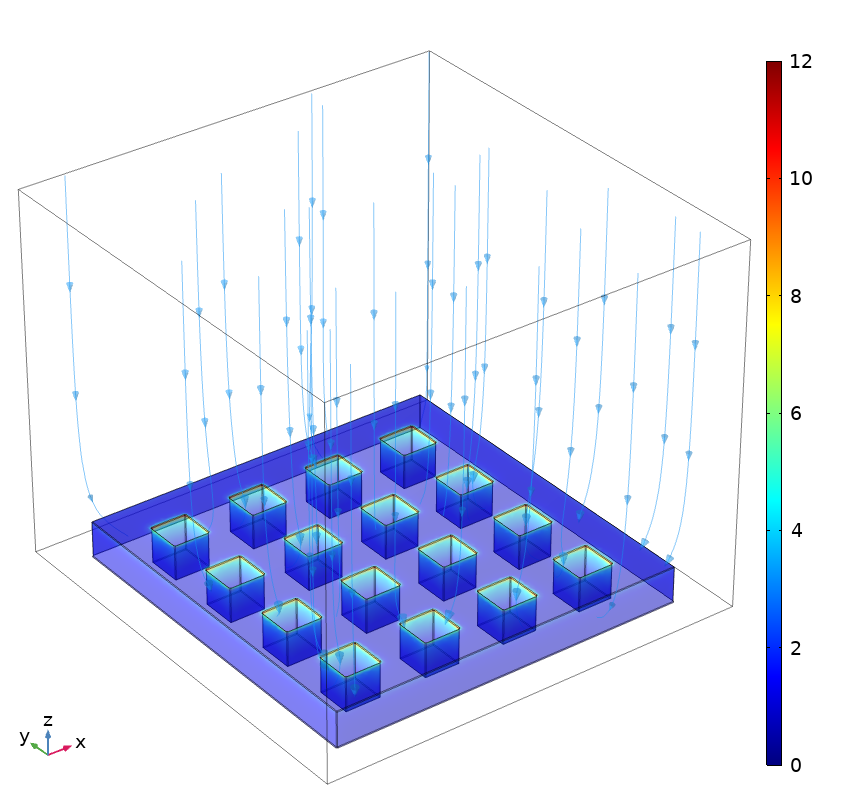


**Figure S27**. Simulation of the current density distribution of the electrolyte at the interface of Zn@3DP-rGO/CNTs microlattices electrodes by COMSOL Multiphysics software.

**
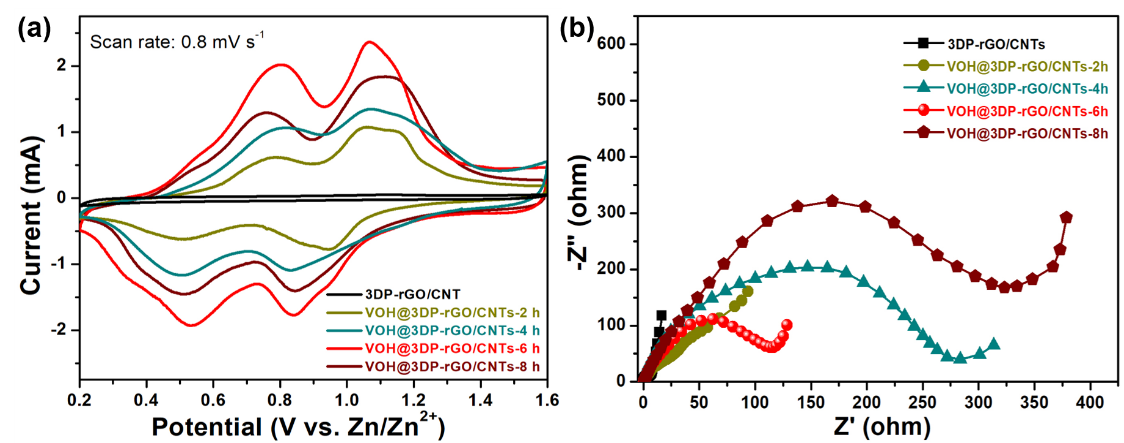
**

**Figure S28**. The CV curves of 3DP-rGO/CNTs and VOH@3DP-rGO/CNTs with different growth time electrodes at a scan rate of 0.8 mV s^-1^. (b) The EIS of 3DP-rGO/CNTs and VOH@3DP-rGO/CNTs with different growth time electrodes in the frequency range of 100mHz to 100 kHz.

**
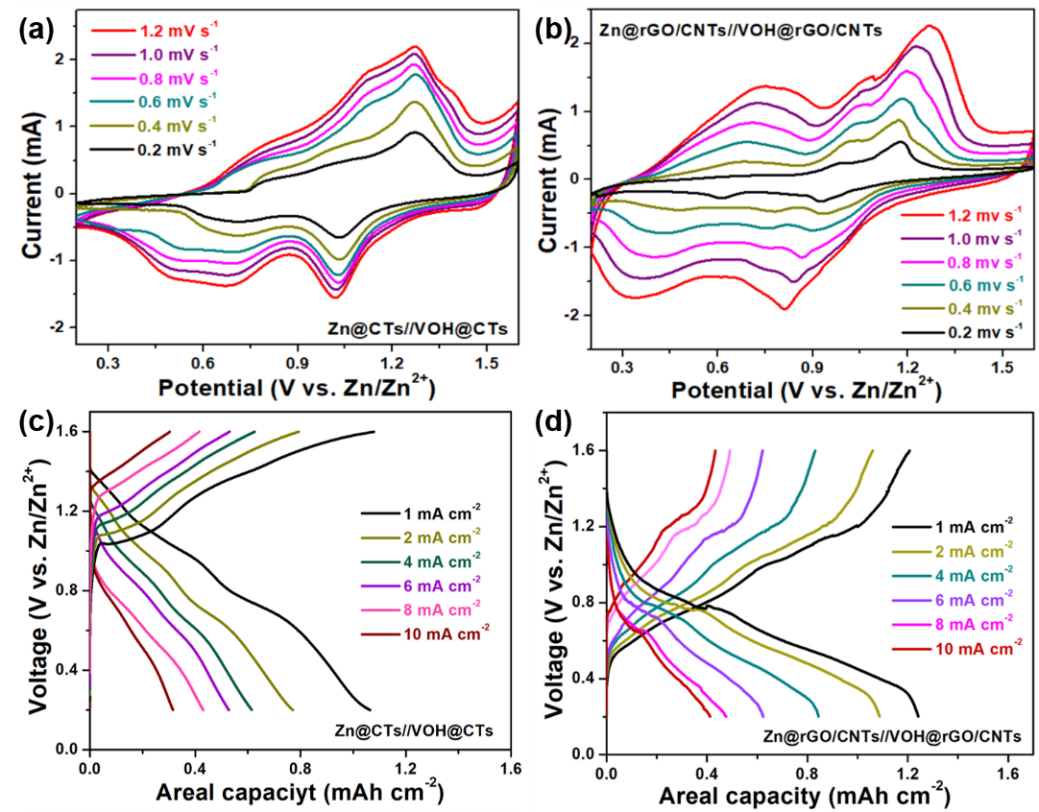
**

**Figure S29**. CV curves of the VOH@CTs and (b)VOH@rGO/CNTs cathodes at different scan rates; Galvanostatic charging-discharging profiles of the (c)VOH@CTs and (d)VOH@rGO/CNTs cathodes at various current densities.


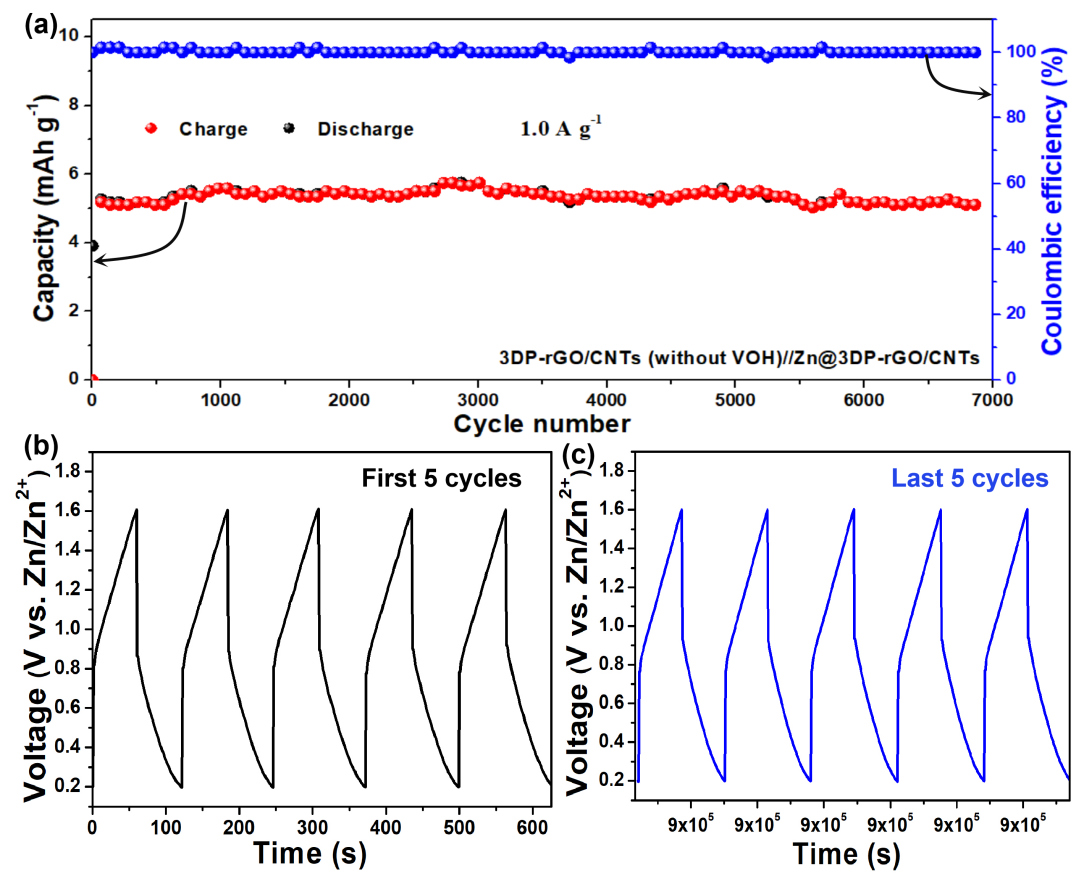


**Figure S30**. (a) Cycling properties of the 3DP-rGO/CNTs//Zn@3DP-rGO/CNTs cell device with gel electrolyte for ~7000 cycles at 1.0 A g^-1^. The galvanostatic charge/discharge (GCD) curves for (b) the first five cycles and (c) the last five cycles, respectively.

**
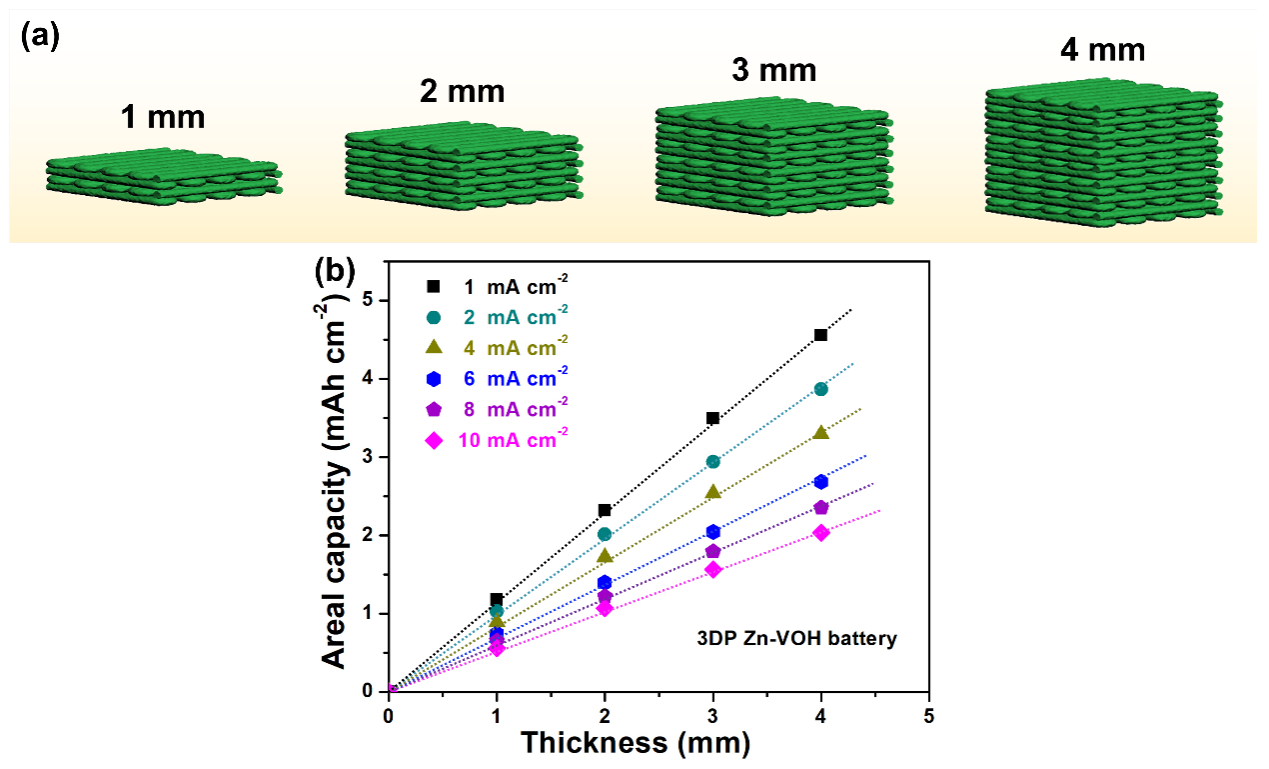
**

**Figure S31**. (a) Schematic illustration of VOH@3DP-rGO/CNTs electrodes with different thicknesses and mass; (b) Areal capacitance of the electrodes measured at 1-10 mA cm^-2^ are plotted as a function of electrode thickness.

**
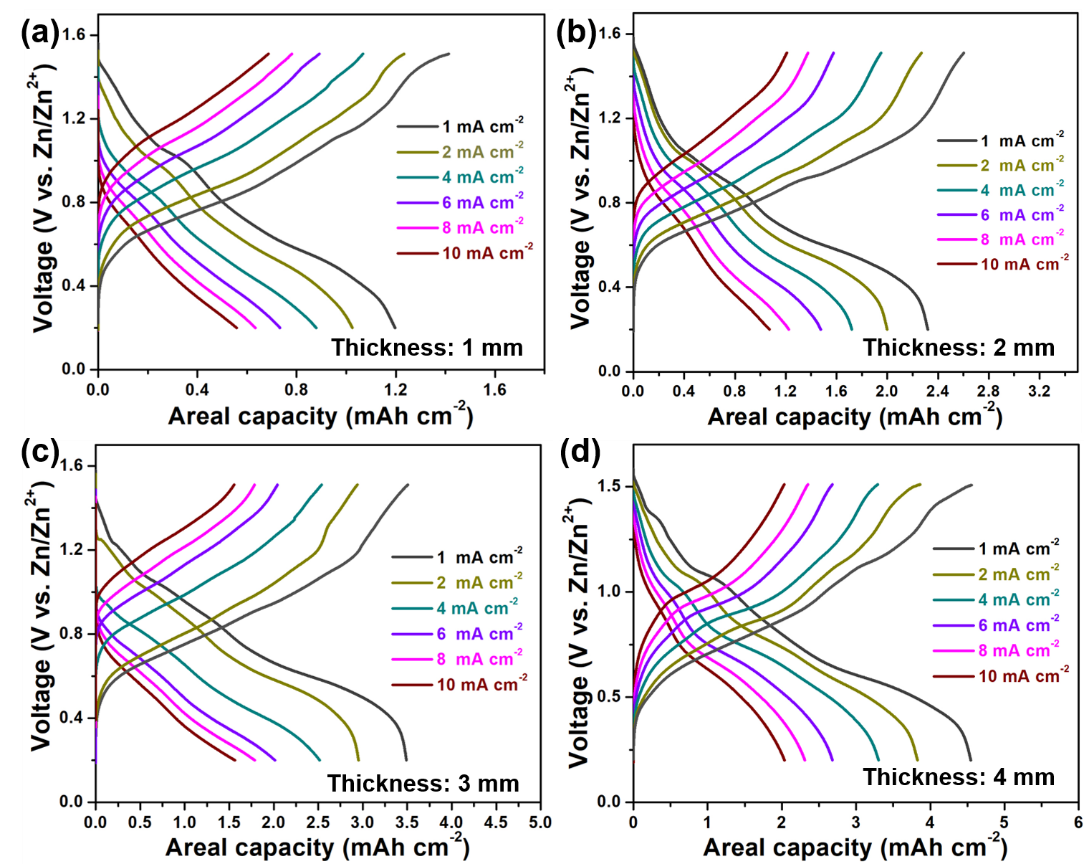
**

**Figure S32**. Specific capacity obtained at different current densities from VOH@3DP-rGO/CNTs electrodes measured at 1-10 mA cm^-2^ with different thickness (1-4 mm).

**
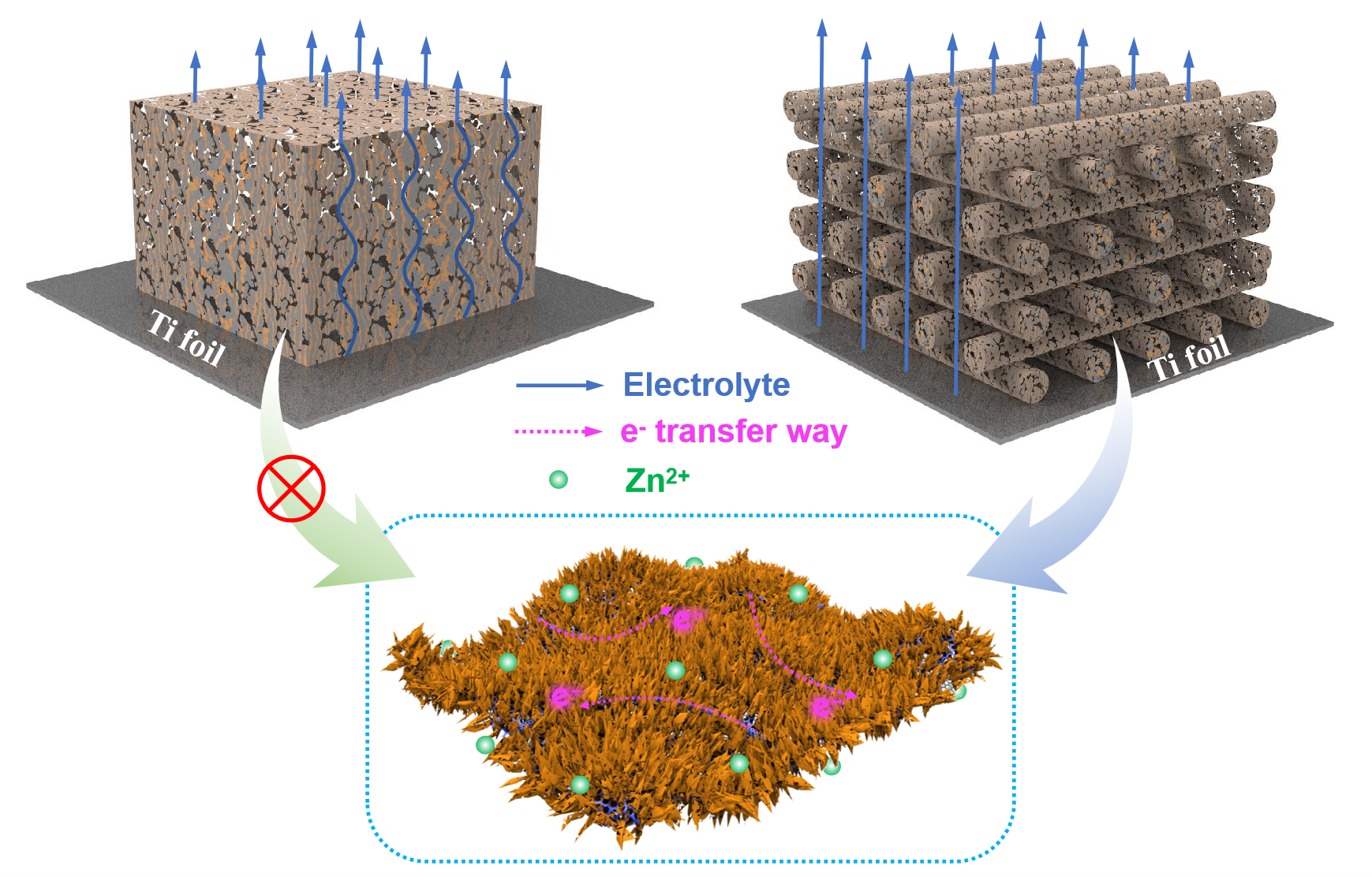
**

**Figure S33**. Schematic illustration of the mass transport process in conventional slurry coated electrode and 3D printing microlattices electrodes.


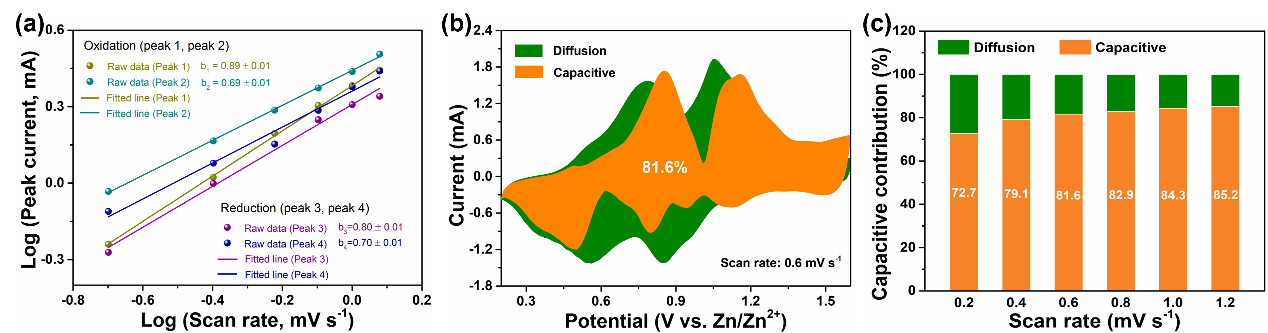


**Figure S34**. (a) log i versus log v plots according to the CV data at selected oxidation/reduction states. (b) CV curve displaying the capacitive contribution (gray region) to the total current at 0.6 mV s^-1^. (c) CV curve displaying the capacitive contribution (orange and (f) the corresponding discharge profiles at different current densities.





**Figure S35**. The evolution of R_ct_ and R_s_ in different discharge/charge states.


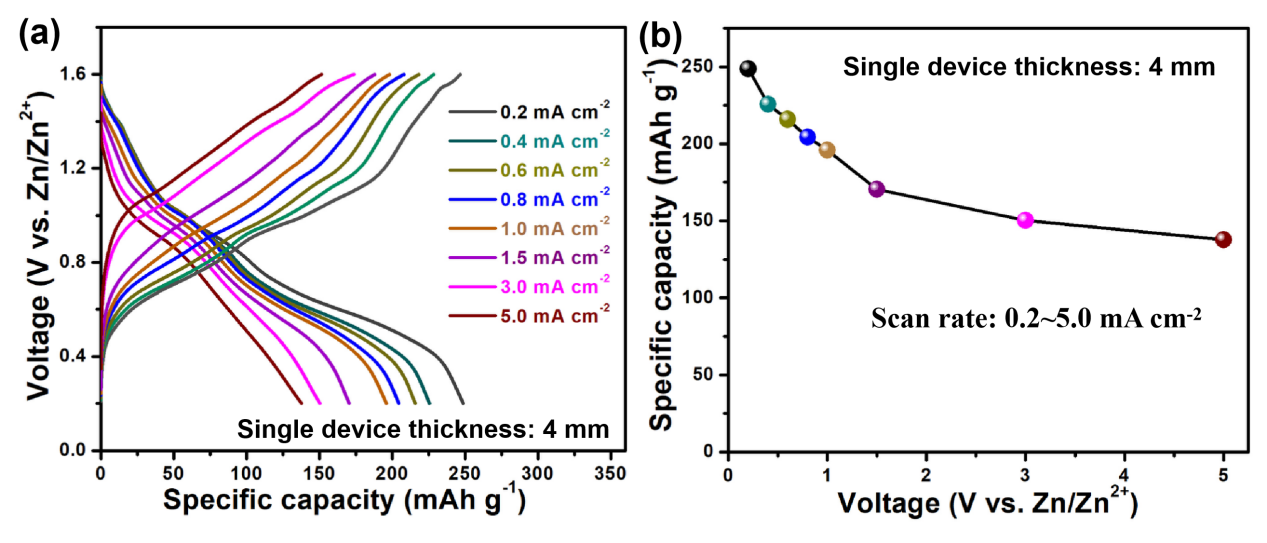


**Figure S36**. Specific capacity obtained at different current densities of 0.2-5.0 mA cm^-2^ from single Zn-VOH cell device with 4 mm thickness.


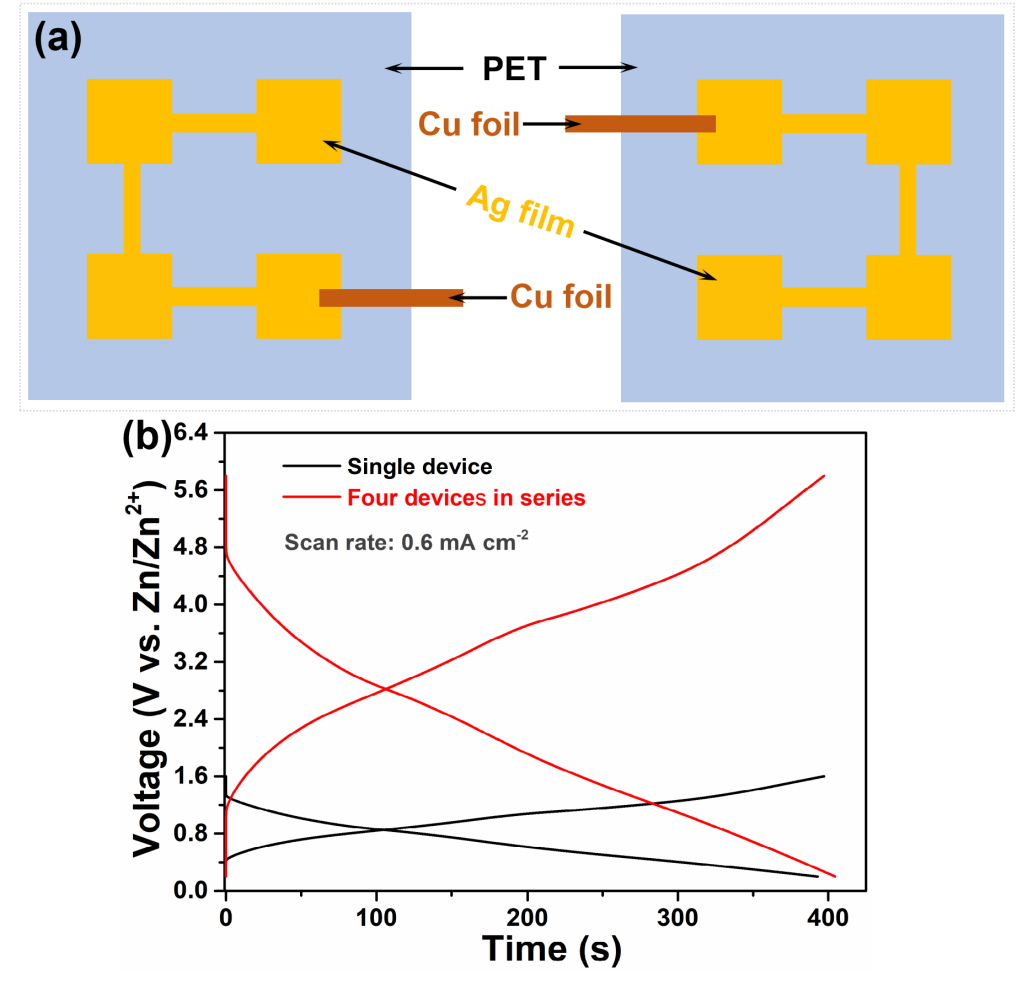


**Figure S37**. (a) Ag film patterns on both PET and glass substrates for assembling four

3D printed Zn-VOH cell devices into one unit in series. (b) The galvanostatic charge/discharge curves of four 3D printed Zn-VOH cell devices group and a single 3D printed Zn-VOH cell device at 0.6 mA cm^-2^.


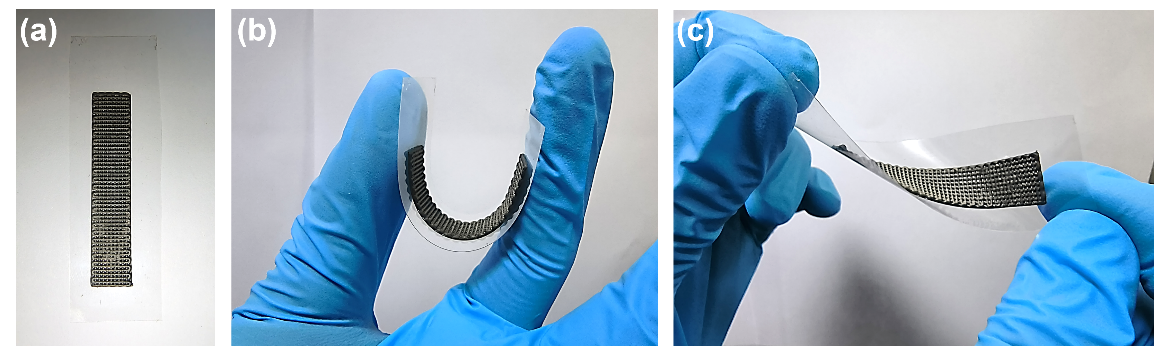


**Figure S38**. Optical images of bending and torsion tests on electrode sheets printed on PET.


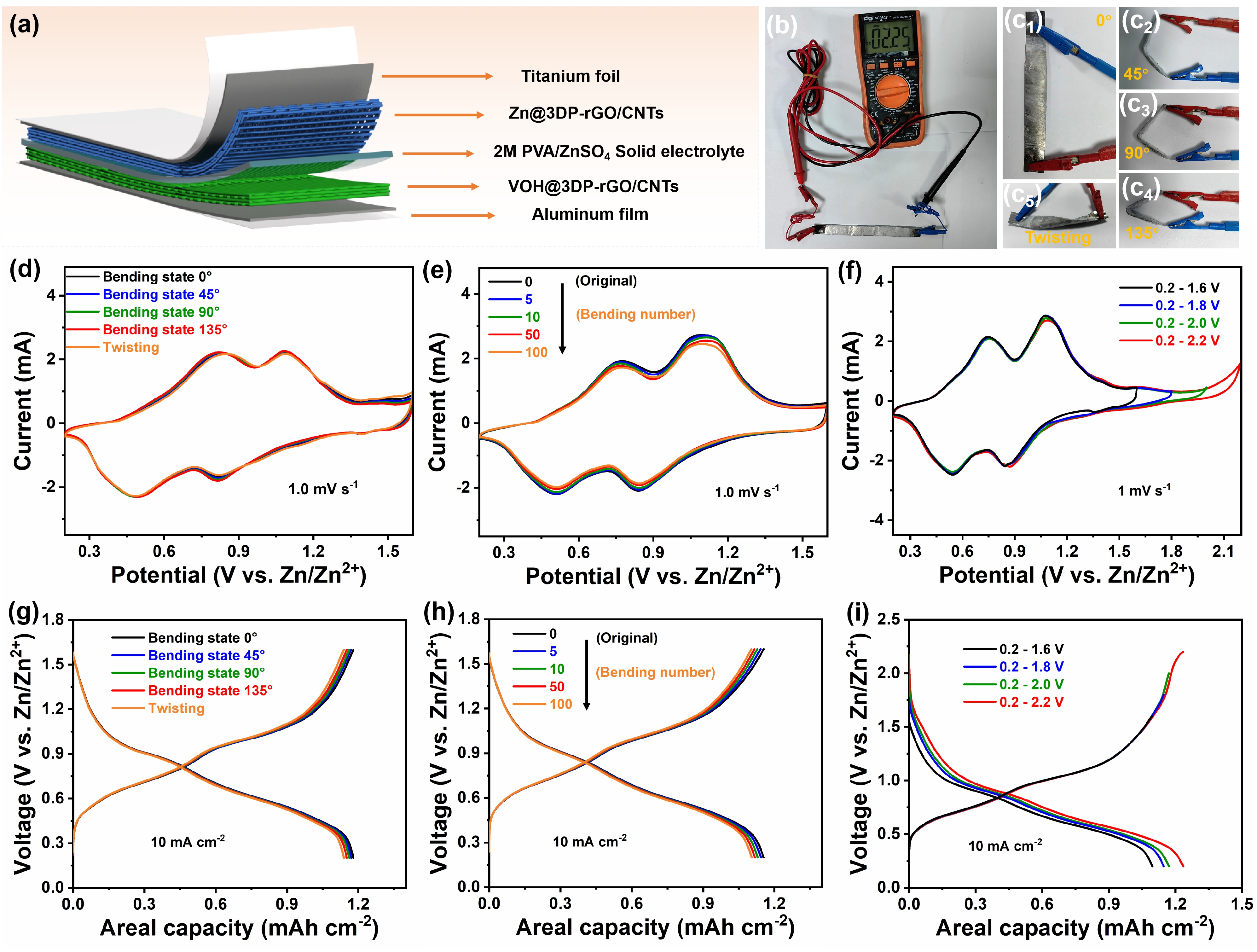


**Figure S39**. (a) Schematic of the components of the flexible 3D printed quasi-solid-state Zn-VOH battery device. (b) Two 3D printed quasi-solid-state Zn-VOH battery devices in series can reach a voltage of 2.25 V. (c) Optical photographs of the single 3D printed quasi-solid-state Zn-VOH battery device at different bending states. (d) CV curves from the single 3D printed quasi-solid-state Zn-VOH battery device taken at 1 mV s^-1^ at different bending angles. (e) CV curves of the single 3D printed quasi-solid-state Zn-VOH battery device at different numbers of sustained bends at 1 mV s^-1^. (f) CV curves of the single 3D printed quasi-solid-state Zn-VOH battery device at different voltage intervals at 1 mV s^-1^. (g) GCD curves of the single 3D printed quasi-solid-state Zn-VOH battery device for different bending angles at 10 mA cm^-2^. (h) GCD curves of the single 3D printed quasi-solid-state Zn-VOH battery device at different numbers of sustained bends at 10 mA cm^-2^. (i) GCD curves of the single 3D printed quasi-solid-state Zn-VOH battery device at different voltage intervals at 10 mA cm^-2^.


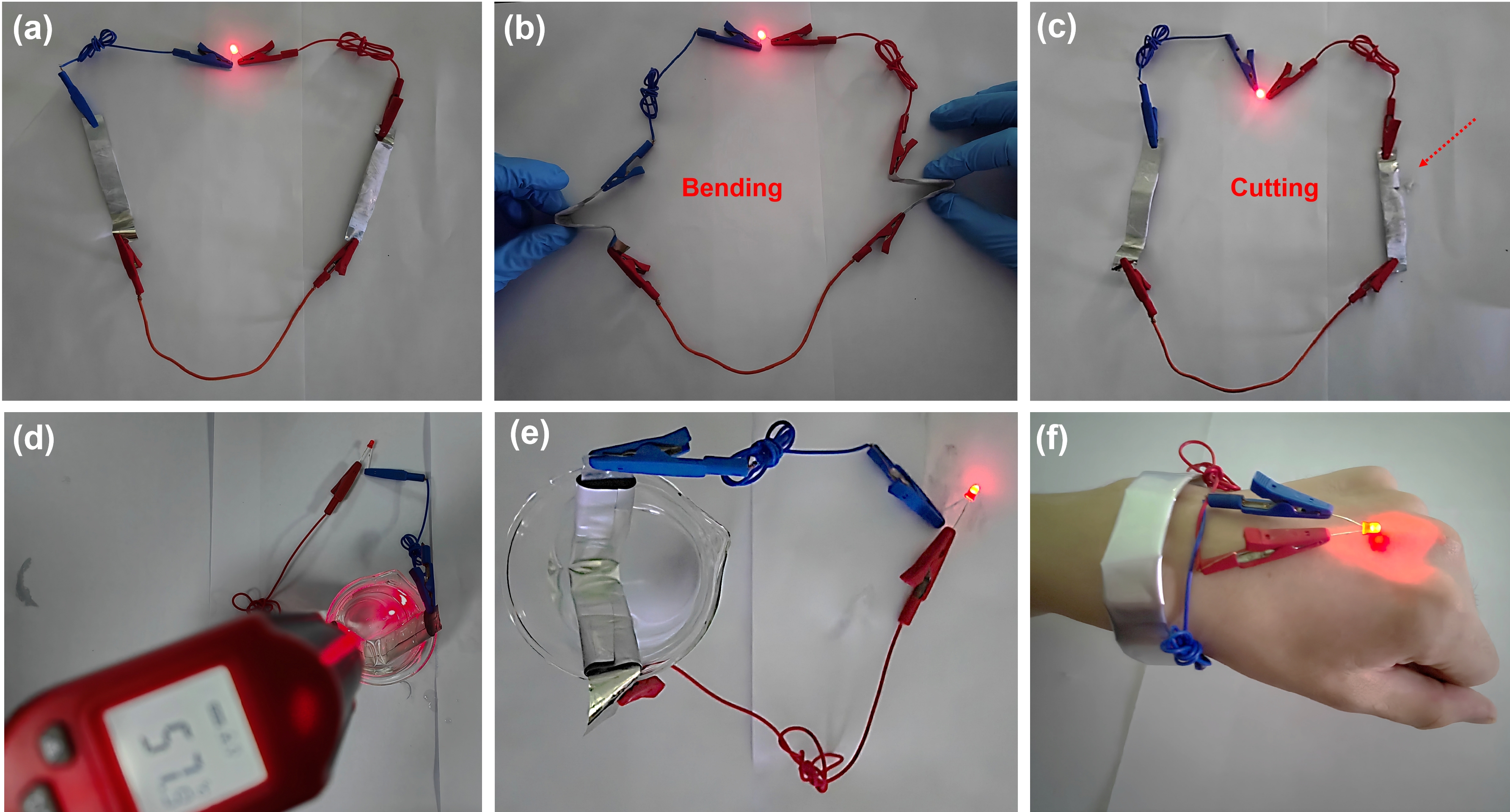


**Figure S40**. (a-c) The flexible 3D printed quasi-solid-state Zn-VOH battery device successfully power LEDs under various conditions, including bending and cutting. (d, e) The flexible 3D printed quasi-solid-state Zn-VOH battery device can successfully power a red LED at extreme temperatures of around 60 °C. (f) A ribbon-like flexible 3D printed quasi-solid-state Zn-VOH battery worn on the wrist, and can lights up a red LED.

**Table S1**. Comparison of the electrochemical performance of the 3D printed Zn-VOH battery (thick: 2 mm) with other reported aqueous Zn-ion batteries.

| Zn-ion cell devices | Electrochemical Performance | Cycle life | Ref. |
| --- | --- | --- | --- |
| VOH@3DP-rGO/CNTs//  Zn@3DP-rGO/CNTs | 364.5 W h kg^-1^ at 700 W kg^-1^ | 6500 cycles at 1.0 A g^-1^ | This Work |
| PVO//3D Ni–Zn | 260 W h kg^-1^ at 14.2 W kg^-1^ | 1000 cycles at 10.0 A g^-1^ | [13] |
| PANI@CC//3DP-ZA | 110 W h kg^-1^ at 86.2 W kg^-1^ | 1000 cycles at 1.0 A g^-1^ | [14] |
| V_2_O_5_·nH_2_O//Zn foil | 270 W h kg^-1^ at 210 W kg^-1^ | 900 cycles at 6.0 A g^-1^ | [15] |
| 3D-NVO//Zn foil | 321 W h kg^-1^ at 66.18 W kg^-1^ | 3000 cycles at 10.0 A g^-1^ | [16] |
| Zn_3_V_2_O_7_(OH)_2_·2H_2_O//Zn foil | 160 Wh kg^-1^ at 40 W kg^-1^ | 300 cycles at.0.2 A g^-1^ | [17] |
| Zn_0.25_V_2_O_5_·nH_2_O//Zn foil | 245 Wh kg^-1^ at 40 W kg^-1^ | 390 cycles at 0.056 A g^-1^ | [18] |
| ZnMn_2_O_4_//Zn foil | 202 Wh kg^-1^ at 67.5 W kg^-1^ | 1500 cycles at 1.0 A g^-1^ | [19] |
| ZnHCF//Zn foil | 102 Wh kg^-1^ at 204 W kg^-1^ | 2110 cycles at 0.05 A g^-1^ | [20] |
| H_2_V_3_O_8_//Zn foil | 245 W h kg^-1^ at 210 W kg^-1^ | 2000 cycles at 6 A g^-1^ | [21] |
| VS_2_//Zn foil | 123 W h kg^-1^ at 33 W kg^-1^ | 200 cycles at 0.5 A g^-1^ | [22] |
| VO_2_//Zn foil | 297 W h kg^-1^ at 180 W kg^-1^ | 1000 cycles at 10 A g^-1^ | [23] |
| Na_3_V_2_(PO_4_)_3_//Zn foil | 98.7 W h kg^-1^ at 625 W kg^-1^ | 100 cycles at 0.05 A g^-1^ | [24] |
| NTP/NVP//Zn foil | 65 W h kg^-1^ at 120 W kg^-1^ | 100 cycles at 1.2 A g^-1^ | [25] |
| N_3_VPF//CFF-Zn | 97.5 W h kg^−1^ at 128 W kg^-1^ | 4000 cycles at 1.0 A g^-1^ | [26] |
| NaTi_2_(PO_4_)_3_/C//Zn foil | 50 W h kg^-1^ at 30 W kg^-1^ | 500 cycles at 0.3 A g^-1^ | [27] |
| PANI-VOH//Zn foil | 216 W h kg^-1^ at 252 W kg^-1^ | 800 cycles at 3.0 A g^-1^ | [28] |

**References:**

[1] D. Jin, Y. Guo, X. Li, Y. Yang, Y. Fang, *Tunn. Undergr. Sp. Tech.* **2023**, *134*, 105017.

[2] W.-W. Song, B. Wang, C.-N. Li, S.-M. Wang, Z.-B. Han, *J. Mater. Chem. A* **2022**, *10*, 3710.

[3] Y. Wang, Y. Zhang, J. Liu, G. Wang, F. Pu, A. Ganesh, C. Tang, X. Shi, Y. Qiao, Y. Chen, H. Liu, C. Kong, L. Li, *Energy Storage Mater.* **2020**, *30*, 412.

[4] C. Li, X. Li, Q. Yang, P. Sun, L. Wu, B. Nie, H. Tian, Y. Wang, C. Wang, X. Chen, J. Shao, *Adv. Sci.* **2021**, *8*, 2004957.

[5] Z. Liang, Y. Yao, B. Jiang, X. Wang, H. Xie, M. Jiao, C. Liang, H. Qiao, D. Kline, M. R. Zachariah, L. Hu, *Adv. Funct. Mater.* **2021**, *31*, 2102994.

[6] M. C. Li, Q. L. Wu, R. J. Moon, M. A. Hubbe, M. J. Bortner, *Adv. Mater.* **2021**, *33*, 2006052.

[7] K. Shen, H. L. Mei, B. Li, J. W. Ding, S. B. Yang, *Adv. Energy. Mater.* **2018**, *8*, 1701527.

[8] M. Du, Z. Miao, H. Li, F. Zhang, Y. Sang, L. Wei, H. Liu, S. Wang, *Nano Energy* **2021**, *89*, 106477.

[9] Z. Wang, Z. Huang, H. Wang, W. Li, B. Wang, J. Xu, T. Xu, J. Zang, D. Kong, X. Li, H. Y. Yang, Y. Wang, *ACS Nano* **2022**, *16*, 9105.

[10] B. Xiao, J. Chen, C. Hu, L. Mou, W. Yang, W. He, Z. Lu, S. Peng, J. Huang, *Adv. Funct. Mater.* **2023**, *33*, 2211679.

[11] P. Zhang, Y. Li, G. Wang, F. Wang, S. Yang, F. Zhu, X. Zhuang, O. G. Schmidt, X. Feng, *Adv. Mater.* **2019**, 31, 1806005.

[12] Q. Wang, J. Zhao, J. Zhang, X. Xue, M. Li, Z. Sui, X. Zhang, W. Zhang, C. Lu, *Adv. Funct. Mater.* **2023**, *33*, 2306346.

[13] G. Zhang, X. Zhang, H. Liu, J. Li, Y. Chen, H. Duan, *Adv. Energy Mater.* **2021**, *11,* 2003927*.*

[14] L. Zeng, J. He, C. Yang, D. Luo, H. Yu, H. He, C. Zhang, *Energy Storage Mater.* **2023**, *54*, 469.

[15] M. Yan, P. He, Y. Chen, S. Wang, Q. Wei, K. Zhao, X. Xu, Q. An, Y. Shuang, Y. Shao, K. T. Mueller, L. Mai, J. Liu, J. Yang, *Adv. Mater.* **2018**, *30*, 1703725.

[16] Q. Li, X. Rui, D. Chen, Y. Feng, N. Xiao, L. Gan, Q. Zhang, Y. Yu, S. Huang, *Nano-Micro Lett.* **2020**, *12*, 67.

[17] C. Xia, J. Guo, Y. Lei, H. Liang, C. Zhao, H. N. Alshareef, *Adv. Mater.* **2018**, *30*, 1705580.

[18] Y. Wang, T. Wang, S. Bu, J. Zhu, Y. Wang, R. Zhang, H. Hong, W. Zhang, J. Fan, C. Zhi, *Nat. Commun.* **2023**, *14*, 1828.

[19] S. Deng, Z. Tie, F. Yue, H. Cao, M. Yao, Z. Niu, *Angew. Chem. Int. Edit.* **2022**, *134*, e202115877.

[20] Q. Li, K. Ma, C. Hong, Z. Yang, C. Qi, G. Yang, C. Wang, *Energy Storage Mater.* **2021**, *42*, 715.

[21] Q. Pang, C. Sun, Y. Yu, K. Zhao, Z. Zhang, P. M. Voyles, G. Chen, Y. Wei, X. Wang, *Adv. Energy Mater.* **2018**, *8*, 1800144.

[22] P. He, M. Yan, G. Zhang, R. Sun, L. Chen, Q. An, L. Mai, *Adv. Energy Mater.* **2017**, *7*, 1601920.

[23] C. Liu, W. Xu, C. Mei, M. Li, W. Chen, S. Hong, W.-Y. Kim, S. Lee, Q. Wu, *Adv. Energy Mater.* **2021**, *11*, 2003902.

[24] G. Li, Z. Yang, Y. Jiang, C. Jin, W. Huang, X. Ding, Y. Huang, *Nano Energy* **2016**, *25*, 211.

[25] H. Zhang, B. Qin, J. Han, S. Passerini, *ACS Energy Lett.* **2018**, *3*, 1769.

[26] W. Li, K. Wang, S. Cheng, K. Jiang, *Energy Storage Mater.* **2018**, *15*, 14.

[27] Z. Hou, X. Li, J. Liang, Y. Zhu, Y. Qian, *J. Mater. Chem. A* **2015**, *3*, 1400.

[28] M. Wang, J. Zhang, L. Zhang, J. Li, W. Wang, Z. Yang, L. Zhang, Y. Wang, J. Chen, Y. Huang, D. Mitlin, X. Li, *ACS Appl. Mater. Inter.* **2020**, *12*, 31564.
